# Background Vlasov equations and Young measures for passive scalar and vector advection equations under special stochastic scaling limits

**DOI:** 10.1007/s00440-026-01471-3

**Published:** 2026-04-10

**Authors:** Federico Butori, Franco Flandoli, Eliseo Luongo, Yassine Tahraoui

**Affiliations:** 1 Scuola Normale Superiore, Piazza dei Cavalieri, 7, 56126 Pisa, Italia; 2 Present Address: Fakultät für Mathematik, Universität Bielefeld, 33501 Bielefeld, Germany

**Keywords:** Young Measures, Transport Noise, Stretching Noise, Passive Scalars, Magnetohydrodynamics, 60H15, 60H30, 76F25

## Abstract

In the last few years it was proved that scalar passive quantities subject to suitable stochastic transport noise, and more recently that also vector passive quantities subject to suitable stochastic transport and stretching noise, weakly converge to the solutions of deterministic equations with a diffusion term. In the background of these stochastic models, we introduce stochastic Vlasov equations which give additional information on the fluctuations and oscillations of solutions: we prove convergence to non-trivial Young measures satisfying limit PDEs with suitable diffusion terms. In the case of a passive vector field the background Vlasov equation adds completely new statistical information to the stochastic advection equation.

## Introduction

This work introduces a background stochastic Vlasov equation behind stochastic transport and advection equations which gives additional information on the fluctuations and oscillations of solutions. It is based on Young measures; although the use of Young measures in PDEs is classical, the introduction of these particular Vlasov equations seems to be new. We develop first the theory for the stochastic transport of a passive scalar, where the result is new but just enriches incrementally the existing understanding. More importantly, then we develop the theory for a passive vector field, where also stretching acts besides transport. Here the results based on the background Vlasov equation add completely new statistical information to the stochastic advection equation.

### Stochastic passive scalar

Consider the stochastic transport equation on the *d*-dimensional torus $\mathbb {T}^{d}$

1.1 $$\begin{aligned} {\left\{ \begin{array}{ll} d\theta ^n\left( t, x\right) & =\sum _{k,j}\sigma _{k,j}^{n}\left( x\right) \cdot \nabla \theta ^n\left( t,x\right) \circ dW_{t}^{k,j} \\ \theta ^n|_{t=0} &  =\overline{\theta }_{0} \end{array}\right. } \end{aligned}$$

Under suitable assumptions described below we know, since [21], that $\theta ^n$ weakly converges to $\overline{\theta }$, solution of

$$\begin{aligned} {\left\{ \begin{array}{ll} \partial _{t}\overline{\theta }\left( t,x\right) &  =\kappa _{T} \Delta \overline{\theta }\left( t, x\right) \\ \overline{\theta }|_{t=0} &  =\overline{\theta }_{0} \end{array}\right. } \end{aligned}$$

for a suitable constant $\kappa _{T}>0$. It is clear that strong convergence in $L^2$ cannot hold, since the $L^2$-norm is preserved by the stochastic transport equation and not by the limit heat equation (different is the case when a dissipation is already present in the transport equation, see the recent work [1]). The convergence is only weak; here we reinforce this fact by showing that the limit Young measure is not trivial. Indeed we prove that, for every test functions $\varphi \in C_{b}\left( \mathbb {R}\right)$, $\psi \in C_{b}\left( \mathbb {T}^{d}\right)$, for every $t\in \left[ 0,T\right]$ we have

$$ \lim _{n\rightarrow \infty }\int _{\mathbb {T}^d}\varphi \left( \theta ^n\left( t,x\right) \right) \psi \left( x\right) dx=\int _{\mathbb {T}^d}\left( \int _{\mathbb {R}}\varphi \left( \theta \right) \mu \left( t,x,d\theta \right) \right) \psi \left( x\right) dx $$

for a suitable probability measure $\mu \left( t,x,d\theta \right)$ on $\mathbb {R}$, measurably dependent on $\left( t, x\right)$, having the property

$$ \int _{\mathbb {R}}\theta \mu \left( t,x,d\theta \right) =\overline{\theta }\left( t,x\right) . $$

The probability measure $\mu \left( t,x,d\theta \right)$ is the Young measure associated to the weak convergence of $\theta ^n$ to $\overline{\theta }$, and solves, in the distributional sense, the equation

1.2 $$\begin{aligned} {\left\{ \begin{array}{ll} \partial _{t}\mu \left( t,x,d\theta \right) &  =\kappa _{T}\Delta _{x}\mu \left( t,x,d\theta \right) \\ \mu \left( 0,x,d\theta \right) &  =\delta _{\overline{\theta }_0\left( x\right) }\left( d\theta \right) . \end{array}\right. } \end{aligned}$$

As shown by the example of Remark 3.1 below, this Young measure is not trivial, as it should be when strong convergence does not hold.

Locally around each point $x_0$ (choose, in the limit above, a probability density function ψ concentrated around $x_0$) the values of $\theta ^n(x,t)$ have large fluctuations/oscillations: the complex Lagrangian dynamics produced by the transport noise mixes the different values of the initial condition $\overline{\theta }_0(x)$ to such a high degree that everywhere, locally, there are high variations; before taking the limit as $n\rightarrow \infty$. Then, in the limit $n\rightarrow \infty$, these local oscillations around a point $x_0$ are summarized, at $x_0$, by the non-trivial measure $\mu \left( t,x_0,d\theta \right)$. This is just the general interpretation of Young measures but it may be convenient to recall it for the understanding of the result, in connection with the mixing properties of the flow.

The result above comes from the following fact: in the background of the stochastic transport equation above there is a stochastic Vlasov equation for measures satisfied by $\mu _n(t,x,d\theta )=\delta _{\theta ^n(t,x)}(d\theta )$. This can be easily seen by some formal manipulations, we refer to subsection 4.1 below for the rigorous argument. If $f:\mathbb {T}^d \times \mathbb {R}\rightarrow \mathbb {R}$ is a smooth function, by Itô formula it holds

$$\begin{aligned} f(x, \theta ^n(t,x))=f(x,\overline{\theta }_0(x))+\sum _{k,j}\int _0^t \partial _{\theta } f(x,\theta ^n(s,x))\sigma _{k,j}^n(x)\cdot \nabla \theta ^n(s,x) \circ dW^{k,j}_s. \end{aligned}$$

Integrating the relation above on $\mathbb {T}^d$, by the definition of $\mu ^n$, we obtain

$$\begin{aligned}&\int _{\mathbb {T}^d\times \mathbb {R}} f(x,\theta ) \mu _n(t,x,d\theta )dx= \int _{\mathbb {T}^d\times \mathbb {R}} f(x,\theta )\delta _{\overline{\theta }_{0}(x)} (d\theta ) dx\\  &\quad -\sum _{k,j}\int _0^t\int _{\mathbb {T}^d} \int _{\mathbb {R}} \sigma _{k,j}^{n}\left( x\right) \cdot \nabla _xf( x,\theta )\mu _n(s,x,d\theta ) dx \circ dW_{s}^{k,j}. \end{aligned}$$

Hence $\mu _n(t,x,d\theta )$ satisfies, in the distributional sense, the Vlasov type SPDE

$$\begin{aligned} {\left\{ \begin{array}{ll} d\mu _{n}\left( t,x,d\theta \right) & =\sum _{k,j}\operatorname {div}_x\left( \sigma _{k,j}^n\left( x\right) \mu _{n}\left( t,x,d\theta \right) \right) \circ dW_{t}^{k,j} \\ \mu _{n}\left( 0,x,d\theta \right) &  =\delta _{\overline{\theta }_0\left( x\right) }\left( d\theta \right) \end{array}\right. } \end{aligned}$$

and $\mu \left( t,x,d\theta \right)$ is the weak limit of $\mu _{n}\left( t,x,d\theta \right)$. It is the Vlasov equation of the dynamics

$$\begin{aligned} dX_{t}^{x,n}&+\sum _{k,j}\sigma _{k,j}^{n}\left( X_{t}^{x,n}\right) \circ dW_{t}^{k}=0\\ \frac{d\theta _{t}^{x,n}}{dt}&=0 \end{aligned}$$

which represents the Lagrangian view of the transport equation, where

$$ \theta _{t}^{x,n}=\theta ^n\left( t, X_{t}^{x,n}\right) . $$

Let us remark that in homogenization theory there are several results about the diffusion limit, see for instance [27]. However they always require a dissipation in the original transport equation, hence it is not clear that one can prove for such models a result like the one above for the purely-transport stochastic problem. This result is closely related to the fundamental findings of [21] and primarily highlights the local oscillatory behavior of $\theta ^n(t,x)$; see also the discussion following Theorem 2.7 and Remark 3.3 below.

### Stochastic passive vector field

A more striking novelty arises in the case of vector advected quantities. Consider the passive vector equation

1.3 $$\begin{aligned} {\left\{ \begin{array}{ll} dB^{n}\left( t,x\right) & =\sum _{k,j}\left( \sigma _{k,j}^{n}\left( x\right) \cdot \nabla B^{n}\left( t, x\right) -B^{n}\left( t,x\right) \cdot \nabla \sigma _{k,j}^{n}\left( x\right) \right) \circ dW_{t}^{k,j} \\ B^{n}|_{t=0} &  =\overline{B}_{0}. \end{array}\right. } \end{aligned}$$

Very recently, in [12], it has been proved that, in a suitable weak sense, under suitable assumptions on $\left( \sigma _{k,j}^{n}\right)$, $B^{n}\left( t, x\right)$ weakly converges to $\overline{B}\left( t, x\right)$ solution of

$$\begin{aligned} {\left\{ \begin{array}{ll} \partial _{t}\overline{B} &  =\mathcal {D}\overline{B}\\ \overline{B}|_{t=0} &  =\overline{B}_{0}, \end{array}\right. } \end{aligned}$$

where $\mathcal {D}$ is a linear operator depending on the noise coefficients, typically corresponding to the Itô–Stratonovich corrector of the transport part of the noise (see, e.g., [12, Lemma 2.13] or equation (2.8) below). The Lagrangian dynamics behind the vector advection equation is given by:

$$\begin{aligned} dX_{t}^{x,n}&+\sum _{k,j}\sigma _{k,j}^{n}\left( X_{t}^{x,n}\right) \circ dW_{t}^{k,j}=0\\ {db_{t}^{x,n}}&+\sum _{k,j}b_{t}^{x,n}\cdot \nabla \sigma _{k,j}^{n}\left( X_{t}^{x,n}\right) \circ dW_{t}^{k,j}=0 \end{aligned}$$

where

$$ b_{t}^{x,n}=B^{n}\left( t, X_{t}^{x,n}\right) . $$

The associated Vlasov equation is now (we denote $\mu _{n}\left( t,x,db\right)$ simply by $\mu _{n}$)

$$\begin{aligned} {\left\{ \begin{array}{ll} d\mu _{n}=\sum _{k,j}{{\,\textrm{div}\,}}_{x}\left( \sigma _{k,j}^{n}\left( x\right) \mu _{n}\right) \circ dW_{t}^{k,j} +\sum _{k,j}{{\,\textrm{div}\,}}_{b}\left( b\cdot \nabla \sigma _{k,j}^{n}\left( x\right) \mu _{n}\right) \circ dW_{t}^{k,j} \\ \mu _{n}\left( 0,x,db\right) =\delta _{\overline{B}_0\left( x\right) }\left( db\right) . \end{array}\right. } \end{aligned}$$

In this work, we impose suitable scaling assumptions on $\left( \sigma _{k,j}^{n}\right)$ which, as shown in the equation (2.7), imply that

$$ \mathcal {D}=0, $$

namely

1.4 $$\begin{aligned} \overline{B}\left( t,x\right) =\overline{B}_{0}\left( x\right) . \end{aligned}$$

Similar scaling assumptions are also used in [20], which addresses the dynamics of polymers in turbulent fluids. We prove that, $\mu _{n}\left( t, x,db\right)$ weakly converges to $\mu \left( t,x,db\right)$, solving

1.5 $$\begin{aligned} {\left\{ \begin{array}{ll} \partial _{t}\mu &  ={{\,\textrm{div}\,}}_{b}\left( \mathcal {L}\left( b\right) \nabla _{b}\mu \right) \\ \mu \left( 0, x,db\right) &  =\delta _{\overline{B}_0\left( x\right) }\left( db\right) , \end{array}\right. } \end{aligned}$$

where the matrix-valued function $\mathcal {L}\left( b\right)$ is described in equation (2.13) below. Again we have the compatibility relations (now for $\varphi \in C_{b}\left( \mathbb {R} ^{3}\right)$, $\psi \in C_{b}\left( \mathbb {T}^{3}\right)$)

1.6 $$\begin{aligned} \lim _{n\rightarrow \infty }\int _{\mathbb {T}^3}\varphi \left( B^{n}\left( t,x\right) \right) \psi \left( x\right) dx=\int _{\mathbb {T}^3}\left( \int _{\mathbb {R}^3}\varphi \left( b\right) \mu \left( t,x,db\right) \right) \psi \left( x\right) dx \end{aligned}$$

identifying $\mu \left( t,x,db\right)$ as the Young measure associated to the sequence $\left( B^{n}\right)$ and

1.7 $$\begin{aligned} \int _{\mathbb {R}^3} b\mu \left( t,x,db\right) =\overline{B}\left( t,x\right) . \end{aligned}$$

Up to here, the interpretation of the result would be similar to the scalar case, namely that the reformulation with Young measures gives more insight about the oscillations. However, here the oscillations are not only produced by the mixing mechanism which moves different values of the initial condition close each other. Here the stretching may enhance the oscillations. One could believe that the equation for $\overline{B}$ is sufficient to capture the effect of stretching, but it is not so. On the contrary, the background Vlasov equation introduced here does. Let us show this phenomenon.

Opposite to the case of the passive scalar θ, here the equation includes second order terms in *b* which produce a regularization in the *b*-variable of the initial condition $\delta _{\overline{B}_{0}\left( x\right) }\left( db\right)$: except for b=0, the matrix $\mathcal {L}(b)$ is non-singular and yields solutions with full support in *b*; see Corollary B.6 and its consequence, Proposition B.1. In particular, for every $(x_0,t)$ and R,r>0, and for all sufficiently large *n*,

$$ \mathcal {L}_{3}\left\{ x\in U_r\left( x_{0}\right) :\left| B^{n}\left( t,x\right) \right| \ge R\right\} >0, $$

where $\mathcal {L}_3$ denotes the Lebesgue measure on $\mathbb {T}^3$ and $U_r(x_0)\subset \mathbb {T}^3$ is the ball in $\mathbb {T}^3$ centered at $x_0$ with radius r>0. Notice also that the stationary equation

$$ {{\,\textrm{div}\,}}_{b}\left( \mathcal {L}\left( b\right) \nabla _{b}f\left( b\right) \right) =0 $$

corresponding to uniform conditions in *x*, is solved by an explicit rotation-invariant distribution, a probability density function *f*(*b*). This density has full support on positive values.

To see how it is possible to obtain $\mathcal {D}=0$ and a non trivial $\mathcal {L}(b)$, we have to understand their different roles: when computing the Itô-Stratonovich corrector for (1.3) (see [12, Lemma 2.13]), we see that the operator $\mathcal {D}$, for an isotropic and space homogeneous noise as the one considered in this paper, comes only from the transport part of the noise. On the contrary, the term $\mathcal {L}(b)$ in the Vlasov equation, is due only to the presence of the stretching part of the noise (see subsection 4.1 below, in particular the proof of Lemma 4.3). Taking advantage of the scaling discrepancy between $\sigma _{k,j}^{n}$ (the transport coefficients) and $\nabla \sigma _{k,j}^{n}$ (the stretching ones), we consider coefficients with spatial oscillations of order approximately *n*, and we impose that as $n\rightarrow \infty$ the intensity of the transport noise decays to zero, while the intensity of the stretching part stays approximately constant. This has to be understood in the sense of the traces of their covariances, we refer to Remark 2.8 below for details. Hence, in this regime, we expect $\mathcal {D}=0$, but a non trivial $\mathcal {L}(b)$. With these considerations at hand, we conclude that the non-triviality of the limit Young measure does not arise from transport mixing, which is irrelevant in the limit, but only from turbulent stretching.

The interpretation of these results then is that the stochastic vector advection equation, for large *n*, due to the turbulent Lagrangian stretching produced by $\left( \sigma _{k,j}^{n}\right)$, develops wide variations (and in particular large values) of $B^{n}\left( t,x\right)$ at small scale, so that in an infinitesimal volume around any point $x_0$ there is a “density" of values of $B^{n}\left( t, x_0\right)$ (just a sort of empirical density, for finite *n*) converging in the limit to a measure $\mu \left( t,x_0,db\right)$ with a true density. For large times the distribution in *x* is uniform and this “density” of *B*-values is described by $f\left( b\right)$. In spite of the fact that in the average we have (1.4). Therefore the background Vlasov equation provides new information, not visible at the level of the vector advection equation. The correspondence of $f\left( b\right)$ with statistical properties observed for magnetic fields in turbulent plasma and other examples will be discussed elsewhere.

Let us conclude this subsection by outlining the main elements of the analysis that will be carried out in Sect. 4. First, we need to rigorously define the Vlasov dynamics associated with the stochastic equation for the magnetic field. This presents some technical difficulties due to the low regularity of several quantities appearing in the magnetic field equation, and calls for the use of regularizations and commutator estimates in the spirit of [14]. Once the equation for $\mu _n$ has been obtained, we proceed by applying a compactness argument for continuous functions taking values in the space of Young measures. To derive the necessary a priori estimates, we exploit the duality between the Vlasov and the SPDE formulations, translating $L^2$ bounds on the magnetic field into properties of the corresponding Young measure. The validity of the aforementioned $L^2$ bounds forces us to move away from the scaling limit considered in [21], where the effects of the stretching term cannot be controlled (cf. [19, Section 3.4], [18, Section 6] and [4, Section 2.2] for examples of this phenomenon). This, in turn, requires faster decaying modes in the noise, rendering the transport term negligible and implying $\mathcal {D}\overline{B}=0$. We can then pass to the limit in each term of the approximating stochastic Vlasov equations thanks to Jakubowski’s version of the Skorokhod representation theorem, and identify μ, the limit of $\mu _n$, as the unique Young-measure-valued solution of equation (1.5). Once again, the nontriviality of the differential operator appearing in (1.5) arises from the decay rate of the noise modes in our scaling limit, which is chosen so that the stretching term remains of order one: a faster decay would yield a trivial matrix $\mathcal {L}(b)$, while a slower one would cause it to diverge.

#### Plan of the paper

In Sect. 2 we provide the functional analytic setup in order to describe rigorously our main results: Theorem 2.7 and Theorem 2.11. The proof of Theorem 2.7 is the content of Sect. 3, while the proof of Theorem 2.11 is the content of Sect. 4. We conclude the paper with two appendices. Appendix A is devoted to the study of the well-posedness of the stochastic equation for the ideal magnetic field, while we add some comments on the interpretation of our results in Appendix B.

## Functional analytic setup and main results

### Notation

Let us start setting some classical notation and properties of operators in the periodic setting before describing the main contributions of this work. We refer to monographs [15, 32, 33, 35] for a complete discussion on the topics shortly recalled in this section.

Let d≥2, in the following we denote by $\mathbb {T}^d=\mathbb {R}^d/(2\pi \mathbb {Z})^d$ the *d* dimensional torus and by $\mathbb {Z}^d_0=\mathbb {Z}^d\smallsetminus \{0\}$ the *d* dimensional lattice made by points with integer coordinates. On $\mathbb {Z}^d_0$ we introduce a partition $\mathbb {Z}^d_0=\Gamma _{d,+}\cup \Gamma _{d,-}$ such that $\Gamma _{d,+}=-\Gamma _{d,-}.$ In the case of d=2 we choose

$$\begin{aligned} \Gamma _{2,+}:=\{k\in \mathbb {Z}^2_0: k_2>0\vee (k_2=0\wedge k_1>0)\}, \end{aligned}$$

with an obvious generalization for d≥3.

Given a function $g:~\mathbb {T}^d~\rightarrow ~\mathbb {R}\cup ~\{+\infty \}$ we denote by $\int _{\mathbb {T}^d}^* g(x) dx$ the outer integral of *g*, i.e.

$$\begin{aligned} \int _{\mathbb {T}^d}^* g(x) dx=\inf _{\begin{array}{c} G\ge g\\ G\ \textit{Borel measurable} \end{array}} \int _{\mathbb {T}^d} G(x) dx. \end{aligned}$$

Points of $\mathbb {T}^d\times \mathbb {R}^k$ will be denoted by bold letters. Let *a*, *b* be two positive numbers, we write a≲b if there exists a positive constant *C* such that a≤Cb and $a\lesssim _\xi b$ if we want to highlight the dependence of the constant *C* on a parameter ξ.

Let $\left( {H}^{s,p}(\mathbb {T}^d),\left\| \cdot \right\| _{{H}^{s,p}}\right) ,\ s\in \mathbb {R},\ p\in (1,+\infty )$ be the Bessel spaces of periodic functions with zero mean. In case of p=2, we simply write ${H}^{s}(\mathbb {T}^d)$ in place of ${H}^{s,2}(\mathbb {T}^d)$ and we denote by $\langle \cdot ,\cdot \rangle _{{H}^s}$ the corresponding scalar products. In case of s=0, we write ${L}^p(\mathbb {T}^d)$ instead of ${H}^{0,p}(\mathbb {T}^d)$ and in case of p=2 we neglect the subscript in the notation for the norm and the inner product. With some abuse of notation, for s>0, we denote the duality between ${H}^{-s}$ and ${H}^s$ by $\langle \cdot ,\cdot \rangle$.

A different characterization of the fractional Sobolev spaces above can be given in terms of powers of the Laplacian. Denoting by

$$\begin{aligned} \Delta : \textsf {D}(\Delta )\subset {L^2}(\mathbb {T}^d)\rightarrow {L}^2(\mathbb {T}^d), \end{aligned}$$

where $\textsf {D}(\Delta )={H}^2(\mathbb {T}^d)$, it is well known that ${\Delta }$ is the infinitesimal generator of analytic semigroup of negative type that we denote by $e^{t\Delta }: L^2(\mathbb {T}^d)\rightarrow L^2(\mathbb {T}^d)$ and moreover for each s>0, $\textsf {D}((-\Delta )^{s})$ (resp. $\left( \textsf {D}((-\Delta )^{s})\right) ^{\prime }$) can be identified with $H^{2s}(\mathbb {T}^d)$ (resp. $H^{-2s}(\mathbb {T}^d)$). The semigroup $e^{t\Delta }$ is associated to an integral kernel *p*(*t*, *x*) such that

$$\begin{aligned} e^{t\Delta }u_0(x)=\int _{\mathbb {T}^d}p(t,x-y)u_0(y)dy. \end{aligned}$$

Similarly, we introduce the Bessel spaces of vector fields

$$\begin{aligned} {H}^{s,p}(\mathbb {T}^d;\mathbb {R}^d)&= \{u=(u^1,\dots , u^d)^t:\ u^1,\dots , u^d\in {H}^{s,p}(\mathbb {T}^d)\},\\ \langle u, v\rangle _{{{H}^s}}&=\sum _{j=1}^d\langle u^j,v^j\rangle _{{H}^s}, \quad \text {for } s\in \mathbb {R}. \end{aligned}$$

Again, in case of s=0 we write ${L}^p(\mathbb {T}^d;\mathbb {R}^d)$ instead of ${H}^{0,p}(\mathbb {T}^d;\mathbb {R}^d)$ and we neglect the subscript in the notation for the norm and the scalar product in case of p=2. Lastly we denote by $\textbf{H}^{s}$ the closed subspace of ${H}^s(\mathbb {T}^d;\mathbb {R}^d)$ made by zero mean, divergence free vector fields with norm induces by ${H}^s(\mathbb {T}^d;\mathbb {R}^d)$ and in order to ease the notation we, simply, write

$$\begin{aligned} H=\textbf{H}^0,\ V=\textbf{H}^1. \end{aligned}$$

We conclude this subsection introducing some classical notation when dealing with stochastic processes taking values in separable Hilbert spaces. Let $(\Omega ,\mathcal {F},\mathcal {F}_t,\mathbb {P})$ be a filtered probability space and *Z* be a separable Hilbert space, with associated norm $\Vert \cdot \Vert _{Z}$. In the following we will always assume, even if not specified, that $(\Omega ,\mathcal {F},\mathbb {P})$ is a complete probability space and $(\mathcal {F}_t)_{t\ge 0}$ is a right continuous filtration such that $\mathcal {F}_0$ contains every $\mathbb {P}$ null subset of Ω. We denote by $C_{\mathcal {F}}\left( \left[ 0,T\right] ;Z\right)$ (resp. $C_{\mathcal {F}}\left( \left[ 0,T\right] ;Z_w\right)$) the space of continuous (resp. weakly continuous) adapted processes $\left( X_{t}\right) _{t\in \left[ 0,T\right] }$ with values in *Z* such that

$$ \mathbb {E} \bigg [ \sup _{t\in \left[ 0,T\right] }\left\| X_{t}\right\| _{Z}^{2}\bigg ] <\infty $$

and by $L_{\mathcal {F}}^{p}\left( 0,T;Z\right) ,\ p\in [1,\infty ),$ the space of progressively measurable processes $\left( X_{t}\right) _{t\in \left[ 0,T\right] }$ with values in *Z* such that

$$ \mathbb {E} \bigg [ \int _{0}^{T}\left\| X_{t}\right\| _{Z}^{p}dt \bigg ] <\infty . $$

### Some preliminaries on young measures

Let us denote by $\mathcal {P}$ the set of probability measures on $\mathbb {T}^d\times \mathbb {R}^k$. We recall here some standard facts on probability measures on $\mathbb {T}^d\times \mathbb {R}^k$; we refer to [23, Section 1.1] and [2, Section 5.1] for details. Let us start denoting by $\{\textbf{x}^n\}_{n\in \mathbb {N}}$ a dense set of $\mathbb {T}^d\times \mathbb {R}^k$ and

$$\begin{aligned} \mathcal {A}&=\{(a-b|\textbf{x}-\textbf{x}^n|)\vee c,\quad a,b,c\in \mathbb {Q},\ b\ge 0,\ n\in \mathbb {N}\},\\ \textbf{A}&=\{f_1\vee f_2\vee \dots \vee f_n,\quad f_i\in \mathcal {A},\ n\in \mathbb {N}\}\\ \mathcal {G}&=\{g\in \textbf{A}\cup (-\textbf{A}): \left\| g\right\| _{C_b(\mathbb {T}^d\times \mathbb {R}^k)}\le 1\}. \end{aligned}$$

The set $\mathcal {G}$ is countable, therefore introducing an enumeration of its elements $\{ g_i\}_{i\in \mathbb {N}}$ and the following distance on the probability measures on $\mathbb {T}^d\times \mathbb {R}^k$

$$\begin{aligned} \rho (\mu ,\nu )=\sum _{i\in \mathbb {N}}\frac{\left\lvert \int _{\mathbb {T}^d\times \mathbb {R}^k} g_i(\textbf{x}) (\mu -\nu )(d\textbf{x}) \right\rvert }{2^{i}}, \end{aligned}$$

it holds:

#### Theorem 2.1

The weak topology on $\mathcal {P}(\mathbb {T}^d\times \mathbb {R}^k)$ is induced by the distance ρ.

#### Remark 2.2

Due to our definition, the functions $g_i\in \mathcal {G}$ are uniformly continuous on $\mathbb {T}^d\times \mathbb {R}^k$, since

$$\begin{aligned} g_i=\pm (f_1\vee \dots \vee f_n ) \end{aligned}$$

for some $n\in \mathbb {N}$ and $\{f_j\}_{j\in \{1,\dots ,n\}}\subset \mathcal {A}$. In particular each $f_j$ is constant outside a certain ball $U_j\subset \mathbb {T}^d\times \mathbb {R}^k$. Since *n* is finite, this implies that also *g* is constant outside $U=\cup _{j=1}^n U_j$, from which the required uniform continuity of $g_i$ follows.

Secondly let us denote by $\mathcal {Y}$ the subset of non negative Borel measures on $\mathbb {T}^d\times \mathbb {R}^k$ given by the Young measures on $\mathbb {T}^d\times \mathbb {R}^k$. We refer to [3, Chapter 4.3] and [36] for a complete discussion on the topic of Young measures. We recall that $\mu \in \mathcal {Y}$ if it is non negative and for each $A\subset \mathbb {T}^d$ Borel measurable, then

$$\begin{aligned} \mu (A\times \mathbb {R}^k)=\mathcal {L}_d(A), \end{aligned}$$

where $\mathcal {L}_d$ is the Lebesgue measure on $\mathbb {T}^d$. The latter, by disintegration theorem for measures, implies that there exists a family of probability measures on $\mathbb {R}^k$ parametrized by $x\in \mathbb {T}^d$, μ(x,db), such that for each $f\in C_b(\mathbb {T}^d\times \mathbb {R}^k)$

$$\begin{aligned} \int _{\mathbb {T}^d\times \mathbb {R}^k} f(x,b) d\mu (x,b)=\int _{\mathbb {T}^d\times \mathbb {R}^k} f(x,b) \mu (x,db)dx \end{aligned}$$

and the map $x\rightarrow \mu (x,db)$ is $\text {weakly}^*$ measurable. Given $\mu _n,\ \mu \in \mathcal {Y}$ we say that $\mu _n$ converges to μ in the sense of Young measures (shortly $\mu _n{\mathop {\rightarrow }\limits ^{\mathcal {Y}}}\mu$), if $\forall f\in C_b(\mathbb {T}^d\times \mathbb {R}^k)$

$$\begin{aligned} \int _{\mathbb {T}^d\times \mathbb {R}^k}f(x,b)\mu _n(x,db)dx\rightarrow \int _{\mathbb {T}^d\times \mathbb {R}^k}f(x,b)\mu (x,db)dx. \end{aligned}$$

Since the measure of the *d* dimensional torus is finite, convergence in the sense of Young measures is characterized by Theorem 2.1. We recall now a compactness criteria for Young measures.

#### Theorem 2.3

Let $\{\mu _n\}_{n\in \mathbb {N}}\subset \mathcal {Y}$ be such that

$$\begin{aligned} \forall \delta >0,\ x\in \mathbb {T}^d\ \exists A_x\subset \mathbb {R}^k \textit{ compact: } \ \sup _{n\in \mathbb {N}}\int _{\mathbb {T}^d}^* \mu _n(x,db)(A_x^c) dx<\delta , \end{aligned}$$

then there exists a subsequence $n_k$ and a Young measure μ such that

$$\begin{aligned} \mu _{n_k}{\mathop {\rightarrow }\limits ^{\mathcal {Y}}}\mu . \end{aligned}$$

Let us denote by ${U}_R(b_0)\subset \mathbb {R}^k$ the closed ball of radius R>0 centered at $b_0\in \mathbb {R}^k$. Combining Theorem 2.1, Theorem 2.3 and the Ascoli-Arzelà theorem for functions with values in metric spaces [26], we obtain the following result:

#### Lemma 2.4

Fix δ>0, introduce for each $i,k\in \mathbb {N}$ some positive quantities R=R(k,δ) and $\theta =\theta (k,\delta ,i)$, then the set

$$\begin{aligned} K^{\delta }:=\bigcap _{k\in \mathbb {N}}\Big \{\mu \in C([0,T];\mathcal {Y}):&\sup _{t\in [0,T]}\int _{\mathbb {T}^d}^* \mu _t(x,db)(U_R(0)^c)dx\le \frac{1}{k},\\  &\sup _{i\in \mathbb {N}}\sup _{|t-s|< \theta }\left\lvert \int _{\mathbb {T}^d\times \mathbb {R}^k} g_i(\textbf{x}) (\mu _t-\mu _s)(d\textbf{x})\right\rvert \le \frac{1}{k}\Big \} \end{aligned}$$

is relatively compact in $C([0,T];\mathcal {Y})$.

#### Proof

Given a sequence $(\mu ^n)\subset K^{\delta },$ by the Ascoli-Arzelà theorem, the claim is equivalent to the fact that

1. $\forall t\in [0,T]$ $\{\mu ^n_t\}$ is relatively compact in $\mathcal {Y}$.
2. Uniform equicontinuity holds.

The first condition is immediately true due to Theorem 2.3, concerning the second one, let us fix ε and find $\overline{k},\ \overline{i}\in \mathbb {N}$ such that

$$\begin{aligned} \overline{k}>{\frac{2}{\varepsilon }},\quad \sum _{i\ge \overline{i}}\frac{|\mathbb {T}^d|}{2^{i-1}}\le \frac{\varepsilon }{2} \end{aligned}$$

and $\gamma =\min \{\theta (\overline{k},\delta ,i),\quad i\in \{1,\dots ,\overline{i}\}\}>0$. Therefore, for each $n\in \mathbb {N},\ |t-s|\le \gamma$

$$\begin{aligned} \rho (\mu ^n_t,\mu ^n_s)&\le \sum _{i=1}^{\overline{i}-1} \frac{\left\lvert \int _{\mathbb {T}^d\times \mathbb {R}^k} g_i(\textbf{x}) (\mu ^n_t-\mu ^n_s)(d\textbf{x})\right\rvert }{2^i}+2|\mathbb {T}^d|\sum _{i\ge \overline{i}}\frac{1}{2^{i}}\\  &\le \frac{\varepsilon }{2}\sum _{i=1}^{\overline{i}-1} \frac{1}{2^i}+\frac{\varepsilon }{2}<\varepsilon . \end{aligned}$$

Hence, the claim follows from Theorem 2.1. □

### Main results

### Stochastic passive scalar

We begin by describing the coefficients of the stochastic transport term appearing in the transport equation in subsection 1.1. Let $(\Omega ,\mathcal {F},\mathcal {F}_t,\mathbb {P})$ be a complete, right-continuous, filtered probability space as described in subsection 2.1 and $\{W_t^{k,j}\},$ ${{k\in \mathbb {Z}^{d}_0, \ j\in \{1,\dots , d-1\}}}$ a sequence of complex-valued Brownian motions adapted to $\mathcal {F}_t$ such that $W^{-k,j}_t=(W^{k,j}_t)^*$ and

$$\begin{aligned} \mathbb {E} \left[ W^{k,j}_1,{W^{l,m}_1}^*\right]&={\left\{ \begin{array}{ll} 2 &  \textit{if } k=l, \ m=j\\ 0 &  \textit{otherwise;} \end{array}\right. } \quad \left[ W^{k,j}_\cdot , W^{l,m}_\cdot \right] _t={\left\{ \begin{array}{ll} 2t &  \textit{if } k=-l, \ m=j\\ 0 &  \textit{otherwise. } \end{array}\right. } \end{aligned}$$

Secondly, for each $n\in \mathbb {N},\ k\in \mathbb {Z}^{d}_0,\ j\in \{1,\dots , d-1\}$ we denote by $\sigma _{k,j}^{n}(x)=\theta _{k,j}^{n}a_{k,j}e^{ik\cdot x}$, where $\left\{ \frac{k}{|k|}, a_{k,1},\dots , a_{k,d-1}\right\}$ is an orthonormal system of $\mathbb {R}^d$ for $k\in \Gamma _{d,+}$ and $a_{k,j}=a_{-k,j}$ if $k\in \Gamma _{d,-}$, while

2.1 $$\begin{aligned} \theta _{k,j}^{n}&=\sqrt{\frac{d \kappa _T}{(d-1)c_n}}\boldsymbol{1}_{\{n\le |k|\le 2n\} }\frac{1}{|k|^{d/2}},\quad c_n=\sum _{\begin{array}{c} k\in \mathbb {Z}^d_0\\ n\le |k|\le 2n \end{array}}\frac{1}{|k|^{d}}=O(1),\quad \kappa _T>0 . \end{aligned}$$

With our choices, we ensure that

$$\begin{aligned} W^n_t:=\sum _{\begin{array}{c} k\in \mathbb {Z}^d_0\\ j\in \{1,\dots ,d-1\} \end{array}}\sigma _{k,j}^{n}W^{k,j}_t \end{aligned}$$

takes values in a space of real valued functions. Moreover, it is well known that the family $\{\frac{{a_{k,j}}}{2\pi \sqrt{2\pi }}e^{ik\cdot x}\}_{{k\in \mathbb {Z}^d_0, j\in \{1,\dots ,d-1\}}}$ is a complete orthogonal systems of *H* made by eigenfunctions of -Δ. Thanks to this choice of the coefficients, the equation for the passive scalar of subsection 1.1 can be rewritten in Itô form as

2.2 $$\begin{aligned} {\left\{ \begin{array}{ll} d\theta ^n_t& =\kappa _T\Delta \theta ^n_t dt+\sum _{\begin{array}{c} k\in \mathbb {Z}^d_0\\ j\in \{1,\dots ,d-1\} \end{array}}\sigma _{k,j}^{n}\cdot \nabla \theta ^n_t dW^{k,j}_t,\\ \theta ^n_0& =\overline{\theta }_0, \end{array}\right. } \end{aligned}$$

see [21, Section 2.3] for details. We are interested in analytically weak solutions of (2.2) as described by the following definition.

#### Definition 2.5

A stochastic process $\theta ^n\in C_{\mathcal {F}}([0,T];{L}^2(\mathbb {T}^d))\cap L^2_{\mathcal {F}}(0,T;{H}^1(\mathbb {T}^d))$ is a weak solution of equation (2.2) if $\mathbb {P}-$a.s.

$$\begin{aligned} \langle \theta ^n_t,\phi \rangle -\langle \overline{\theta }_0,\phi \rangle&=-\kappa _T\int _0^t \langle \nabla \theta ^n_s,\nabla \phi \rangle ds+\sum _{\begin{array}{c} k\in \mathbb {Z}^d_0\\ j\in \{1,\dots ,d-1\} \end{array}} \int _0^t \langle \sigma _{k,j}^{n}\cdot \nabla \theta ^n_s ,\phi \rangle dW^{k,j}_s \end{aligned}$$

for each $$\phi \in {H}^1(\mathbb {T}^d),\ t\in [0,T]$$.

The well-posedness of (2.2) in the sense of Definition 2.5 is guaranteed by the following standard result, which collects also some a priori estimates crucial for the following arguments.

#### Theorem 2.6

For each $\overline{\theta }_0\in {H}^1(\mathbb {T}^d)$ there exists a unique weak solution of (2.2) in the sense of Definition 2.5. Moreover $\theta ^n\in C_{\mathcal {F}}(0,T;{H}^1_w(\mathbb {T}^d))$ and

$$\begin{aligned} d\left\| \theta ^n_t\right\| ^2&=0\quad \mathbb {P}-a.s. \end{aligned}$$

Theorem 2.6 might be well known to the experts. Therefore, we omit its proof which however can be obtained following verbatim the one provided below in a more complex framework for the magnetic field, see Theorem 2.10 and Appendix A. As discussed in subsection 1.1, the results of [17, 21] imply, due to the choice of the coefficients (2.1), that the family $\theta ^n$ converges *weakly* to $\overline{\theta }$ solving

2.3 $$\begin{aligned} {\left\{ \begin{array}{ll} \partial _t \overline{\theta }(x,t)& =\kappa _T \Delta \overline{\theta }(t,x)\quad x\in \mathbb {T}^d,\ t\in [0,T]\\ \overline{\theta }(0,x)& =\overline{\theta }_0. \end{array}\right. } \end{aligned}$$

Our way to reinterpret this result in our framework is the following: let us introduce the family of Young measures

$$\begin{aligned} \mu _{n}(t,x,d\theta )=\delta _{\theta ^n_t(x)}(d\theta ), \end{aligned}$$

then the following holds:

#### Theorem 2.7

For every pair $\varphi \in C_b(\mathbb {R}),\psi \in C_b(\mathbb {T}^d)$ and for every $t\in [0,T],\ \overline{\theta }_0\in {H}^1$, denoting by

$$\begin{aligned} {\mu }(t,x,d\theta ):= \int _{\mathbb {T}^d}p(\kappa _T t,x-y)\delta _{\overline{\theta }_0(y)}(d\theta ) dy, \end{aligned}$$

then

2.4 $$\begin{aligned} \lim _{n\rightarrow +\infty }\mathbb {E} \left[ \left\lvert \int _{\mathbb {T}^d\times \mathbb {R}}\varphi (\theta )\psi (x)\mu _n(t,x,d\theta ) dx -\int _{\mathbb {T}^d\times \mathbb {R}}\varphi (\theta )\psi (x)\mu (t,x,d\theta ) dx \right\rvert ^2\right] =0. \end{aligned}$$

In particular μ satisfies

2.5 $$\begin{aligned} \begin{array}{c} \int _{\mathbb {T}^d\times \mathbb {R}}\varphi (\theta )\psi (x)\mu (t,x,d\theta ) dx-\int _{\mathbb {T}^d\times \mathbb {R}}\varphi (\theta )\psi (x)\delta _{\overline{\theta }_0(x)} dx\\ =\kappa _T\int _0^t\int _{\mathbb {T}^d\times \mathbb {R}}\varphi (\theta )\Delta \psi (x)\mu (s,x,d\theta ) dx ds \end{array}\end{aligned}$$

for each $\varphi \in C_b(\mathbb {R}),\ \psi \in C^2_b(\mathbb {T}^d)$ and

$$ \overline{\theta }\left( x,t\right) =\int _{\mathbb {R}}\theta {\mu }\left( t,x,d\theta \right) . $$

Unlike the PDE for $\overline{\theta }$, the limit PDE for μ retains the transport structure of the SPDE satisfied by $\theta ^n$. Indeed, choosing formally $\psi \equiv 1,\ \varphi (\theta )=|\theta |^2$ we obtain

$$\begin{aligned} \frac{d}{dt}\int _{\mathbb {T}^d\times \mathbb {R}}|\theta |^2\mu (t,x,d\theta )dx=0. \end{aligned}$$

By Itô formula in Theorem 2.6 we also have

$$\begin{aligned} \frac{d}{dt}\int _{\mathbb {T}^d\times \mathbb {R}}|\theta |^2\mu _n(t,x,d\theta )dx=0. \end{aligned}$$

The relation above fails obviously for the measure $\delta _{\overline{\theta }_t(x)}(d\theta )dx$. We regard this as a consequence of the fact that the measure-valued formulation (2.5) and the associated Young measure $\mu (t,x,d\theta )$ contain more information about their approximating sequence than (2.3) and its solution $\overline{\theta }$. Indeed, in the usual interpretation of Young measures, the limit leading to (2.3) can be understood as an averaging with respect to the Young measure that captures the underlying oscillations. Consequently, while one can trivially deduce (2.3) from (2.5) and recover $\overline{\theta }_t$ from $\mu _t$, the converse does not hold. We will come back on these issues in Remark 3.2.

### Stochastic passive vector field

Similarly to previous subsection, we start introducing the noise affecting our equation for the magnetic field. Let $\{W_t^{k,j}\},$ ${{k\in \mathbb {Z}^{3}_0, \ j\in \{1,2\}}}$ be a sequence of complex-valued Brownian motions adapted to $\mathcal {F}_t$ such that $W^{-k,j}_t=(W^{k,j}_t)^*$ and

$$\begin{aligned} \mathbb {E} \left[ W^{k,j}_1,{W^{l,m}_1}^*\right]&={\left\{ \begin{array}{ll} 2 &  \textit{if } k=l, \ m=j\\ 0 &  \textit{otherwise;} \end{array}\right. } \quad \left[ W^{k,j}_\cdot , W^{l,m}_\cdot \right] _t={\left\{ \begin{array}{ll} 2t &  \textit{if } k=-l, \ m=j\\ 0 &  \textit{otherwise. } \end{array}\right. } \end{aligned}$$

Secondly, for each $n\in \mathbb {N},\ k\in \mathbb {Z}^{3}_0,\ j\in \{1,2\}$ we denote by $\sigma _{k,j}^{n}(x)=\theta _{k,j}^{n}a_{k,j}e^{ik\cdot x}$, where $\left\{ \frac{k}{|k|}, a_{k,1}, a_{k,2}\right\}$ is an orthonormal system of $\mathbb {R}^3$ for $k\in \Gamma _{3,+}$ and $a_{k,j}=a_{-k,j}$ if $k\in \Gamma _{3,-}$, while

2.6 $$\begin{aligned} \theta _{k,j}^{n}&=\sqrt{\chi }\boldsymbol{1}_{\{n\le |k|\le 2n\} }\frac{1}{|k|^{5/2}},\ {\chi }>0. \end{aligned}$$

With our choices, we ensure that

$$\begin{aligned} W^n_t:=\sum _{\begin{array}{c} k\in \mathbb {Z}^d_0\\ j\in \{1,\dots ,d-1\} \end{array}}\sigma _{k,j}^{n}W^{k,j}_t \end{aligned}$$

takes values in a space of real valued functions and the family $\{a_{k,j}\frac{e^{ik\cdot x}}{2\pi \sqrt{2\pi }}\}_{{k\in \mathbb {Z}^{3}_0, j\in \{1,2\}}}$ is a complete orthogonal systems of *H* made by eigenfunctions of -Δ. We set

2.7 $$\begin{aligned} \eta _n=\sum _{\begin{array}{c} k\in \mathbb {Z}^3_0\\ n\le |k|\le 2n \end{array}}\frac{1}{|k|^5}=O(n^{-2}),\quad \alpha _n=\sum _{\begin{array}{c} k\in \mathbb {Z}^3_0\\ n\le |k|\le 2n \end{array}}\frac{1}{|k|^3}=4\pi \log 2+O(n^{-1}). \end{aligned}$$

Setting

$$\begin{aligned} W^n_t=\sum _{\begin{array}{c} k\in \mathbb {Z}^{3}_0\\ j\in \{1,2\} \end{array}}\sigma _{k,j}^{n}W^{k,j}_t, \end{aligned}$$

thanks to this choice of the coefficients, the equation for the magnetic field of subsection 1.2 can be rewritten in Itô form as

2.8 $$\begin{aligned} {\left\{ \begin{array}{ll} dB^n_t& =\frac{2}{3}\chi \eta _n\Delta B^n_t dt+\sum _{\begin{array}{c} k\in \mathbb {Z}^{3}_0\\ j\in \{1,2\} \end{array}}(\sigma _{k,j}^{n}\cdot \nabla B^n_t-B^n_t\cdot \nabla \sigma _{k,j}^{n})dW^{k,j}_t,\\ {{\,\textrm{div}\,}}B^n_t& =0,\\ B^n_0& =\overline{B}_0, \end{array}\right. } \end{aligned}$$

see [12, Section 2] for details.

#### Remark 2.8

It is important here to appreciate the different scaling imposed on the noise coefficients (2.6) with respect to [12], and other previous works on the same scaling limit, starting from [21]. In those works, the Itô–Stratonovich corrector produced a diffusive operator of order one, whereas here, due to our choices, this operator has size $\frac{2}{3}\chi \eta _n\Delta B^n_t$ and vanishes as $n \rightarrow \infty$. By contrast, another corrector, $\mathcal {L}^n(b)$, arises in the equation for the Young measure associated with $B_t^n$ (cf. equation (4.1) and Lemma 4.3). This corrector, arising solely from the stretching part of the noise, is of order $\alpha _n$ and therefore does not vanish in the limit.

We are interested to analytically weak solution of (2.8) as described by the following definition.

#### Definition 2.9

A stochastic process $B^n\in C_{\mathcal {F}}([0,T];H)\cap L^2_{\mathcal {F}}(0,T;V)$ is a weak solution of equation (2.8) if $\mathbb {P}-$a.s.

$$\begin{aligned} \langle B^n_t,\phi \rangle -\langle \overline{B}_0,\phi \rangle&=-\frac{2}{3}\chi \eta _n\int _0^t \langle \nabla B^n_s,\nabla \phi \rangle ds\\  &\quad \,\, +\sum _{\begin{array}{c} k\in \mathbb {Z}^{3}_0\\ j\in \{1,2\} \end{array}}\int _0^t \langle \sigma _{k,j}^n \cdot \nabla B^n_s - B^n_s\cdot \nabla \sigma _{k,j}^n ,\phi \rangle dW^{k,j}_s \end{aligned}$$

for each $$\phi \in V,\ t\in [0,T]$$.

The well-posedness of (2.8) in the sense of Definition 2.9 is guaranteed by the following standard result, which collect also some a priori estimates crucial for the following arguments.

#### Theorem 2.10

For each $\overline{B}_0\in V$ there exists a unique weak solution of (2.8) in the sense of Definition 2.9. Moreover $B^n\in C_{\mathcal {F}}(0,T;V_w)$ and the following Itô formulas hold

2.9 $$\begin{aligned} d\left\| B^n_t\right\| ^2&=-2\sum _{\begin{array}{c} k\in \mathbb {Z}^{3}_0\\ j\in \{1,2\} \end{array}}\langle B^n_t\cdot \nabla \sigma _{k,j}^{n}, B^n_t\rangle dW^{k,j}_t+2\sum _{\begin{array}{c} k\in \mathbb {Z}^{3}_0\\ j\in \{1,2\} \end{array}}\left\| B^n_t\cdot \nabla \sigma _{k,j}^{n}\right\| ^2 dt,\end{aligned}$$

2.10 $$\begin{aligned} d\left\| B^n_t\right\| ^\gamma&=-\gamma \left\| B^n_t\right\| ^{\gamma -2}\sum _{\begin{array}{c} k\in \mathbb {Z}^{3}_0\\ j\in \{1,2\} \end{array}}\langle B^n_t\cdot \nabla \sigma _{k,j}^{n}, B^n_t\rangle dW^{k,j}_t \nonumber \\  &+\gamma \left\| B^n_t\right\| ^{\gamma -2}\sum _{\begin{array}{c} k\in \mathbb {Z}^{3}_0\\ j\in \{1,2\} \end{array}}\left\| B^n_t\cdot \nabla \sigma _{k,j}^{n}\right\| ^2 dt\nonumber \\  &+ \frac{\gamma }{2}\frac{\gamma -2}{2}\left\| B^n_t\right\| ^{\gamma -4}\sum _{\begin{array}{c} k\in \mathbb {Z}^{3}_0\\ j\in \{1,2\} \end{array}}|\langle B^n_t\cdot \nabla \sigma _{k,j}^{n}, B^n_t\rangle |^2 dt \quad \text {for } \gamma \ge 4. \end{aligned}$$

In particular

2.11 $$\begin{aligned} \mathbb {E} \left[ \sup _{t\in [0,T]}\left\| B^n_t\right\| ^2\right]&\le C_0(\overline{B}_0,T,\chi ),\end{aligned}$$

2.12 $$\begin{aligned} \mathbb {E} \left[ \sup _{t\in [0,T]}\left\| B^n_t\right\| ^\gamma \right]&\le C_1(\overline{B}_0,T,\chi ,\gamma )\quad \text {for } \gamma \ge 4. \end{aligned}$$

Theorem 2.10 might be well known to the experts. Therefore, we postpone its proof which is based on standard arguments, namely compactness via a vanishing viscosity procedure, to Appendix A. According to the results of [12], due to the choice of the coefficients (2.7), the family $B^n$ converges *weakly* to the constant profile $\overline{B}_t\equiv \overline{B}_0$. However, looking at the family of Young measures

$$\begin{aligned} \mu _{n}(t,x,db)=\delta _{B^n_t(x)}(db), \end{aligned}$$

more can be seen. Indeed, denoting by

2.13 $$\begin{aligned} \mathcal {L}(b):=\frac{8\pi \chi \log 2}{15}\left( 2|b|^2 I-b\otimes b\right) \end{aligned}$$

and by μ the solution of the following PDE

$$\begin{aligned} {\left\{ \begin{array}{ll} \partial _t \mu (t,x,db)& ={{\,\textrm{div}\,}}(\mathcal {L}(b)\mu (t,x,db))\quad b\in \mathbb {R}^3,x\in \mathbb {T}^3,\ t\in [0,T]\\ \mu (0,x,db)& =\delta _{\overline{B}_0(x)}(db), \end{array}\right. } \end{aligned}$$

defined rigorously in section 4, then our main result reads as follows:

#### Theorem 2.11

For each $\overline{B}_0\in V$, $\mu _{n}$ converges in probability in $C([0,T];\mathcal {Y})$ to ${\mu }$, where μ is the unique weak solution of (4.23) in the sense of Definition 4.8. Moreover

$$\begin{aligned} \int _{\mathbb {R}^3}b\mu (t,x,db)= \overline{B}_0(x), \qquad \forall t\ge 0. \end{aligned}$$

An important consequence of the previous theorem is the possibility of deducing a dynamo-like property for $B^n_t$ which is not visible from the limit equation for $\overline{B}_t$, and is difficult to extract from the stochastic equation itself. In the context of Magnetohydrodynamics, the passive vector advection equation (2.8) models the evolution of a magnetic field induced by the motion of a (turbulent) ideal conducting fluid, like a plasma. A well-known phenomenon in plasma physics is the self-excitation of the magnetic field, resulting from the transfer of kinetic energy of the fluid into magnetic energy. Mathematically, this can be formulated as an asymptotic exponential growth of the $L^2$ norm of the solution of (2.5). For more details on the physics of the dynamo, we refer the interested reader to the monographs [13, 29]. In our setting we can see that the limit equation for μ allows to deduce a dynamo-like property of the solution. Indeed, choosing formally $f(b)=|b|^2$ as test function for the equation satisfied by μ (this can be done rigorously by approximation as in Proposition 4.7 below), we obtain, by simple computations,

$$\begin{aligned} \int _{\mathbb {T}^3\times \mathbb {R}^3} |b|^2\mu (t,x,db)dx&=\left\| \overline{B}_0\right\| ^2+\frac{16\pi \chi \log 2}{3}\int _0^t \int _{\mathbb {T}^3\times \mathbb {R}^3} |b|^2\mu (s,x,db)dx ds. \end{aligned}$$

Therefore

$$\begin{aligned} \int _{\mathbb {T}^3\times \mathbb {R}^3} |b|^2\mu (t,x,db)dx=e^{\frac{16\pi \chi \log 2}{3}t}\left\| \overline{B}_0\right\| ^2. \end{aligned}$$

We stress once again that this is due to the only weak convergence, indeed

$$\begin{aligned} \int _{\mathbb {T}^3\times \mathbb {R}^3} |b|^2\delta _{\overline{B}_t(x)}(db)dx=\left\| \overline{B}_0\right\| ^2. \end{aligned}$$

More importantly, the convergence of $\mu ^n$ towards μ allows to transfer dynamo-like properties to the magnetic field $B^n_t$, *before* taking the limit $n\rightarrow \infty$. A rigorous formulation of this observation, together with a discussion on the support properties of the measure μ(t,x,db) is provided in Appendix B.

## The scalar case

We start by observing that, by density, it is enough to prove the validity of equation Theorem 2.7 for $\varphi \in C^2_b(\mathbb {R}),\ \psi \in C^2_b(\mathbb {T}^d)$. Indeed, assume relation (2.4) holds for $\varphi \in C^2_b(\mathbb {R}),\ \psi \in C^2_b(\mathbb {T}^d)$. Let us now consider $\varphi \in C_b(\mathbb {R}),\ \psi \in C_b(\mathbb {T}^d)$. For each ε>0, by density it is possible to find $\varphi ^N\in C^2_b(\mathbb {R}),\ \psi ^N \in C^2_b(\mathbb {T}^d)$ such that

$$\begin{aligned} \left\| \varphi ^N-\varphi \right\| _{C_b}<\sqrt{\varepsilon },\quad \left\| \psi ^N-\psi \right\| _{C_b}<\sqrt{\varepsilon }. \end{aligned}$$

Therefore

$$\begin{aligned}&\mathbb {E} \left[ \left\lvert \int _{\mathbb {T}^d\times \mathbb {R}}\varphi (\theta )\psi (x)\mu _n(t,x,d\theta ) dx -\int _{\mathbb {T}^d\times \mathbb {R}}\varphi (\theta )\psi (x)\mu (t,x,d\theta ) dx \right\rvert ^2\right] \\  &\quad \lesssim \varepsilon +\mathbb {E} \left[ \left\lvert \int _{\mathbb {T}^d\times \mathbb {R}}\varphi ^N(\theta )\psi ^N(x)\mu _n(t,x,d\theta ) dx -\int _{\mathbb {T}^d\times \mathbb {R}}\varphi ^N(\theta )\psi ^N(x)\mu (t,x,d\theta ) dx \right\rvert ^2\right] . \end{aligned}$$

Considering the limsup in *n* of the expression above we obtain

$$\begin{aligned} \limsup _{n\rightarrow +\infty }\mathbb {E} \left[ \left\lvert \int _{\mathbb {T}^d\times \mathbb {R}}\varphi (\theta )\psi (x)\mu _n(t,x,d\theta ) dx -\int _{\mathbb {T}^d\times \mathbb {R}}\varphi (\theta )\psi (x)\mu (t,x,d\theta ) dx \right\rvert ^2\right] \lesssim \varepsilon \end{aligned}$$

and the claim follows from the arbitrariness of ε. Secondly, one can prove that the solutions given by Theorem 2.6 are renormalizable. By this we mean that, given $\varphi \in C^2_b(\mathbb {R}),\ \psi \in C^2_b(\mathbb {T}^d)$, one has

$$\begin{aligned} \varphi \left( \theta ^n_t \right) =\int _{\mathbb {R}}\varphi (\theta )d\delta _{\theta ^n_t(\cdot )}\in C_{\mathcal {F}}([0,T];H^1_w(\mathbb {T}^d))\cap C_{\mathcal {F}}([0,T];L^2(\mathbb {T}^d)) \end{aligned}$$

and

3.1 $$\begin{aligned} \int _{\mathbb {T}^d} \varphi (\theta ^n_t(x))\psi (x) dx&=\int _{\mathbb {T}^d} \varphi (\overline{\theta }_0(x))\psi (x) dx+\kappa _T\int _0^t\int _{\mathbb {T}^d} \varphi ({\theta }_s(x))\Delta \psi (x) dxds\nonumber \\  &\quad -\sum _{\begin{array}{c} k\in \mathbb {Z}^d_0\\ j\in \{1,\dots ,d\} \end{array}}\int _0^t\int _{\mathbb {T}^d} \varphi ({\theta }_s(x))\sigma _{k,j}^{n}\cdot \nabla \psi (x)dW^{k,j}_s\quad \mathbb {P}-a.s. \end{aligned}$$

In particular, by Theorem 2.6, it is the unique solution of (2.2) in the sense of Definition 2.5 with initial condition $\varphi \circ \overline{\theta }_0.$ The proof of this claim can be obtained following verbatim the argument provided below in subsection 4.1 in a more complex framework for the magnetic field, therefore we avoid to add details in this easier framework.

Recalling the definition of the Young measure

$$\begin{aligned} \mu _{n}\left( t,x,d\theta \right)&=\delta _{\theta ^n_t\left( x\right) }\left( d\theta \right) , \end{aligned}$$

we can rewrite (3.1) as

$$\begin{aligned} \int _{\mathbb {T}^d\times \mathbb {R}} \varphi (\theta )\psi (x)\mu _n(t,x,db)dx=&\int _{\mathbb {T}^d\times \mathbb {R}} \varphi (\theta )\psi (x)\delta _{\overline{\theta }_0(x)}(db)dx\\&+\kappa _T\int _0^t \int _{\mathbb {T}^d\times \mathbb {R}} \varphi (\theta )\Delta \psi (x)\mu _n(s,x,db)dxds\\&-\sum _{\begin{array}{c} k\in \mathbb {Z}_0^d\\ j\in \{1,\dots , d\} \end{array}}\int _0^t \int _{\mathbb {T}^d\times \mathbb {R}} \varphi (\theta )\sigma _{k,j}^{n}(x)\cdot \nabla \psi (x)\mu _n(s,x,db)dxds\ dW^{k,j}_s. \end{aligned}$$

Notice that we cannot deduce any convergence of $\int _{\mathbb {T}^d\times \mathbb {R}} \varphi (\theta )\psi (x)\mu _n(t,x,db)dx$ from the weak convergence of $\theta ^n$ guaranteed by the results of [17, 21]. Thus, the equations for $\theta ^n$ lead to equations for $\mu _{n}$, but convergence properties do not translate immediately from $\theta ^n$ to $\mu _{n}$.

The following remark may help to get an intuition about the main result of the section.

### Remark 3.1

Assume $\overline{\theta }_0\left( x\right) =\sum _{j}a_{j}\boldsymbol{1}_{D_{j}}\left( x\right)$ over a finite partition $\left( D_{j}\right)$, with different values $a_{j}$. Then

$$ \theta ^n\left( x,t\right) =\sum _{j}a_{j}\boldsymbol{1}_{D_{j}^{n}\left( t\right) }\left( x\right) $$

where $\left\{ D_{j}^{n}\left( t\right) \right\}$ is again a (random) partition, and

$$ \mu _{n}\left( t,x,d\theta \right) =\delta _{\theta ^n_t\left( x\right) }\left( d\theta \right) =\sum _{j}\delta _{a_{j}}\left( d\theta \right) \boldsymbol{1}_{D_{j}^{n}\left( t\right) }\left( x\right) . $$

The process $\boldsymbol{1}_{D_{j}^{n}\left( t\right) }\left( x\right)$ is the solution of the stochastic transport equation with initial condition $\boldsymbol{1}_{D_{j}}\left( x\right)$. Applying for every *j* the results above we get that $\mu _{n}\left( t,x,d\theta \right)$ weakly converges to

$$ {\mu }\left( t,x,d\theta \right) =\sum _{j}\delta _{a_{j}}\left( d\theta \right) \overline{\theta }_{j}\left( x,t\right) $$

where $\overline{\theta }_{j}\left( x,t\right)$ is the solution of

$$\begin{aligned} {\left\{ \begin{array}{ll} \partial _{t}\overline{\theta }_{j} &  =\kappa _{T}\Delta \overline{\theta }_{j}\\ \overline{\theta }_{j}|_{t=0} &  =\boldsymbol{1}_{D_{j}} \end{array}\right. } \end{aligned}$$

Now we are ready to provide the proof of Theorem 2.7.

### Proof of Theorem 2.7

According to the discussion above it is enough to show the theorem for $\varphi \in ~ C^2_b(\mathbb {R}),$ $\psi \in ~ C^2_b(\mathbb {T}^d).$ Moreover we know that, denoting by $\varphi ^n_t(x)=\varphi (\theta ^n_t(x))$, it solves

$$\begin{aligned} {\left\{ \begin{array}{ll} d\varphi ^n_t& =\kappa _T\Delta \varphi ^n_tdt+\sum _{\begin{array}{c} k\in \mathbb {Z}^d_0\\ j\in \{1,\dots ,d\} \end{array}}\sigma _{k,j}^{n}\cdot \nabla \varphi ^n_t dW^{k,j}_t\\ \varphi ^n_0& =\varphi \circ \overline{\theta }_0 \end{array}\right. } \end{aligned}$$

in the sense of Definition 2.5. Due to our choice of φ, $\sup _{t\in [0,T]}, \left\| \varphi ^n_t\right\| _{L^{\infty }}\le \left\| \varphi \right\| _{C_b}\ \mathbb {P}-a.s.$ Thanks to our choice of the coefficients $\sigma _{k,j}^{n}$, see in particular equation (2.1), we can apply [17, Theorem 1.7]. If we denote by $\overline{\varphi }_{\overline{\theta }_0}$ the unique weak solution of

$$\begin{aligned} {\left\{ \begin{array}{ll} \partial _t\overline{\varphi }_{\overline{\theta }_0}& =\kappa _T\Delta \overline{\varphi }_{\overline{\theta }_0}\\ \overline{\varphi }_{\overline{\theta }_0}(0)& =\varphi \circ \overline{\theta }_0, \end{array}\right. } \end{aligned}$$

for each ψ we have

$$\begin{aligned} \mathbb {E}\left[ \langle \overline{\varphi }_{\overline{\theta }_0}(t)-\varphi ^n(t),\psi \rangle ^2 \right] \lesssim \frac{\left\| \psi \right\| ^2\left\| \kappa _T\varphi \right\| _{C_b}^2}{n^d}\rightarrow 0\quad \text {as }n\rightarrow +\infty . \end{aligned}$$

Let us rewrite $\langle \overline{\varphi }_{\theta _0}(t),\psi \rangle$ and $\langle \varphi ^n(t),\psi \rangle$:

$$\begin{aligned} \langle \varphi ^n(t),\psi \rangle&=\int _{\mathbb {T}^d} \psi (x)\varphi \circ \theta ^n_t(x) dx\\  &= \int _{\mathbb {T}^d} \psi (x)\int _{\mathbb {R}}\varphi (\theta )\delta _{\theta ^n_t(x)}(d\theta ) dx\\  &= \int _{\mathbb {T}^d\times \mathbb {R}} \psi (x)\varphi (\theta )\mu _n(t,x,d\theta ) dx \end{aligned}$$

recalling the definition of $\mu _n(t,x,d\theta )$. While

$$\begin{aligned} \langle \overline{\varphi }_{\overline{\theta }_0},\psi \rangle&=\int _{\mathbb {T}^d} \psi (x)\int _{\mathbb {T}^d} p(\kappa _Tt,x-y)\varphi \circ \overline{\theta }_0(y) dy dx\\  &=\int _{\mathbb {T}^d} \psi (x)\int _{\mathbb {T}^d}\int _{\mathbb {R}} p(\kappa _Tt,x-y)\varphi (\theta ) \delta _{\overline{\theta }_0(y)}(d\theta ) dy dx\\  &=\int _{\mathbb {T}^d} \psi (x)\int _{\mathbb {R}}\varphi (\theta )\int _{\mathbb {T}^d}p(\kappa _Tt,x-y)\delta _{\overline{\theta }_0(y)}(d\theta ) dy dx\\  &= \int _{\mathbb {T}^d} \psi (x)\int _{\mathbb {R}}\varphi (\theta ){\mu }(t,x,d\theta ) dx, \end{aligned}$$

where

$$\begin{aligned} {\mu }(t,x,d\theta ):= \int _{\mathbb {T}^d}p(\kappa _Tt,x-y)\delta _{\overline{\theta }_0(y)}(d\theta ) dy. \end{aligned}$$

The remaining claims follow from the definition above of μ and the one of heat kernel. This concludes the proof. □

### Remark 3.2

The measure

$$ \overline{\mu }\left( t,x,d\theta \right) =\delta _{\overline{\theta }_t\left( x\right) }\left( d\theta \right) $$

is *not* a solution of the limit PDE. Indeed it satisfies

$$\begin{aligned} \int _{\mathbb {T}^d\times \mathbb {R}} \psi (x)\varphi (\theta )\overline{\mu }\left( t,x,d\theta \right) dx&=\int _{\mathbb {T}^d}\psi \left( x\right) \varphi \left( \overline{\theta }_t\left( x\right) \right) dx\\ \int _{\mathbb {T}^d\times \mathbb {R}} \psi (x)\varphi (\theta )\overline{\mu }\left( 0,x,d\theta \right) dx&=\int _{\mathbb {T}^d}\psi \left( x\right) \varphi \left( \overline{\theta }_0\left( x\right) \right) dx\\ \kappa _{T}\int _{0}^{t}\int _{\mathbb {T}^d\times \mathbb {R}}\Delta \psi (x)\varphi (\theta )\overline{\mu }\left( s,x,d\theta \right) dxds&=\kappa _{T}\int _{0}^{t}\int _{\mathbb {T}^d}\Delta \psi \left( x\right) \varphi \left( \overline{\theta }_s\left( x\right) \right) dxds \end{aligned}$$

and

$$\begin{aligned} \partial _{t}\varphi \left( \overline{\theta }_t\left( x\right) \right)&=\varphi ^{\prime }\left( \overline{\theta }_t\left( x\right) \right) \partial _{t}\overline{\theta }_t\left( x\right) \\ \nabla \varphi \left( \overline{\theta }_t\left( x\right) \right)&=\varphi ^{\prime }\left( \overline{\theta }_t\left( x\right) \right) \nabla \overline{\theta }_t\left( x\right) , \end{aligned}$$

but

$$\begin{aligned} \Delta \varphi \left( \overline{\theta }_t\left( t\right) \right)&=\varphi ^{\prime \prime }\left( \overline{\theta }_t\left( x\right) \right) \left| \nabla \overline{\theta }_t\left( x\right) \right| ^{2} +\varphi ^{\prime }\left( \overline{\theta }_t\left( x\right) \right) \Delta \overline{\theta }_t\left( x\right) \\&\ne \varphi ^{\prime }\left( \overline{\theta }_t\left( x\right) \right) \Delta \overline{\theta }_t\left( x\right) . \end{aligned}$$

Moreover, let us observe the following fact concerning the different behaviour of the limit measure $\mu (t,x,d\theta )$ and $\overline{\mu }(t,x,d\theta )$. Let us choose as test functions for relation (2.5) $\psi \equiv 1$ and a family $\varphi ^M:\mathbb {R}\rightarrow \mathbb {R}$ defined as $\varphi ^M(\theta )=M\wedge |\theta |^2$. In this way we obtain

$$\begin{aligned} \int _{\mathbb {T}^d\times \mathbb {R}}\varphi ^M(\theta )\mu (t,x,d\theta )dx=\int _{\mathbb {T}^d}M\wedge |\overline{\theta }_0(x)|^2 dx. \end{aligned}$$

Therefore, letting $M\rightarrow +\infty ,$ due to monotone convergence theorem

$$\begin{aligned} \int _{\mathbb {T}^d\times \mathbb {R}} |\theta |^2\mu (t,x,d\theta )dx=\left\| \overline{\theta }_0\right\| ^2. \end{aligned}$$

Similarly, the Itô formula of Theorem 2.6 gives

$$\begin{aligned} \int _{\mathbb {T}^d\times \mathbb {R}} |\theta |^2\mu _n(t,x,d\theta )dx=\left\| \overline{\theta }_0\right\| ^2. \end{aligned}$$

On the contrary, the dissipative nature of the equation satisfied by $\overline{\theta }$ implies

$$\begin{aligned} \int _{\mathbb {T}^d\times \mathbb {R}} |\theta |^2\overline{\mu }(t,x,d\theta )dx\le \left\| \overline{\theta }_0\right\| ^2e^{-2\kappa _T t}. \end{aligned}$$

### Remark 3.3

As the proof of Theorem 2.7 discussed above shows, [17, Theorem 1.7], namely the standard scaling limit of [21] made at the PDE level, and the renormalization property for $\theta ^n$ imply the convergence at the level of Young measures $\mu _n$. However, the convergence at the level of Young measures stated in Theorem 2.7 readily implies the convergence of $\theta ^n$ to $\overline{\theta }$ as well, at least in a weak sense. More precisely, let us assume that $\overline{\theta }_0\in L^{2}(\mathbb {T}^d)$ and consider a random Young measure $\mu \in C([0,T];\mathcal {Y})\ \mathbb {P}-a.s.$ satisfying (2.4) and (2.5). These conditions readily imply that

$$\begin{aligned} \sup _{t\in [0,T]}\mathbb {E}\left[ \int _{\mathbb {T}^d\times \mathbb {R}}|\theta |^2 \mu (t,x,d\theta )dx\right] \le \left\| \overline{\theta }_0\right\| ^2, \end{aligned}$$

and that $\overline{\theta }(t,x)=\int _{\mathbb {T}^d \times \mathbb {R}}\theta \mu (t,x,d\theta )$ is $\mathbb {P}-a.s.$ well-defined and satisfies (2.3). Consider now $\psi \in C_b(\mathbb {T}^3)$ and $\varphi ^M:\mathbb {R}\rightarrow \mathbb {R}$ defined as $\varphi ^M(\theta )=\theta \frac{M\wedge |\theta |}{|\theta |}$. For each M>1 and t∈[0,T] we have by Hölder’s and Markov’s inequalities

$$\begin{aligned}&\mathbb {E}\left[ \langle \theta ^n_t-\overline{\theta }_t, \psi \rangle ^2\right] \\&\quad \le 3 \mathbb {E}\left[ \left( \int _{\mathbb {T}^d\times \mathbb {R}} \varphi ^M(\theta )\psi (x)\mu _n(t,x,d\theta )dx-\int _{\mathbb {T}^d\times \mathbb {R}} \varphi ^M(\theta )\psi (x)\mu (t,x,d\theta )dx\right) ^2\right] \\&\qquad +3 \mathbb {E}\left[ \left( \int _{\mathbb {T}^d\times \mathbb {R}} |\theta |\boldsymbol{1}_{|\theta |>M}|\psi (x)|\mu (t,x,d\theta )dx\right) ^2\right] \\&\qquad +3\mathbb {E}\left[ \left( \int _{\mathbb {T}^d\times \mathbb {R}} |\theta |\boldsymbol{1}_{|\theta |>M}|\psi (x)|\mu _n(t,x,d\theta )dx\right) ^2\right] \\&\quad \le 3 \mathbb {E}\left[ \left( \int _{\mathbb {T}^d\times \mathbb {R}} \varphi ^M(\theta )\psi (x)\mu _n(t,x,d\theta )dx-\int _{\mathbb {T}^d\times \mathbb {R}} \varphi ^M(\theta )\psi (x)\mu (t,x,d\theta )dx\right) ^2\right] \\&\qquad +3\left\| \psi \right\| _{L^{\infty }(\mathbb {T}^d)}^2 \mathbb {E}\left[ \int _{\mathbb {T}^d\times \mathbb {R}} \boldsymbol{1}_{|\theta |>M}\mu (t,x,d\theta )dx\int _{\mathbb {T}^d\times \mathbb {R}} |\theta |^2\mu (t,x,d\theta )dx\right] \\&\qquad +3\left\| \psi \right\| _{L^{\infty }(\mathbb {T}^d)}^2 \mathbb {E}\left[ \int _{\mathbb {T}^d\times \mathbb {R}} \boldsymbol{1}_{|\theta |>M}\mu _n(t,x,d\theta )dx\int _{\mathbb {T}^d\times \mathbb {R}} |\theta |^2\mu _n(t,x,d\theta )dx\right] \\&\quad \le 3 \mathbb {E}\left[ \left( \int _{\mathbb {T}^d\times \mathbb {R}} \varphi ^M(\theta )\psi (x)\mu _n(t,x,d\theta )dx-\int _{\mathbb {T}^d\times \mathbb {R}} \varphi ^M(\theta )\psi (x)\mu (t,x,d\theta )dx\right) ^2\right] \\&\qquad +\frac{3|\mathbb {T}^d|}{M}\left\| \psi \right\| _{L^{\infty }(\mathbb {T}^d)}^2 \left( \mathbb {E}\left[ \int _{\mathbb {T}^d\times \mathbb {R}}|\theta |^2 \mu (t,x,d\theta )dx\right] +\mathbb {E}\left[ \int _{\mathbb {T}^d\times \mathbb {R}}|\theta |^2 \mu _n(t,x,d\theta )dx\right] \right) . \end{aligned}$$

Since $\mathbb {E}\left[ \int _{\mathbb {T}^d\times \mathbb {R}}|\theta |^2 \mu (t,x,d\theta )dx\right] +\sup _{n\in \mathbb {N}}\mathbb {E}\left[ \int _{\mathbb {T}^d\times \mathbb {R}}|\theta |^2 \mu _n(t,x,d\theta )dx\right] <+\infty$, we obtain

$$\begin{aligned} \limsup _{n\rightarrow +\infty }\mathbb {E}\left[ \langle \theta ^n_t-\overline{\theta }_t, \psi \rangle ^2\right]&\lesssim \frac{1}{M} \end{aligned}$$

and the claim follows from the arbitrariness of *M*.

## The case of the magnetic field

The proof of Theorem 2.11 relies on a stochastic compactness argument on $\mu _n(t,x,db)$. The proof is splitted in three main steps corresponding to subsection 4.1, subsection 4.2 and subsection 4.3. We prove some properties of the Young measure $\mu _n(t,x,db)$ in subsection 4.1. Such properties allow us to show the tightness of the laws of $\mu _n(t,x,db)$ in the space $C([0,T];\mathcal {Y})$ in subsection 4.2. Thanks to Skorokhod’s representation theorem, we can pass to an auxiliary probability space where the convergence in law becomes $\mathbb {P}-a.s.$ convergence in subsection 4.3. This allows us to identify a limit Young measure which satisfies a deterministic PDE and conclude the proof by uniqueness of the solutions of this PDE in our class.

In order to ease the notation, let us fix χ=1 in the following.

### Properties of the PDE for the magnetic field

In this section we collect some useful properties of the Young measure associated to $B^n$ solution of (2.8): we will introduce the Vlasov type SPDE satisfied by $\mu _n(t,x,db)=\delta _{B^n_t(x)}(db)$ and then we will prove some estimates on $\mu _n(t,x,db)$, uniformly in the scaling parameter *n*.

### Vlasov type SPDE for $\mu _n$

In this section we show that the measure $\mu _n(t,x,db)=\delta _{B^n_t(x)}(db)$ satisfied the Vlasov type PDE

4.1 $$\begin{aligned} {\left\{ \begin{array}{ll} &  d\mu _n = \sum _{\begin{array}{c} k\in \mathbb {Z}^{3}_0\\ j\in \{1,2\} \end{array}}{{\,\textrm{div}\,}}_b(b\cdot \nabla \sigma _{k,j}^{n}\mu _n) dW^{k,j}_t+\sum _{\begin{array}{c} k\in \mathbb {Z}^{3}_0\\ j\in \{1,2\} \end{array}}{{\,\textrm{div}\,}}_x(\sigma _{k,j}^{n}\mu _n) dW^{k,j}_t\\   & \qquad \qquad +{{\,\textrm{div}\,}}_b(\mathcal {L}^n(b)\nabla _b\mu _n)dt +\frac{2}{3}\eta _n\Delta _x \mu _{n}dt,\\ &  \mu _n(0,x,db)=\delta _{\overline{B}_0(x)}(db), \end{array}\right. } \end{aligned}$$

where

$$\begin{aligned} \mathcal {L}^n(b)=\sum _{\begin{array}{c} k\in \mathbb {Z}^3_0\\ n\le |k|\le 2n \end{array}}\frac{(b\cdot k)^2}{|k|^5}\left( I-\frac{k\otimes k}{|k|^2}\right) , \end{aligned}$$

in the sense that for each $f\in C^{2}_b(\mathbb {T}^3\times \mathbb {R}^3)$

4.2 $$\begin{aligned} \begin{array}{c} \int _{\mathbb {T}^3\times \mathbb {R}^3}f(x,b)\mu _n(t,x,db) dx-\int _{\mathbb {T}^3\times \mathbb {R}^3}f(x,b)\delta _{\overline{B}_0(x)}(db)dx\\ =-\sum _{\begin{array}{c} k\in \mathbb {Z}^{3}_0\\ j\in \{1,2\} \end{array}}\int _0^t\int _{\mathbb {T}^3\times \mathbb {R}^3} \left( \sigma _{k,j}^{n}(x)\cdot (\nabla _x f)(x,b)+b\cdot \nabla \sigma _{k,j}^{n}(x)\nabla _b f(x,b)\right) \mu _n(s,x,db)dxdW^{k,j}_s \\ +\frac{2}{3}\eta _n\int _0^t\int _{\mathbb {T}^3\times \mathbb {R}^3} \Delta _x f(x,b)\mu _n(s,x,db) dx ds +\int _0^t\int _{\mathbb {T}^3\times \mathbb {R}^3} {{\,\textrm{div}\,}}_b\left( \mathcal {L}^n(b)\nabla _b f(x,b)\right) \mu _n(s,x,db) dx ds \end{array}\end{aligned}$$

$$\mathbb {P}-a.s.$$ for each t∈[0,T].

#### Remark 4.1

Due to the definition of $\mu _n(t,x,db)$, relation (4.2) can be rewritten as

$$\begin{aligned}&\int _{\mathbb {T}^3}f(x,B^n_t(x))-f(x,\overline{B}_0(x)) dx\\&\quad =-\sum _{\begin{array}{c} k\in \mathbb {Z}^{3}_0\\ j\in \{1,2\} \end{array}}\int _0^t\int _{\mathbb {T}^3} \sigma _{k,j}^{n}(x)\cdot \left( \nabla _x f\right) (x,B^n_s(x))dW^{k,j}_s\\&\qquad -\sum _{\begin{array}{c} k\in \mathbb {Z}^{3}_0\\ j\in \{1,2\} \end{array}}\int _0^t\int _{\mathbb {T}^3}B^n_s(x)\cdot \nabla \sigma _{k,j}^{n}(x)\nabla _b f(x,B^n_s(x))dW^{k,j}_s \nonumber \\&\qquad +\frac{2}{3}\eta _n\int _0^t\int _{\mathbb {T}^3} \left( \Delta _x f\right) (x,B^n_s(x)) dxds\\&\qquad +\int _0^t\int _{\mathbb {T}^3} {{\,\textrm{div}\,}}_b\left( \mathcal {L}^n(B^n_s(x))\nabla _b f(x,B^n_s(x))\right) dx ds\quad \mathbb {P}-a.s. \ \forall t\in [0,T]. \end{aligned}$$

We will actually prove this relation, obtaining (4.2) thanks to the definition of $\mu _n$.

Let $\varrho \in C^{\infty }(\mathbb {R}^3)$ a non negative, even function such that

$$\begin{aligned} \int _{\mathbb {R}^3}\varrho (x)dx=1,\quad \operatorname {supp}\varrho \subset [-1/2,1/2]^3. \end{aligned}$$

For ε>0, let $\varrho _{\varepsilon }(\cdot )=\frac{1}{\varepsilon ^3}\varrho (\cdot /\varepsilon ).$ If $f\in L^1(\mathbb {T}^3)$ it can be extended to a locally integrable periodic function on $\mathbb {R}^3$, so that the convolution $\varrho _{\varepsilon }*f$ makes sense and it is still a periodic function. It is easy to show, see for example [12, Remark 2.15] that if $\phi \in C^{\infty }(\mathbb {T}^3)$ then

$$\begin{aligned} \langle B^n_t,\phi \rangle&=\langle B^n_0,\phi \rangle +\frac{2}{3}\eta _n\int _0^t \langle B^n_s,\Delta \phi \rangle ds-\sum _{\begin{array}{c} k\in \mathbb {Z}^{3}_0\\ j\in \{1,2\} \end{array}}\int _0^t \langle \sigma _{k,j}^{n}\cdot \nabla \phi ,B^n_s\rangle dW^{k,j}_s\\  &\quad -\sum _{\begin{array}{c} k\in \mathbb {Z}^{3}_0\\ j\in \{1,2\} \end{array}}\int _0^t \langle B^n_s\cdot \nabla \sigma _{k,j}^{n},\phi \rangle dW^{k,j}_s. \end{aligned}$$

In particular, denoting by $B^{n,\varepsilon }=B^n*\varrho _{\varepsilon }$ and choosing $\phi ^x(y)=\left( \varrho _{\varepsilon }(x-y),\varrho _{\varepsilon }(x-y),\right. \left. \varrho _{\varepsilon }(x-y)\right) ^t$ as a test function we obtain

4.3 $$\begin{aligned} B^{n,\varepsilon }_t(x)&=B^{n,\varepsilon }_0(x)+\frac{2}{3}\eta _n \int _0^t\Delta B^{n,\varepsilon }_s(x) ds\nonumber \\  &\quad +\sum _{\begin{array}{c} k\in \mathbb {Z}^{3}_0\\ j\in \{1,2\} \end{array}}\int _0^t\left( \sigma _{k,j}^{n}(x)\cdot \nabla B^{n,\varepsilon }_s(x)-B^{n,\varepsilon }_s(x)\cdot \nabla \sigma _{k,j}^{n}(x)\right) dW^{k,j}_s\nonumber \\  &\quad -\sum _{\begin{array}{c} k\in \mathbb {Z}^{3}_0\\ j\in \{1,2\} \end{array}}\int _0^t\int _{\mathbb {R}^3} \left( \sigma _{k,j}^{n}(x)-\sigma _{k,j}^{n}(x-z)\right) \cdot \nabla \varrho _{\varepsilon }(z)B^n_s(x-z) dz dW^{k,j}_s\nonumber \\  &\quad -\sum _{\begin{array}{c} k\in \mathbb {Z}^{3}_0\\ j\in \{1,2\} \end{array}}\int _0^t\int _{\mathbb {R}^3} B^n_s(z)\cdot \nabla \left( \sigma _{k,j}^{n}(z)-\sigma _{k,j}^{n}(x)\right) \varrho _{\varepsilon }(x-z) dz dW^{k,j}_s. \end{aligned}$$

From now on, in this subsection we will drop the index *n* in our notation, since we are interested in proving the validity of (4.2) for *n* fixed. In order to reach our goal, as it is customary in the framework of transport equations, we rely on a commutator lemma. Therefore, let us introduce a notation we will use in the following. If $v\in C^{\infty }(\mathbb {T}^3;\mathbb {R}^3),\ B\in L^p(\mathbb {T}^3;\mathbb {R}^3)$ are divergence free vector field for some $p\in [1,+\infty )$ we denote by

$$\begin{aligned} [v\cdot \nabla ,\varrho _{\varepsilon }]B:=\int _{\mathbb {R}^3} \left( v(x)-v(x-z)\right) \cdot \nabla \varrho _{\varepsilon }(z)B(x-z) dz \end{aligned}$$

and by

$$\begin{aligned} [\cdot \nabla v,\varrho _{\varepsilon }]B:=\int _{\mathbb {R}^3} B(z)\cdot \nabla \left( v(z)-v(x)\right) \varrho _{\varepsilon }(x-z) dz. \end{aligned}$$

#### Lemma 4.2

Let $p\in [1,+\infty )$, $v\in C^{\infty }(\mathbb {T}^3;\mathbb {R}^3),\ B\in L^p(\mathbb {T}^3;\mathbb {R}^3)$ divergence free vector fields. Then

$$\begin{aligned} \left\| [v\cdot \nabla ,\varrho _{\varepsilon }]B\right\| _{L^p(\mathbb {T}^3;\mathbb {R}^3)}\rightarrow 0,\quad \left\| [\cdot \nabla v,\varrho _{\varepsilon }]B\right\| _{L^p(\mathbb {T}^3;\mathbb {R}^3)}\rightarrow 0\quad \text {as }\varepsilon \rightarrow 0. \end{aligned}$$

Moreover

$$\begin{aligned}&\left\| [v\cdot \nabla ,\varrho _{\varepsilon }]B\right\| _{L^p(\mathbb {T}^3;\mathbb {R}^3)}\lesssim \left\| v\right\| _{C^1_b}\left\| \varrho \right\| _{C^1_b} \left\| B\right\| _{L^p},\\  &\quad \left\| [\cdot \nabla v,\varrho _{\varepsilon }]B\right\| _{L^p(\mathbb {T}^3;\mathbb {R}^3)}\lesssim \varepsilon \left\| v\right\| _{C^2_b}\left\| \varrho \right\| _{C^0_b}\left\| B\right\| _{L^p}. \end{aligned}$$

#### Proof

First we observe that by change of variables

$$\begin{aligned} [v\cdot \nabla ,\varrho _{\varepsilon }]B(x)&=\int _{\mathbb {R}^3}\frac{v(x)-v(x-\varepsilon y)}{\varepsilon }\nabla \varrho (y)B(x-\varepsilon y) dy. \end{aligned}$$

Secondly, since *v* is smooth, the fundamental theorem of calculus implies

$$\begin{aligned} v(x)-v(x-\varepsilon y)=\varepsilon \int _0^1 y\cdot \nabla v(x-(1-s)y) ds. \end{aligned}$$

Therefore

4.4 $$\begin{aligned} [v\cdot \nabla ,\varrho _{\varepsilon }]B(x)=\int _{\mathbb {R}^3}\int _0^1 y\cdot \nabla v(x-(1-s)\varepsilon y)\nabla \varrho (y) B(x-\varepsilon y)ds dy. \end{aligned}$$

Now we are ready to compute the $L^p$ norm of the commutator. Indeed, by Hölder’s inequality, since the support of ϱ is compact

$$\begin{aligned} \int _{\mathbb {T}^3}\left\lvert [v\cdot \nabla ,\varrho _{\varepsilon }]B(x)\right\rvert ^p dx&\lesssim \left\| v\right\| _{C^1_b}^p\left\| \varrho \right\| _{C^1_b}^p\int _{\mathbb {T}^3}\int _{\operatorname {supp} \varrho }|B(x-\varepsilon y)|^p dx dy\\  &\lesssim \left\| v\right\| _{C^1_b}^p\left\| \varrho \right\| _{C^1_b}^p \left\| B\right\| _{L^p}^p. \end{aligned}$$

Concerning the convergence to 0 we observe that, since

$$\begin{aligned} B(x)\int _{\mathbb {R}^3} y\cdot \nabla v(x)\nabla \varrho (y) dy&=-\sum _{i,j=1}^3 B(x)\int _{\mathbb {R}^3} \delta _{i,j} \partial _j v^j(x)\varrho (y) dy\\  &\equiv 0, \end{aligned}$$

it is enough to show that

$$\begin{aligned} \left\| [v\cdot \nabla ,\varrho _{\varepsilon }]B-B(\cdot )\int _{\mathbb {R}^3} y\cdot \nabla v(\cdot )\nabla \varrho (y) dy\right\| _{L^p}\rightarrow 0. \end{aligned}$$

Thanks to Hölder’s inequality, relation (4.4) and the fact that the support of ϱ is included in $[-1/2,1/2]^3$ we obtain

$$\begin{aligned}  &   \Big \Vert [v\cdot \nabla ,\varrho _{\varepsilon }]B-B(\cdot )\int _{\mathbb {R}^3} y\cdot \nabla v(\cdot )\nabla \varrho (y) dy\Big \Vert _{L^p}^p\\    &   \quad \lesssim \int _{[-1/2,1/2]^3}\int _0^1 \int _{\mathbb {T}^3} |B(x)\nabla v(x)-B(x-\varepsilon y)\nabla v(x-(1-s)\varepsilon y) |^p dx ds dy\\    &   \quad = \int _{[-1/2,1/2]^3} f^{\varepsilon }(y) dy, \end{aligned}$$

where we denoted by

$$\begin{aligned} f^{\varepsilon }(y)=\int _0^1 \int _{\mathbb {T}^3} |B(x)\nabla v(x)-B(x-\varepsilon y)\nabla v(x-(1-s)\varepsilon y) |^p dx ds. \end{aligned}$$

The function $f^{\varepsilon }\rightarrow 0$ a.e. indeed

$$\begin{aligned}&|B(x)\nabla v(x)-B(x-\varepsilon y)\nabla v(x-(1-s)\varepsilon y) |^p\\&\qquad \lesssim |B(x)\nabla v(x)-B(x-\varepsilon y)\nabla v(x-\varepsilon y) |^p \\&\qquad + |B(x-\varepsilon y)|^p|\nabla v(x-\varepsilon y) -\nabla v(x-(1-s)\varepsilon y) |^p\\&\quad \quad \le |B(x)\nabla v(x)-B(x-\varepsilon y)\nabla v(x-\varepsilon y) |^p+ \varepsilon ^p |B(x-\varepsilon y)|^p\left\| v\right\| _{C^2_b}^p. \end{aligned}$$

Therefore

$$\begin{aligned} f^{\varepsilon }(y)\lesssim \int _{0}^1 \int _{\mathbb {T}^3}|B(x)\nabla v(x)-B(x-\varepsilon y)\nabla v(x-\varepsilon y) |^p dx ds+\varepsilon ^p\left\| v\right\| _{C^2_b}^p \left\| B\right\| _{L^p}^p\rightarrow 0 \end{aligned}$$

since the first term converges to 0 thanks to the continuity of translations in $L^p$. Moreover, we can apply dominated convergence theorem since previous computations imply by change of variables

$$\begin{aligned} f^{\varepsilon }(y)\lesssim \left\| v\right\| _{C^2_b}^p \left\| B\right\| _{L^p}^p. \end{aligned}$$

The analysis of the second commutator is easier. Indeed, by change of variables

$$\begin{aligned} \left\lvert \int _{\mathbb {R}^3} B(z)\cdot \nabla \left( v(z)-v(x)\right) \varrho _{\varepsilon }(x-z) dz\right\rvert&=\left\lvert \int _{\mathbb {R}^3} B(x-\varepsilon z)\cdot \nabla \left( v(x-\varepsilon z)-v(x)\right) \varrho (z) dz\right\rvert \\  &\le \varepsilon \left\| v\right\| _{C^2_b}\left\| \varrho \right\| _{C^0_b}\int _{[-1/2,1/2]^3}|B(x-\varepsilon z)|dz. \end{aligned}$$

Therefore by Hölder’s inequality

$$\begin{aligned} \left\| [\cdot \nabla v,\varrho _{\varepsilon }]B\right\| _{L^p(\mathbb {T}^3;\mathbb {R}^3)}^p&\lesssim \varepsilon ^p\left\| v\right\| ^p_{C^2_b}\left\| \varrho \right\| ^p_{C^0_b}\left\| B\right\| _{L^p}^p\rightarrow 0. \end{aligned}$$

□

Next step is to compute, for a smooth function $f\in C^{2}_b(\mathbb {T}^3\times \mathbb {R}^3)$ the evolution of $\int _{\mathbb {T}^3} f(x,B^{\varepsilon }_t)dx.$ This is the content of the following lemma.

#### Lemma 4.3

For each $f\in C^{2}_b(\mathbb {T}^3\times \mathbb {R}^3)$ it holds

$$\begin{aligned}&\int _{\mathbb {T}^3} f(x,B^{\varepsilon }_t(x))dx= \int _{\mathbb {T}^3} f(x,B^{\varepsilon }_0(x))dx+\frac{2}{3}\eta \int _0^t\int _{\mathbb {T}^3} \left( \Delta _x f\right) (x,B^\varepsilon _s(x)) dxds\\&\quad +\int _0^t\int _{\mathbb {T}^3} {{\,\textrm{div}\,}}_b\left( \mathcal {L}(B^{\varepsilon }_s(x))\nabla _b f(x,B^\varepsilon _s(x))\right) dx ds \\  &\quad -\sum _{\begin{array}{c} k\in \mathbb {Z}^{3}_0\\ j\in \{1,2\} \end{array}}\int _0^t\int _{\mathbb {T}^3}B^\varepsilon _s(x)\cdot \nabla \sigma _{k,j}(x)\nabla _b f(x,B^\varepsilon _s(x))dx dW^{k,j}_s \\  &\quad - \sum _{\begin{array}{c} k\in \mathbb {Z}^{3}_0\\ j\in \{1,2\} \end{array}}\int _0^t\int _{\mathbb {T}^3}\sigma _{k,j}(x)\cdot (\nabla _x f)(x,B^\varepsilon _s(x))dx dW^{k,j}_s\\  &\quad -\sum _{\begin{array}{c} k\in \mathbb {Z}^{3}_0\\ j\in \{1,2\} \end{array}}\int _0^t \int _{\mathbb {T}^3}\left( [\sigma _{k,j}\cdot \nabla , \varrho _{\varepsilon }]+[\cdot \nabla \sigma _{k,j}, \varrho _{\varepsilon } ]\right) B_s(x) \cdot \nabla _b f(x,B^{\varepsilon }_s) dx dW^{k,j}_s \\  &\quad +\sum _{\begin{array}{c} k\in \mathbb {Z}^{3}_0\\ j\in \{1,2\} \end{array}}\int _0^t\int _{\mathbb {T}^3}Tr\left( \nabla ^2_b f(x,B^{\varepsilon }_s(x))\left( \left( [\sigma _{k,j}\cdot \nabla , \varrho _{\varepsilon }]+[\cdot \nabla \sigma _{k,j}, \varrho _{\varepsilon } ]\right) B_s(x) \otimes \left( [\sigma _{-k,j}\cdot \nabla , \varrho _{\varepsilon }]\right. \right. \right. \\  &\quad \quad \quad \quad \quad \quad \left. \left. \left. +[\cdot \nabla \sigma _{-k,j}, \varrho _{\varepsilon } ]\right) B_s(x)\right) \right) dx ds\\  &\quad -\sum _{\begin{array}{c} k\in \mathbb {Z}^{3}_0\\ j\in \{1,2\} \end{array}}\int _0^t\int _{\mathbb {T}^3}Tr\left( \nabla ^2_b f(x,B^{\varepsilon }_s(x))\left( \left( [\sigma _{k,j}\cdot \nabla , \varrho _{\varepsilon }]+[\cdot \nabla \sigma _{k,j}, \varrho _{\varepsilon } ]\right) B_s(x)\right. \right. \\  &\quad \quad \quad \quad \quad \quad \otimes \left. \left. \left( \sigma _{-k,j}(x)\cdot \nabla B^{\varepsilon }_s(x)-B^{\varepsilon }_s(x)\cdot \nabla \sigma _{-k,j}(x)\right) \right) \right) dx ds\\  &\quad -\sum _{\begin{array}{c} k\in \mathbb {Z}^{3}_0\\ j\in \{1,2\} \end{array}}\int _0^t\int _{\mathbb {T}^3}Tr\left( \nabla ^2_b f(x,B^{\varepsilon }_s(x))\left( \left( \sigma _{k,j}(x)\cdot \nabla B^{\varepsilon }_s(x)-B^{\varepsilon }_s(x)\cdot \nabla \sigma _{k,j}(x)\right) \right. \right. \\  &\quad \quad \quad \quad \quad \otimes \left. \left. \left( [\sigma _{-k,j}\cdot \nabla , \varrho _{\varepsilon }]+[\cdot \nabla \sigma _{-k,j}, \varrho _{\varepsilon } ]\right) B_s(x)\right) \right) dx ds. \end{aligned}$$

#### Proof

Since $B^{\varepsilon }$ is a smooth object in space which satisfies in a strong sense relation (4.3), we can apply finite dimensional Itô formula obtaining

4.5 $$\begin{aligned} f(x,B^{\varepsilon }_t(x))&=f(x,B^{\varepsilon }_0(x))+\frac{2}{3}\eta \int _0^t \nabla _b f(x,B^{\varepsilon }_s(x))\cdot \Delta B^{\varepsilon }_s(x)ds+\sum _{\begin{array}{c} k\in \mathbb {Z}^{3}_0\\ j\in \{1,2\} \end{array}}\int _0^t R^{k,j,\varepsilon ,f}_s(x) ds\nonumber \\  &\quad +\sum _{\begin{array}{c} k\in \mathbb {Z}^{3}_0\\ j\in \{1,2\} \end{array}}\int _0^t \nabla _b f(x,B^{\varepsilon }_s(x)) \cdot \left( \sigma _{k,j}(x)\cdot \nabla B^{\varepsilon }_s(x)-B^{\varepsilon }_s(x)\cdot \nabla \sigma _{k,j}(x)\right) dW^{k,j}\nonumber \\  &\quad -\sum _{\begin{array}{c} k\in \mathbb {Z}^{3}_0\\ j\in \{1,2\} \end{array}}\int _0^t \nabla _b f(x,B^{\varepsilon }_s(x)) \cdot \left( [\sigma _{k,j}\cdot \nabla ,\varrho _{\varepsilon }]+[\cdot \nabla \sigma _{k,j},\varrho _{\varepsilon }]\right) B_s(x)dW^{k,j}, \end{aligned}$$

where

$$\begin{aligned}&R^{k,j,\varepsilon ,f}_s(\cdot )\\  &=Tr(\nabla ^2_b f(\cdot ,B^{\varepsilon }_s(\cdot ))\left( \left( \sigma _{k,j}(\cdot )\cdot \nabla B^{\varepsilon }_s(\cdot )-B^{\varepsilon }_s(\cdot )\cdot \nabla \sigma _{k,j}(\cdot )-\left( [\sigma _{k,j}\cdot \nabla ,\varrho _{\varepsilon }]+[\cdot \nabla \sigma _{k,j},\varrho _{\varepsilon }]\right) B_s(\cdot )\right) \right. \\&\quad \left. \otimes \left( \sigma _{-k,j}(\cdot )\cdot \nabla B^{\varepsilon }_s(\cdot )-B^{\varepsilon }_s(\cdot )\cdot \nabla \sigma _{-k,j}(\cdot )-\left( [\sigma _{-k,j}\cdot \nabla ,\varrho _{\varepsilon }]+[\cdot \nabla \sigma _{-k,j},\varrho _{\varepsilon }]\right) B_s(\cdot ) \right) \right) . \end{aligned}$$

In order to get the claim integrating (4.5) on $\mathbb {T}^3$ we need to make some observations. First let us observe that by chain rule and integration by parts

$$\begin{aligned}&\int _{\mathbb {T}^3} \nabla _b f(x,B^{\varepsilon }_s(x))\cdot \left( \sigma _{k,j}(x)\cdot \nabla B^{\varepsilon }_s(x)\right) dx\\  &=\int _{\mathbb {T}^3} \nabla _x f(x,B^{\varepsilon }_s(x))\cdot \sigma _{k,j}(x)- (\nabla _x f)(x,B^{\varepsilon }_s(x))\cdot \sigma _{k,j}(x)dx\\  &=-\int _{\mathbb {T}^3} (\nabla _x f)(x,B^{\varepsilon }_s(x))\cdot \sigma _{k,j}(x)dx. \end{aligned}$$

Therefore

4.6 $$\begin{aligned}  &   \sum _{\begin{array}{c} k\in \mathbb {Z}^{3}_0\\ j\in \{1,2\} \end{array}}\int _0^t \int _{\mathbb {T}^3} \nabla _b f(x,B^{\varepsilon }_s(x)) \cdot \sigma _{k,j}(x)\cdot \nabla B^{\varepsilon }_s(x)dxdW^{k,j}\nonumber \\    &   \quad =- \sum _{\begin{array}{c} k\in \mathbb {Z}^{3}_0\\ j\in \{1,2\} \end{array}}\int _0^t \int _{\mathbb {T}^3} (\nabla _x f)(x,B^{\varepsilon }_s(x))\cdot \sigma _{k,j}(x)dxdW^{k,j}. \end{aligned}$$

Secondly, let us observe that for each $x\in \mathbb {T}^3,\ t\in [0,T]$

$$\begin{aligned} (\sigma _{k,j}\cdot \nabla B^{\varepsilon })\otimes (B^{\varepsilon }\cdot \nabla \sigma _{-k,j})+(\sigma _{-k,j}\cdot \nabla B^{\varepsilon })\otimes (B^{\varepsilon }\cdot \nabla \sigma _{k,j})&=0,\\ (B^{\varepsilon }\cdot \nabla \sigma _{k,j})\otimes (\sigma _{-k,j}\cdot \nabla B^{\varepsilon })+(B^{\varepsilon }\cdot \nabla \sigma _{-k,j})\otimes (\sigma _{k,j}\cdot \nabla B^{\varepsilon })&=0. \end{aligned}$$

Therefore

4.7 $$\begin{aligned} \begin{array}{c} \sum _{\begin{array}{c} k\in \mathbb {Z}^{3}_0\\ j\in \{1,2\} \end{array}}\int _0^t \int _{\mathbb {T}^3}Tr\left( \nabla ^2_b f(x,B^{\varepsilon }_s(x))(\sigma _{k,j}(x)\cdot \nabla B^{\varepsilon }_s(x))\otimes (B^{\varepsilon }_s(x)\cdot \nabla \sigma _{-k,j}(x))\right) dx ds \\ +\sum _{\begin{array}{c} k\in \mathbb {Z}^{3}_0\\ j\in \{1,2\} \end{array}}\int _0^t \int _{\mathbb {T}^3} Tr\left( \nabla ^2_bf(x,B^{\varepsilon }_s(x))(B^{\varepsilon }_s(x)\cdot \nabla \sigma _{k,j}(x))\otimes (\sigma _{-k,j}(x)\cdot \nabla B^{\varepsilon }_s(x))\right) dx ds=0. \end{array}\end{aligned}$$

Considering $$\sum _{\begin{array}{c} k\in \mathbb {Z}^{3}_0\\ j\in \{1,2\} \end{array}}\int _0^t\int _{\mathbb {T}^3}Tr\left( \nabla ^2_b f(x,B^{\varepsilon }_s(x))(B^{\varepsilon }_s(x)\cdot \nabla \sigma _{k,j}(x))\otimes (B^{\varepsilon }_s(x)\right. \left. \cdot \nabla \sigma _{-k,j}(x))\right) dxds $$, we observe that for each $x\in \mathbb {T}^3,\ t\in [0,T]$

$$\begin{aligned}&\sum _{\begin{array}{c} k\in \mathbb {Z}^{3}_0\\ j\in \{1,2\} \end{array}}Tr\left( \nabla ^2_b f(\cdot ,B^{\varepsilon })(B^{\varepsilon }\cdot \nabla \sigma _{k,j}\otimes B^{\varepsilon }\cdot \nabla \sigma _{-k,j}\right) \\  &\quad =\sum _{\begin{array}{c} k\in \mathbb {Z}^3_0\\ n\le |k|\le 2n \end{array}}\sum _{\alpha ,\beta =1}^3\frac{(B^{\varepsilon }\cdot k)^2}{|k|^5} \left( I-\frac{k\otimes k}{|k|^2}\right) _{\alpha ,\beta }\partial _{b,\alpha ,\beta } f(\cdot , B^{\varepsilon }) \end{aligned}$$

and by definition of $\mathcal {L}$

$$\begin{aligned} {{\,\textrm{div}\,}}_b\left( \mathcal {L}(B^{\varepsilon })\nabla _b f(\cdot ,B^\varepsilon )\right)&=\sum _{\begin{array}{c} k\in \mathbb {Z}^3_0\\ n\le |k|\le 2n \end{array}}\sum _{\alpha ,\beta =1}^3\partial _{b,\alpha }\left( \frac{\left( B^\varepsilon \cdot k\right) ^2}{|k|^5}\left( I-\frac{k\otimes k}{|k|^2}\right) _{\alpha ,\beta }\partial _{b,\beta }f(\cdot ,B^{\varepsilon })\right) \\  &= 2\sum _{\begin{array}{c} k\in \mathbb {Z}^3_0\\ n\le |k|\le 2n \end{array}} \frac{B^\varepsilon \cdot k }{|k|^5} k\cdot \left( I-\frac{k\otimes k}{|k|^2}\right) \nabla _b f(\cdot ,B^{\varepsilon })) \\  &\quad +\sum _{\begin{array}{c} k\in \mathbb {Z}^3_0\\ n\le |k|\le 2n \end{array}}\sum _{\alpha ,\beta =1}^3\frac{(B^{\varepsilon }\cdot k)^2}{|k|^5} \left( I-\frac{k\otimes k}{|k|^2}\right) _{\alpha ,\beta }\partial _{b,\alpha ,\beta } f(\cdot , B^{\varepsilon })\\  &=\sum _{\begin{array}{c} k\in \mathbb {Z}^3_0\\ n\le |k|\le 2n \end{array}}\sum _{\alpha ,\beta =1}^3\frac{(B^{\varepsilon }\cdot k)^2}{|k|^5} \left( I-\frac{k\otimes k}{|k|^2}\right) _{\alpha ,\beta }\partial _{b,\alpha ,\beta } f(\cdot , B^{\varepsilon }). \end{aligned}$$

Therefore

4.8 $$\begin{aligned}  &   \sum _{\begin{array}{c} k\in \mathbb {Z}^{3}_0\\ j\in \{1,2\} \end{array}}\int _0^t\int _{\mathbb {T}^3}Tr\left( \nabla ^2_b f(x,B^{\varepsilon }_s(x))(B^{\varepsilon }_s(x){\!}\cdot {\!}\nabla \sigma _{k,j}(x)){\!}\otimes {\!}(B^{\varepsilon }_s(x)\cdot {\!}\nabla \sigma _{-k,j}(x))\right) dxds\nonumber \\    &   \quad =\int _0^t \int _{\mathbb {T}^3} {{\,\textrm{div}\,}}_b\left( \mathcal {L}(B^{\varepsilon }(x))\nabla _b f(x,B^\varepsilon (x))\right) dx ds. \end{aligned}$$

Lastly we note that for each $\alpha ,\beta \in \{1,2,3\}$

$$\begin{aligned} \sum _{\begin{array}{c} k\in \mathbb {Z}^{3}_0\\ j\in \{1,2\} \end{array}}(\sigma _{k,j}\cdot \nabla B^{\varepsilon })_{\alpha } (\sigma _{k,j}\cdot \nabla B^{\varepsilon })_{\beta }&=\frac{2}{3}\eta \sum _{\gamma =1}^3 \partial _\gamma (B^{\varepsilon })_{\alpha }\partial _\gamma (B^{\varepsilon })_{\beta }. \end{aligned}$$

Therefore, by the chain rule and integrating by parts repeatedly

4.9 $$\begin{aligned}&\frac{2}{3}\eta \int _0^t \int _{\mathbb {T}^3}\nabla _b f(x,B^{\varepsilon }_s(x))\cdot \Delta B^{\varepsilon }_s(x)dxds\nonumber \\  &\qquad +\sum _{\begin{array}{c} k\in \mathbb {Z}^{3}_0\\ j\in \{1,2\} \end{array}}\int _0^t\int _{\mathbb {T}^3}Tr\left( \nabla ^2_b f(x,B^{\varepsilon }_s(x))(\sigma _{k,j}(x)\cdot \nabla B^{\varepsilon }_s(x))\right. \nonumber \\&\quad \quad \quad \left. \otimes (\sigma _{-k,j}(x)\cdot \nabla B^{\varepsilon }_s(x))\right) dxds \nonumber \\  &\quad = \frac{2}{3}\eta \sum _{\alpha ,\beta =1}^3\int _0^t\int _{\mathbb {T}^3} \partial _{x,\alpha }(\partial _{b,\beta }f(x,B^{\varepsilon }_s(x))\partial _{\alpha }(B^{\varepsilon }_s(x))_{\beta })\nonumber \\&\quad \quad \quad \quad -(\partial _{x,\alpha }\partial _{b,\beta }f)(x,B^{\varepsilon }_s(x))\partial _{\alpha }(B^{\varepsilon }_s(x))_{\beta } dx ds\nonumber \\  &\quad =-\frac{2}{3}\eta \sum _{\alpha ,\beta =1}^3\int _0^t\int _{\mathbb {T}^3} \partial _{x,\alpha }\left( \partial _{x,\alpha }f(x,B^{\varepsilon }_s(x))\right) dx ds\nonumber \\&\quad +\frac{2}{3}\eta \int _0^t \int _{\mathbb {T}^3}(\Delta _x f)(x,B^{\varepsilon }_s(x)) dx ds\nonumber \\  &\quad = \frac{2}{3}\eta \int _0^t \int _{\mathbb {T}^3}(\Delta _x f)(x,B^{\varepsilon }_s(x)) dx ds. \end{aligned}$$

Integrating (4.5) on $\mathbb {T}^3$, thanks to (4.6), (4.7), (4.8) and (4.9) the claim follows. □

Now we are in the position to prove the validity of relation (4.2).

#### Proof of (4.2)

Let us start considering $f\in C^{3}_b(\mathbb {T}^3\times \mathbb {R}^3)$ and t∈[0,T]. Since $B\in C([0,T];V_w)$ $\mathbb {P}-a.s.$ then for each t∈[0,T] $B^{\varepsilon }(t)\rightarrow B(t)$ in $V\hookrightarrow L^p(\mathbb {T}^3;\mathbb {R}^3)$ for all p≤6 and

4.10 $$\begin{aligned} \sup _{t\in [0,T]}\left\| B^{\varepsilon }(t)\right\| _V\le \sup _{t\in [0,T]}\left\| B(t)\right\| _V<+\infty \quad \mathbb {P}-a.s. \end{aligned}$$

Due to Lemma 4.3 it is enough to pass to the limit in each term appearing in the lemma. Thanks to the regularity of *f* we get easily

$$\begin{aligned} \left\lvert \int _{\mathbb {T}^3}f(x,B^{\varepsilon }_t(x))-f(x,B_t(x)) dx\right\rvert&\le \left\| f\right\| _{C^1_b}\left\| B^{\varepsilon }_t-B_t\right\| _{L^1(\mathbb {T}^3)}\rightarrow 0\quad \mathbb {P}-a.s. \end{aligned}$$

This gives us the convergence of the terms non integrated in time. Secondly let us study the deterministic integrals affecting the limit. Due to the regularity of *f* and (4.10) we have

$$\begin{aligned} \left\lvert \int _{\mathbb {T}^3}(\Delta _x f)(x,B^{\varepsilon }_t(x))dx\right\rvert&\lesssim \left\| f\right\| _{C^2_b}\in L^1(0,T)\quad \mathbb {P}-a.s.,\\ \left\lvert \int _{\mathbb {T}^3}{{\,\textrm{div}\,}}_b\left( \mathcal {L}(B^{\varepsilon }_t(x))\nabla _b f(x,B^\varepsilon _t(x))\right) dx\right\rvert&\lesssim \left\| f\right\| _{C^2_b}\left\| B\right\| _{C([0,T];H)}^2\in L^1(0,T)\quad \mathbb {P}-a.s., \\ \left\lvert \int _{\mathbb {T}^3}(\Delta _x f)(x,B^{\varepsilon }_t(x))-(\Delta _x f)(x,B_t(x)) dx\right\rvert&\le \left\| f\right\| _{C^3_b}\left\| B^{\varepsilon }_t-B_t\right\| _{L^1(\mathbb {T}^3)}\rightarrow 0\quad \mathbb {P}-a.s., \end{aligned}$$

$$\begin{aligned}  &   \left\lvert \int _{\mathbb {T}^3}{{\,\textrm{div}\,}}_b\left( \mathcal {L}(B^{\varepsilon }_t(x))\nabla _b f(x,B^\varepsilon _t(x))-\mathcal {L}(B_t(x))\nabla _b f(x,B_t(x))\right) dx\right\rvert \\    &   \quad \le \left\lvert \int _{\mathbb {T}^3}{{\,\textrm{div}\,}}_b\left( \mathcal {L}(B^{\varepsilon }_t(x))\nabla _b f(x,B^\varepsilon _t(x))-\mathcal {L}(B^{\varepsilon }_t(x))\nabla _b f(x,B_t(x))\right) dx\right\rvert \\    &   \qquad + \left\lvert \int _{\mathbb {T}^3}{{\,\textrm{div}\,}}_b\left( \mathcal {L}(B_t(x))\nabla _b f(x,B^\varepsilon _t(x))-\mathcal {L}(B_t(x))\nabla _b f(x,B_t(x))\right) dx\right\rvert \\    &   \quad \lesssim \left\| f\right\| _{C^3_b}\left\| B^{\varepsilon }_t\right\| _{L^3(\mathbb {T}^3)}^2\left\| B^{\varepsilon }_t-B_t\right\| _{L^3(\mathbb {T}^3)}\\  &   \quad +\left\| f\right\| _{C^2_b}\left\| B^{\varepsilon }_t-B_t\right\| _{L^2(\mathbb {T}^3)}\left\| B^{\varepsilon }_t+B_t\right\| _{L^2(\mathbb {T}^3)}\rightarrow 0\quad \mathbb {P}-a.s. \end{aligned}$$

Therefore by dominated convergence theorem we get the convergence of the corresponding terms. The analysis of the stochastic integrals affecting the limit goes in a similar way. Indeed by Itô isometry

$$\begin{aligned}&\mathbb {E} \left[ \left\lvert \sum _{\begin{array}{c} k\in \mathbb {Z}^{3}_0\\ j\in \{1,2\} \end{array}}\int _0^t\int _{\mathbb {T}^3}\left( B^\varepsilon _s(x)\cdot \nabla \sigma _{k,j}(x)\nabla _b f(x,B^\varepsilon _s(x))-B_s(x)\cdot \nabla \sigma _{k,j}(x)\nabla _b f(x,B_s(x))\right) dx dW^{k,j}_s\right\rvert ^2\right] \\  &= \mathbb {E} \left[ \int _0^t\sum _{\begin{array}{c} k\in \mathbb {Z}^{3}_0\\ j\in \{1,2\} \end{array}}\left\lvert \int _{\mathbb {T}^3}\left( B^\varepsilon _s(x)\cdot \nabla \sigma _{k,j}(x)\nabla _b f(x,B^\varepsilon _s(x))-B_s(x)\cdot \nabla \sigma _{k,j}(x)\nabla _b f(x,B_s(x))\right) dx\right\rvert ^2 ds\right] ,\\&\mathbb {E} \left[ \left\lvert \sum _{\begin{array}{c} k\in \mathbb {Z}^{3}_0\\ j\in \{1,2\} \end{array}}\int _0^t\int _{\mathbb {T}^3}\left( \sigma _{k,j}(x)\cdot (\nabla _x f)(x,B^\varepsilon _s(x))-\sigma _{k,j}(x)\cdot (\nabla _x f)(x,B_s(x))\right) dx dW^{k,j}_s\right\rvert ^2\right] \\  &=\mathbb {E} \left[ \int _0^t\sum _{\begin{array}{c} k\in \mathbb {Z}^{3}_0\\ j\in \{1,2\} \end{array}}\left\lvert \int _{\mathbb {T}^3}\left( \sigma _{k,j}(x)\cdot (\nabla _x f)(x,B^\varepsilon _s(x))-\sigma _{k,j}(x)\cdot (\nabla _x f)(x,B_s(x))\right) dx\right\rvert ^2 ds\right] . \end{aligned}$$

Again due to Theorem 2.10, (4.10) and the regularity of *f*

$$\begin{aligned}&\sum _{\begin{array}{c} k\in \mathbb {Z}^{3}_0\\ j\in \{1,2\} \end{array}}\left\lvert \int _{\mathbb {T}^3}\left( B^\varepsilon _t(x)\cdot \nabla \sigma _{k,j}(x)\nabla _b f(x,B^\varepsilon _t(x))-B_t(x)\cdot \nabla \sigma _{k,j}(x)\nabla _b f(x,B_t(x))\right) dx\right\rvert ^2\\  &\quad \lesssim \left\| f\right\| _{C^1_b}^2 \left\| B\right\| _{C([0,T];H)}^2\in L^1(\Omega \times (0,T)),\\&\sum _{\begin{array}{c} k\in \mathbb {Z}^{3}_0\\ j\in \{1,2\} \end{array}}\left\lvert \int _{\mathbb {T}^3}\left( \sigma _{k,j}(x)\cdot (\nabla _x f)(x,B^\varepsilon _t(x))-\sigma _{k,j}(x)\cdot (\nabla _x f)(x,B_t(x))\right) dx\right\rvert ^2 \\&\quad \lesssim \left\| f\right\| _{C^1_b}^2 \in L^1(\Omega \times (0,T)) \end{aligned}$$

and

$$\begin{aligned}&\sum _{\begin{array}{c} k\in \mathbb {Z}^{3}_0\\ j\in \{1,2\} \end{array}}\left\lvert \int _{\mathbb {T}^3}\left( \sigma _{k,j}(x)\cdot (\nabla _x f)(x,B^\varepsilon _t(x))-\sigma _{k,j}(x)\cdot (\nabla _x f)(x,B_t(x))\right) dx\right\rvert ^2\\  &\quad \lesssim \left\| f\right\| ^2_{C^2_b}\left\| B^\varepsilon _t-B_t\right\| ^2\rightarrow 0\quad a.e.\quad (\omega ,t)\in \Omega \times (0,T) , \end{aligned}$$

$$\begin{aligned}&\sum _{\begin{array}{c} k\in \mathbb {Z}^{3}_0\\ j\in \{1,2\} \end{array}}\left\lvert \int _{\mathbb {T}^3}\left( B^\varepsilon _t(x)\cdot \nabla \sigma _{k,j}(x)\nabla _b f(x,B^\varepsilon _t(x))-B_t(x)\cdot \nabla \sigma _{k,j}(x)\nabla _b f(x,B_t(x))\right) dx\right\rvert ^2\\  &\quad \lesssim \sum _{\begin{array}{c} k\in \mathbb {Z}^{3}_0\\ j\in \{1,2\} \end{array}}\left\lvert \int _{\mathbb {T}^3}\left( B^\varepsilon _t(x)\cdot \nabla \sigma _{k,j}(x)\nabla _b f(x,B^\varepsilon _t(x))-B^{\varepsilon }_t(x)\cdot \nabla \sigma _{k,j}(x)\nabla _b f(x,B_t(x))\right) dx\right\rvert ^2\\  &\qquad +\sum _{\begin{array}{c} k\in \mathbb {Z}^{3}_0\\ j\in \{1,2\} \end{array}}\left\lvert \int _{\mathbb {T}^3}\left( B^\varepsilon _t(x)\cdot \nabla \sigma _{k,j}(x)\nabla _b f(x,B(x))-B_t(x)\cdot \nabla \sigma _{k,j}(x)\nabla _b f(x,B_t(x))\right) dx\right\rvert ^2\\  &\quad \lesssim \left\| f\right\| ^2_{C^2_b}\left\| B_t\right\| _{L^2}^2 \left\| B^{\varepsilon }_t-B_t\right\| _{L^2}^2+\left\| f\right\| _{C^1_b}^2 \left\| B^{\varepsilon }_t-B_t\right\| _{L^1}^2\rightarrow 0\quad a.e.\quad (\omega ,t)\in \Omega \times (0,T). \end{aligned}$$

Therefore by dominated convergence theorem we get the convergence of the corresponding terms. Up to passing to subsequences the convergence is $\mathbb {P}-a.s.$ We are left to show that the remaining terms $\mathbb {P}-a.s.$ converge to 0. We start with the deterministic integrals. Thanks to (4.10) and the regularity of *f*

$$\begin{aligned}\begin{array}{c} \sum _{\begin{array}{c} k\in \mathbb {Z}^{3}_0\\ j\in \{1,2\} \end{array}}\Bigg |\int _{\mathbb {T}^3}Tr\left( \nabla ^2_b f(x,B^{\varepsilon }_t(x))\left( \left( [\sigma _{k,j}\cdot \nabla , \varrho _{\varepsilon }]+[\cdot \nabla \sigma _{k,j}, \varrho _{\varepsilon } ]\right) B_t(x)\right. \right. \\ \otimes \left. \left. \left( [\sigma _{-k,j}\cdot \nabla , \varrho _{\varepsilon }]+[\cdot \nabla \sigma _{-k,j}, \varrho _{\varepsilon } ]\right) B_t(x)\right) \right) dx \Bigg |\\ \lesssim \left\| f\right\| _{C^2_b}\sum _{\begin{array}{c} k\in \mathbb {Z}^{3}_0\\ j\in \{1,2\} \end{array}}\left( \left\| [\sigma _{k,j}\cdot \nabla ,\varrho _{\varepsilon }]B\right\| _{L^2(\mathbb {T}^3)}^2+\left\| [\cdot \nabla \sigma _{k,j},\varrho _{\varepsilon }]B\right\| _{L^2(\mathbb {T}^3)}^2\right) , \end{array}\end{aligned}$$

$$\begin{aligned}\begin{array}{c} \sum _{\begin{array}{c} k\in \mathbb {Z}^{3}_0\\ j\in \{1,2\} \end{array}}\left\lvert \int _{\mathbb {T}^3}Tr\left( \nabla ^2_b f(x,B^{\varepsilon }_t(x))\left( \left( [\sigma _{k,j}\cdot \nabla , \varrho _{\varepsilon }]+[\cdot \nabla \sigma _{k,j}, \varrho _{\varepsilon } ]\right) B_t(x)\right. \right. \right. \\ \left. \left. \otimes \left( \sigma _{-k,j}(x)\cdot \nabla B^{\varepsilon }_t(x)-B^{\varepsilon }_t(x)\cdot \nabla \sigma _{-k,j}(x)\right) \right) \right) dx \bigg |\\ \lesssim \left\| f\right\| _{C^2_b}\sup _{t\in [0,T]}\left\| B_t\right\| _{V}\sum _{\begin{array}{c} k\in \mathbb {Z}^{3}_0\\ j\in \{1,2\} \end{array}}\left( \left\| [\sigma _{k,j}\cdot \nabla ,\varrho _{\varepsilon }]B\right\| _{L^2(\mathbb {T}^3)}+\left\| [\cdot \nabla \sigma _{k,j},\varrho _{\varepsilon }]B\right\| _{L^2(\mathbb {T}^3)}\right) . \end{array}\end{aligned}$$

As a consequence of Theorem 2.10, Lemma 4.2 the terms above converge pointwise to 0 and are uniformly bounded by a function in $L^1(0,T)$. Therefore dominated convergence theorem implies the claim. Concerning the last stochastic integral, by Itô isometry

$$\begin{aligned}&\mathbb {E} \left[ \left\lvert \sum _{\begin{array}{c} k\in \mathbb {Z}^{3}_0\\ j\in \{1,2\} \end{array}}\int _0^t \int _{\mathbb {T}^3}\left( [\sigma _{k,j}\cdot \nabla , \varrho _{\varepsilon }]+[\cdot \nabla \sigma _{k,j}, \varrho _{\varepsilon } ]\right) B_s(x)\cdot \nabla _b f(x,B^{\varepsilon }_s) dx dW^{k,j}_s \right\rvert ^2\right] \\  &= \mathbb {E} \left[ \int _0^t \sum _{\begin{array}{c} k\in \mathbb {Z}^{3}_0\\ j\in \{1,2\} \end{array}}\left\lvert \int _{\mathbb {T}^3}\left( [\sigma _{k,j}\cdot \nabla , \varrho _{\varepsilon }]+[\cdot \nabla \sigma _{k,j}, \varrho _{\varepsilon } ]\right) B_s(x)\cdot \nabla _b f(x,B^{\varepsilon }_s) dx \right\rvert ^2 ds \right] . \end{aligned}$$

If we show that the latter term converges to 0 as $\varepsilon \rightarrow 0$ then by passing to subsequences we have the required $\mathbb {P}-a.s.$ convergence. Thanks to the regularity of *f*

$$\begin{aligned}\begin{array}{c} \sum _{\begin{array}{c} k\in \mathbb {Z}^{3}_0\\ j\in \{1,2\} \end{array}}\left\lvert \int _{\mathbb {T}^3}\left( [\sigma _{k,j}\cdot \nabla , \varrho _{\varepsilon }]+[\cdot \nabla \sigma _{k,j}, \varrho _{\varepsilon } ]\right) B_t(x)\cdot \nabla _b f(x,B^{\varepsilon }_t) dx \right\rvert ^2\\ \lesssim \left\| f\right\| _{C^1_b}^2\sum _{\begin{array}{c} k\in \mathbb {Z}^{3}_0\\ j\in \{1,2\} \end{array}}\left( \left\| [\sigma _{k,j}\cdot \nabla ,\varrho _{\varepsilon }]B\right\| _{L^2(\mathbb {T}^3)}^2+\left\| [\cdot \nabla \sigma _{k,j},\varrho _{\varepsilon }]B\right\| _{L^2(\mathbb {T}^3)}^2\right) . \end{array}\end{aligned}$$

As a consequence of Theorem 2.10, Lemma 4.2 the terms above converge pointwise to 0 and are uniformly bounded by a function in $L^1(\Omega \times (0,T))$. Dominated convergence theorem’s implies the validity of the claim.

So far we showed that for each t∈[0,T] and $f\in C^3_b(\mathbb {T}^3\times \mathbb {R}^3)$ there exists a zero measure set $\mathcal {N}\subset \Omega$ such that on its complementary

$$\begin{aligned}&\int _{\mathbb {T}^3}f(x,B_t(x))-f(x,\overline{B}_0(x)) dx\\  &\quad =-\sum _{\begin{array}{c} k\in \mathbb {Z}^{3}_0\\ j\in \{1,2\} \end{array}}\int _0^t\int _{\mathbb {T}^3} \sigma _{k,j}(x)\cdot \left( \nabla _x f\right) (x,B_s(x))dW^{k,j}_s\\  &\quad -\sum _{\begin{array}{c} k\in \mathbb {Z}^{3}_0\\ j\in \{1,2\} \end{array}}\int _0^t\int _{\mathbb {T}^3}B_s(x)\cdot \nabla \sigma _{k,j}(x)\nabla _b f(x,B_s(x))dW^{k,j}_s \nonumber \\  &\quad +\frac{2}{3}\eta \int _0^t\int _{\mathbb {T}^3} \left( \Delta _x f\right) (x,B_s(x)) dxds\\  &\quad +\int _0^t\int _{\mathbb {T}^3} {{\,\textrm{div}\,}}_b\left( \mathcal {L}(B_s(x))\nabla _b f(x,B_s(x))\right) dx ds. \end{aligned}$$

Thanks to the density of $C^{3}_b(\mathbb {T}^3\times \mathbb {R}^3)$ in $C^{2}_b(\mathbb {T}^3\times \mathbb {R}^3)$ and the continuity properties of *B*, *cf. * Theorem 2.10, the relation above holds for each $f\in C^2_b(\mathbb {T}^3\times \mathbb {R}^3)$ $\mathbb {P}-a.s.$ uniformly in t∈[0,T]. Recalling the definition of μ(t,x,db) this completes the proof of (4.2). □

### Uniform estimates

Now we are interested to study the regularity of the increments of $\int _{T^3\times \mathbb {R}^3} f(x,b)\mu _n(t,x,db)dx$. The following holds:

#### Proposition 4.4

For each $f\in C^2_b(\mathbb {T}^3\times \mathbb {R}^3)$ and $\gamma \ge 1$ we have

$$\begin{aligned}&\mathbb {E} \left[ \left\lvert \int _{T^3\times \mathbb {R}^3} f(x,b)(\mu _n(t,x,db)-\mu _n(s,x,db))dx \right\rvert ^{\gamma }\right] \\  &\quad \le C_2(\overline{B}_0,T,2\gamma )\left\| f\right\| _{C^2_b(\mathbb {T}^3\times \mathbb {R}^3)}^{\gamma }|t-s|^{\gamma /2}. \end{aligned}$$

#### Proof

Due to Remark 4.1 we have

$$\begin{aligned}&\mathbb {E} \left[ \left\lvert \int _{\mathbb {T}^3}f(x,B^n_t(x))-f(x,B^n_s(x)) dx\right\rvert ^{\gamma }\right] \\&\quad \lesssim _{\gamma }\mathbb {E} \left[ \left\lvert \sum _{\begin{array}{c} k\in \mathbb {Z}^{3}_0\\ j\in \{1,2\} \end{array}}\int _s^t\int _{\mathbb {T}^3} \sigma _{k,j}^{n}(x)\cdot \left( \nabla _x f\right) (x,B^n_r(x))dxdW^{k,j}_r \right\rvert ^\gamma \right] \\&\quad +\mathbb {E} \left[ \left\lvert \sum _{\begin{array}{c} k\in \mathbb {Z}^{3}_0\\ j\in \{1,2\} \end{array}}\int _s^t\int _{\mathbb {T}^3}B^n_r(x)\cdot \nabla \sigma _{k,j}^{n}(x)\nabla _b f(x,B^n_r(x))dxdW^{k,j}_r\right\rvert ^\gamma \right] \\&\quad +\left( \frac{2}{3}\eta _n\right) ^{\gamma }\mathbb {E} \left[ \left\lvert \int _s^t\int _{\mathbb {T}^3} \left( \Delta _x f\right) (x,B^n_r(x)) dxdr\right\rvert ^\gamma \right] \\&\quad +\mathbb {E} \left[ \left\lvert \int _s^t\int _{\mathbb {T}^3} {{\,\textrm{div}\,}}_b\left( \mathcal {L}^n(B^n_r(x))\nabla _b f(x,B^n_r(x))\right) dx dr\right\rvert ^\gamma \right] \\&\quad =J_1+J_2+J_3+J_4. \end{aligned}$$

We have immediately

4.11 $$\begin{aligned} J_3\lesssim \left\| \nabla ^2_x f\right\| _{C_b}^{\gamma }|t-s|^{\gamma }. \end{aligned}$$

Similarly we can treat $J_4$. Indeed, since $k\cdot \left( I-\frac{k\otimes k}{|k|^2}\right) =0$, we obtain

4.12 $$\begin{aligned} J_4&\lesssim _{\gamma }\mathbb {E} \left[ \left\lvert \int _s^t\int _{\mathbb {T}^3} Tr\left( \mathcal {L}^n(B^n_r(x))\nabla ^2_b f(x,B^n_r(x))\right) dx dr\right\rvert ^\gamma \right] \nonumber \\  &\lesssim \mathbb {E} \left[ \left\lvert \int _s^t\int _{\mathbb {T}^3} |B^n_r(x)|^2 \sum _{\begin{array}{c} k\in \mathbb {Z}^3_0\\ n\le |k|\le 2n \end{array}}\frac{1}{|k|^3}\left\| \nabla ^2_b f\right\| _{C_b} dx dr\right\rvert ^\gamma \right] \nonumber \\  &\lesssim \alpha _n^{\gamma }\vert t-s\vert ^\gamma \left\| \nabla ^2_b f\right\| _{C_b}^{\gamma }\mathbb {E} \left[ \sup _{t\in [0,T]}\left\| B^n_t\right\| ^{2\gamma }\right] \nonumber \\  &\lesssim C_1(\overline{B}_0,T,2\gamma )\left\| \nabla ^2_b f\right\| _{C_b}^{\gamma }|t-s|^{\gamma }. \end{aligned}$$

Lastly $J_1$ and $J_2$ can be treated by Burkholder-Davis-Gundy inequality. Indeed,

4.13 $$\begin{aligned} J_1&\lesssim _{\gamma }\mathbb {E} \left[ \left( \sum _{\begin{array}{c} k\in \mathbb {Z}^{3}_0\\ j\in \{1,2\} \end{array}}\int _s^t \left( \int _{\mathbb {T}^3} \sigma _{k,j}^{n}(x)\cdot \left( \nabla _x f\right) (x,B^n_r(x))dx\right) ^2 dr\right) ^{\gamma /2}\right] \nonumber \\  &\lesssim _{\gamma } \eta _n^{\gamma /2}\left\| \nabla _x f\right\| _{C_b}^\gamma |t-s|^{\gamma /2}. \end{aligned}$$

Finally

4.14 $$\begin{aligned} J_2&\lesssim _{\gamma } \mathbb {E} \left[ \left( \sum _{\begin{array}{c} k\in \mathbb {Z}^{3}_0\\ j\in \{1,2\} \end{array}}\int _s^t \left( \int _{\mathbb {T}^3} B^n_r(x)\cdot \nabla \sigma _{k,j}^{n}(x)\nabla _b f(x,B^n_r(x))dx\right) ^2 dr\right) ^{\gamma /2}\right] \nonumber \\  &\lesssim \alpha _n^{\gamma /2}\mathbb {E} \left[ \sup _{t\in [0,T]}\left\| B^n_t\right\| ^{\gamma }\right] \left\| \nabla _b f\right\| _{C_b}^\gamma |t-s|^{\gamma /2}\nonumber \\  &\lesssim \alpha _n^{\gamma /2}C_{1}(\overline{B}_0,T,\gamma )\left\| \nabla _b f\right\| _{C_b}^\gamma |t-s|^{\gamma /2}. \end{aligned}$$

Combining (4.13), (4.14), (4.11), (4.12) the result follows. □

As an immediate corollary of Proposition 4.4 and of the Garsia–Rodemich–Rumsey inequality, see [22], we obtain

#### Corollary 4.5

For each $f\in C^2_b(\mathbb {T}^3\times \mathbb {R}^3),\ \phi ,\lambda$ such that $\phi <\frac{1}{2},\ \phi -\frac{1}{\lambda }>0$ then

$$\begin{aligned} \int _{\mathbb {T}^3\times \mathbb {R}^3}f(x,b)\mu _n(t,x,db)dx \end{aligned}$$

has a $\phi -\frac{1}{\lambda }$ Hölder version. Moreover

$$\begin{aligned} \mathbb {E} \left[ \left\| \int _{\mathbb {T}^3\times \mathbb {R}^3}f(x,b)\mu _n(t,x,db)dx\right\| _{C^{0,\phi -\frac{1}{\lambda }}}^{\lambda }\right] \le C(\phi ,\lambda ,\overline{B}_0,T)\left\| f\right\| _{C^2_b}^{\lambda }. \end{aligned}$$

#### Proof

Thanks to Garsia–Rodemich–Rumsey inequality we know that for each *t*, *s*

4.15 $$\begin{aligned} \begin{array}{c} \frac{\left\lvert \int _{\mathbb {T}^3\times \mathbb {R}^3}f(x,b)\left( \mu _n(t,x,db)-\mu _n(s,x,db)\right) dx\right\rvert ^{\lambda }}{|t-s|^{\phi \lambda -1}}\\ \le C(\phi ,\lambda )\int _s^t\int _s^t \frac{\left\lvert \int _{\mathbb {T}^3\times \mathbb {R}^3}f(x,b)\left( \mu _n(r,x,db)-\mu _n(v,x,db)\right) dx\right\rvert ^{\lambda }}{|r-v|^{\phi \lambda +1}}dr dv. \end{array}\end{aligned}$$

Therefore, combining (4.15) and Proposition 4.4 we obtain

$$\begin{aligned}&\mathbb {E} \left[ \left\| \int _{T^3\times \mathbb {R}^3}f(x,b)\mu _n(t,x,db)dx\right\| _{C^{0,\phi -\frac{1}{\lambda }}}^{\lambda }\right] \\&\quad \lesssim _{\lambda } \left\| f\right\| _{C_b}^{\lambda }+\int _0^T\int _0^T \frac{C_2(\overline{B}_0,T,2\lambda )\left\| f\right\| _{C^2_b(\mathbb {T}^3\times \mathbb {R}^3)}^{\lambda }}{|r-v|^{\lambda (\phi -\frac{1}{2})+1}} dr dv \\&\quad \le C(\phi ,\lambda ,\overline{B}_0,T)\left\| f\right\| _{C^2_b}^{\lambda }. \end{aligned}$$

This completes the proof. □

### Compactness of the laws

The goal of this section is to show that the laws of $\mu _n$ are tight in the space of probability measure on $C([0,T];\mathcal {Y}).$ We start introducing a family of mollifiers on $\mathbb {T}^3\times \mathbb {R}^3$, i.e. we introduce

$$\begin{aligned}&h:\mathbb {T}^3\times \mathbb {R}^3\rightarrow \mathbb {R}\quad s.t.\ \operatorname {supp}h\subset U_{1/2}(\textbf{0}),\ h\in C^{\infty }(\mathbb {T}^3\times \mathbb {R}^3),\\&\quad h\ge 0, \int _{\mathbb {T}^3\times \mathbb {R}^3}h(\textbf{x})d\textbf{x}=1 \end{aligned}$$

and set $h_\varepsilon :\mathbb {T}^3\times \mathbb {R}^3\rightarrow \mathbb {R},\ h_{\varepsilon }(\textbf{x})=\frac{1}{\varepsilon ^6}h(\frac{\textbf{x}}{\varepsilon })$. Above we denoted by $U_{1/2}(\textbf{0})\subset \mathbb {T}^3\times \mathbb {R}^3$ the open ball in $\mathbb {T}^3\times \mathbb {R}^3$ centered in 0 and with radius $\frac{1}{2}.$ We recall that if $f\in C_{b}(\mathbb {T}^3\times \mathbb {R}^3)$ is uniformly continuous then

4.16 $$\begin{aligned} f^{\varepsilon }&:=h_{\varepsilon }*f\rightarrow f\quad \text {in } C_{b}(\mathbb {T}^3\times \mathbb {R}^3),\quad \left\| f^{\varepsilon }\right\| _{C^1_b}\le \frac{\left\| f\right\| _{C_b}\left\| \nabla h\right\| _{W^{1,1}}}{\varepsilon },\nonumber \\  &\quad \left\| f^{\varepsilon }\right\| _{C^2_b}\le \frac{\left\| f\right\| _{C_b}\left\| h\right\| _{W^{2,1}}}{\varepsilon ^2}. \end{aligned}$$

Now we are ready to prove the main result of this section.

#### Theorem 4.6

The laws of $\mu _n$ are tight in the space of probability measure on $C([0,T];\mathcal {Y}).$

#### Proof

Thanks to Lemma 2.4 it is enough to show that for each δ>0 there exist a family of positive quantities $R(k,\delta ),\ \theta (k,\delta ,i),\ i,k\in \mathbb {N}$ such that

$$\begin{aligned} \mathbb {P}(\mu _n\notin \overline{K^{\delta }})\le \delta . \end{aligned}$$

By definition of $K^{\delta }$ we have thanks to Markov’s inequality

$$\begin{aligned} \mathbb {P}(\mu _n\notin \overline{K^{\delta }})&\le \mathbb {P}(\mu _n\notin {K^{\delta }})\\  &\le \sum _{k\in \mathbb {N}}\mathbb {P}\left( \sup _{t\in [0,T]}\int _{\mathbb {T}^3}^* \mu _n(t, x, U_{R(k,\delta )}(0)^c)dx> \frac{1}{k}\right) \\  &\quad +\sum _{i, k\in \mathbb {N}}\mathbb {P}\left( \sup _{|t-s|< \theta (k,\delta ,i)}\left\lvert \int _{\mathbb {T}^3\times \mathbb {R}^3} g_i(x,b) (\mu _n(t,x,db)-\mu _n(s,x,db))\right\rvert > \frac{1}{k}\right) \\  &\le \sum _{k\in \mathbb {N}} k\mathbb {E} \left[ \sup _{t\in [0,T]}\int _{\mathbb {T}^3}^* \mu _n(t,x, U_{R(k,\delta )}(0)^c)dx\right] \\  &\quad + \sum _{i, k\in \mathbb {N}}k\mathbb {E} \left[ \sup _{|t-s|< \theta (k,\delta ,i)}\left\lvert \int _{\mathbb {T}^3\times \mathbb {R}^3} g_i(x,b) (\mu _n(t,x,db)-\mu _n(s,x,db))\right\rvert \right] \\&=H_1+H_2. \end{aligned}$$

We recall that $U_{R(k,\delta )}(0)\subset \mathbb {R}^3$ is the open ball in $\mathbb {R}^3$ centered in 0 with radius R(k,δ). Thanks to the definition of $\mu _n$ and Theorem 2.10 we can easily treat $H_1$ by Markov’s inequality. Indeed,

4.17 $$\begin{aligned} H_1&\le \sum _{k\in \mathbb {N}} k\mathbb {E} \left[ \sup _{t\in [0,T]}\int _{\mathbb {T}^3}\boldsymbol{1}_{|B^n_t(x)|>R(k,\delta )}(x) dx \right] \nonumber \\  &\le |\mathbb {T}^3|\sum _{k\in \mathbb {N}} \frac{k}{R^2(k,\delta )}\mathbb {E} \left[ \sup _{t\in [0,T]}\left\| B^n_t\right\| ^2\right] \nonumber \\  &\le C_0(\overline{B}_0,T)|\mathbb {T}^3|\sum _{k\in \mathbb {N}} \frac{k}{R^2(k,\delta )}. \end{aligned}$$

The estimate of $H_2$ is more involved. Obviously we have

$$\begin{aligned}&\left\lvert \int _{\mathbb {T}^3\times \mathbb {R}^3} g_i(x,b) (\mu _n(t,x,db)-\mu _n(s,x,db))\right\rvert \\&\quad \le \left\lvert \int _{\mathbb {T}^3\times \mathbb {R}^3} g^{\varepsilon }_i(x,b) (\mu _n(t,x,db)-\mu _n(s,x,db))\right\rvert \\&\qquad + \int _{\mathbb {T}^3\times \mathbb {R}^3}|g_i(x,b)-g^{\varepsilon }_i(x,b)||\mu _n(t,x,db)-\mu _n(s,x,db)|\\&\quad \le |\mathbb {T}^3|\left\| g_i-g_i^{\varepsilon }\right\| _{C_b}\\&\qquad +\left\lvert \int _{\mathbb {T}^3\times \mathbb {R}^3} g^{\varepsilon }_i(x,b) (\mu _n(t,x,db)-\mu _n(s,x,db))\right\rvert . \end{aligned}$$

Due to (4.16) for each $i\in \mathbb {N}$ we can choose $\varepsilon =\varepsilon (k,\delta ,i)$ such that

$$\begin{aligned} |\mathbb {T}^3|\left\| g_i-g_i^{\varepsilon }\right\| _{C_b}\le \frac{3\delta }{2^{i+1} \pi ^2 k^3}. \end{aligned}$$

Therefore we obtain

$$\begin{aligned} H_2\le \frac{\delta }{4}+\sum _{i, k\in \mathbb {N}}k\mathbb {E} \left[ \sup _{|t-s|< \theta (k,\delta ,i)}\left\lvert \int _{\mathbb {T}^3\times \mathbb {R}^3} g^{\varepsilon }_i(x,b) (\mu _n(t,x,db)-\mu _n(s,x,db))\right\rvert \right] . \end{aligned}$$

Lastly due to Corollary 4.5, with the choice of $\phi =\frac{3}{8},\ \gamma =8$, and (4.16) we have

4.18 $$\begin{aligned} H_2\le \frac{\delta }{4}+\frac{C(\overline{B}_0,T)\left\| h\right\| _{W^{2,1}}}{\varepsilon ^2}\sum _{i, k\in \mathbb {N}}k\left( \theta (k,\delta ,i)\right) ^{1/4}. \end{aligned}$$

Combining (4.17) and (4.18) we obtain

$$\begin{aligned} \mathbb {P}(\mu _n\notin \overline{K^{\delta }})&\le \frac{\delta }{4}+C_0(\overline{B}_0,T)|\mathbb {T}^3|\sum _{k\in \mathbb {N}} \frac{k}{R^2(k,\delta )}\\  &\quad +\frac{C_3(\overline{B}_0,T)\left\| h\right\| _{W^{2,1}}}{\varepsilon ^2}\sum _{i, k\in \mathbb {N}}k\left( \theta (k,\delta ,i)\right) ^{1/4}. \end{aligned}$$

Choosing

$$\begin{aligned} R(k,\delta )=\sqrt{\frac{2\pi ^3k^3 C_0(\overline{B}_0,T)|\mathbb {T}^3|}{3\delta }},\quad \theta (k,\delta ,i)=\left( \frac{3\varepsilon ^2}{2^{i+1}\pi ^2k^3\left\| h\right\| _{W^{2,1}}C_3(\overline{B}_0,T)}\right) ^4 \end{aligned}$$

the claim follows and the proof is complete. □

### Passage to the limit

Thanks to Theorem 4.6, by Jakubowski version of Skorokhod’s representation’s theorem, [25, Theorem 2], up to passing to subsequences, we can find an auxiliary probability space, that for simplicity we continue to call $(\Omega ,\mathcal {F},\mathbb {P})$, and processes

$$\begin{aligned} \left( \tilde{\mu }_n,\ W^n:=\{W^{n,k,j}\}_{\begin{array}{c} k\in \mathbb {Z}^3_0\\ j\in \{1,2\} \end{array}}\right) ,\quad \left( \overline{\mu },\ W:=\{W^{k,j}\}_{\begin{array}{c} k\in \mathbb {Z}^3_0\\ j\in \{1,2\} \end{array}}\right) , \end{aligned}$$

such that

$$\begin{aligned}&\tilde{\mu }_n\rightarrow \overline{\mu } \quad \text {in } C([0,T];\mathcal {Y}) \quad \mathbb {P}-a.s.\\&W^n\rightarrow W \quad \text {in } C([0,T];\mathbb {R}^{\mathbb {Z}^3_0\times \mathbb {Z}^3_0} )\quad \mathbb {P}-a.s. \end{aligned}$$

Of course the convergence above between $W^n$ and *W* can be seen as the uniform convergence of cylindrical Wiener processes $$W^n=\sum _{\begin{array}{c} k\in \mathbb {Z}^{3}_0\\ j\in \{1,2\} \end{array}}e_{k,j} W^{n,k,j},\ W=\sum _{\begin{array}{c} k\in \mathbb {Z}^{3}_0\\ j\in \{1,2\} \end{array}}e_{k,j} W^{k,j}$$ on a suitable Hilbert space $\mathcal {U}_0$. Before going on, in order to identify the PDE satisfied by $\overline{\mu }$ we provide integrability properties of $\overline{\mu }$.

#### Proposition 4.7

The limit measure $\overline{\mu }$ satisfies

$$\begin{aligned} \mathbb {E} \left[ \sup _{t\in [0,T]}\int _{\mathbb {T}^3\times \mathbb {R}^3} |b|^2 \overline{\mu }(t,x,db)dx\right] \le C(\overline{B}_0,T). \end{aligned}$$

#### Proof

Since $\tilde{\mu }_n$ converges to $\overline{\mu }$ in $C([0,T];\mathcal {Y})$, for each $f\in C_{b}(\mathbb {T}^3\times \mathbb {R}^3)$ and p≥1, by dominated convergence theorem,

4.19 $$\begin{aligned} \int _0^T\left\lvert \int _{\mathbb {T}^3\times \mathbb {R}^3} f(b,x) \tilde{\mu }_n(t,x,db)dx-\int _{\mathbb {T}^3\times \mathbb {R}^3} f(b,x) \overline{\mu }(t,x,db)dx\right\rvert ^p dt\rightarrow 0 \quad \mathbb {P}-a.s. \end{aligned}$$

Choose a smooth test function such that

$$\begin{aligned} \phi ^M(b,x)={\left\{ \begin{array}{ll} |b|^2\quad & \text {if } |b|\le M\\ 0\quad & \text {if } |b|\ge M+1, \end{array}\right. }\quad \left\| \phi ^M\right\| _{C_b}\le M^2. \end{aligned}$$

Obviously $\phi ^M(b,x)\nearrow |b|^2$ pointwise. Therefore, due to (4.19), Fatou’s Lemma and Theorem 2.10

$$\begin{aligned}&\mathbb {E} \left[ \left( \int _0^T\left\lvert \int _{\mathbb {T}^3\times \mathbb {R}^3} \phi ^M(b,x) \overline{\mu }(t,x,db)dx\right\rvert ^p dt\right) ^{1/p}\right] \\&\quad \le \liminf _{n\rightarrow +\infty } \mathbb {E} \left[ \left( \int _0^T\left\lvert \int _{\mathbb {T}^3\times \mathbb {R}^3} \phi ^M(b,x) \tilde{\mu }_n(t,x,db)dx\right\rvert ^p dt\right) ^{1/p}\right] \\&\quad \le \liminf _{n\rightarrow +\infty } \mathbb {E} \left[ \sup _{t\in [0,T]} \int _{\mathbb {T}^3\times \mathbb {R}^3} |b|^2 \tilde{\mu }_n(t,x,db)dx\right] \\&\quad \le C_0(\overline{B}_0,T). \end{aligned}$$

Then, by monotone convergence theorem, for each p≥1

4.20 $$\begin{aligned} \mathbb {E} \left[ \left( \int _0^T\left\lvert \int _{\mathbb {T}^3\times \mathbb {R}^3} |b|^2 \overline{\mu }(t,x,db)dx\right\rvert ^p dt\right) ^{1/p}\right]&\le C_0(\overline{B}_0,T). \end{aligned}$$

This implies that $\left\| \int _{\mathbb {T}^3\times \mathbb {R}^3} |b|^2 \overline{\mu }(\cdot , x,db)dx\right\| _{L^p(0,T)}<+\infty \quad \mathbb {P}-a.s.$ Therefore

4.21 $$\begin{aligned} \left\| \int _{\mathbb {T}^3\times \mathbb {R}^3} |b|^2 \overline{\mu }(\cdot ,x,db)dx\right\| _{L^p(0,T)}\rightarrow \left\| \int _{\mathbb {T}^3\times \mathbb {R}^3} |b|^2 \overline{\mu }(\cdot , x,db)dx\right\| _{L^\infty (0,T)}. \end{aligned}$$

Combining (4.20) and (4.21) we obtain the result by Fatou’s lemma and the continuity of $\overline{\mu }$. □

By the properties of Riemann sums we have

4.22 $$\begin{aligned}&\mathcal {L}^n(b)=(b\otimes b):\left( \sum _{\begin{array}{c} k\in \mathbb {Z}^3_0\\ n\le |k|\le 2n \end{array}}\frac{k\otimes k}{|k|^5}\otimes \left( I-\frac{k\otimes k}{|k|^2}\right) \right) \nonumber \\  &\rightarrow \mathcal {L}(b):=(b\otimes b):\int _{\xi \in \mathbb {R}^3,\ 1\le |\xi |\le 2} \frac{\xi \otimes \xi }{|\xi |^5}\otimes \left( I-\frac{\xi \otimes \xi }{|\xi |^2}\right) d\xi \nonumber \\  &=\frac{8\pi \log 2}{15}\left( 2|b|^2 I-b\otimes b\right) \end{aligned}$$

uniformly on compact sets.

Let us introduce our limit PDE on the space of Young measures

4.23 $$\begin{aligned} {\left\{ \begin{array}{ll} \partial _t \mu (t,x,db)& ={{\,\textrm{div}\,}}(\mathcal {L}(b)\mu (t,x,db))\quad b\in \mathbb {R}^3,x\in \mathbb {T}^3\ t\in [0,T]\\ \mu (0,x,db)& =\delta _{\overline{B}_0(x)}(db) \end{array}\right. } \end{aligned}$$

and its notion of solution.

#### Definition 4.8

We say that $\mu \in C([0,T];\mathcal {Y})$ is a weak solution of equation (4.23) if

$$\begin{aligned} \mu (0,x,db)=\delta _{\overline{B}_0(x)}(db),\quad \int _0^T \int _{\mathbb {T}^3\times \mathbb {R}^3}|b|^2 \mu (t,x,db)dx<+\infty \end{aligned}$$

and for each $f\in C_c^1((0,T);C^2_c(\mathbb {T}^3\times \mathbb {R}^3))$ it holds

$$\begin{aligned}&\int _0^T\int _{\mathbb {T}^3\times \mathbb {R}^3}\partial _sf(s,x,b){\mu }(s,x,db)dxds \\  &\quad +\int _0^T\int _{\mathbb {T}^3\times \mathbb {R}^3} {{\,\textrm{div}\,}}_b\left( \mathcal {L}(b)\nabla _b f_s(x,b)\right) {\mu }(s,x,db) dx ds=0. \end{aligned}$$

#### Remark 4.9

Arguing as in [34, Remark 2.3], if μ is a weak solution of (4.23) in the sense of Definition 4.8, then

$$\begin{aligned}&\int _{\mathbb {T}^3\times \mathbb {R}^3} f_{t_2}(x,b)\mu (t_2,x,db)dx- \int _{\mathbb {T}^3\times \mathbb {R}^3} f_{t_1}(x,b)\mu (t_1,x,db)dx \\  &\quad =\int _{t_1}^{t_2}\int _{\mathbb {T}^3\times \mathbb {R}^3}\partial _sf(s,x,b){\mu }(s,x,db)dxds\\  &\qquad +\int _{t_1}^{t_2}\int _{\mathbb {T}^3\times \mathbb {R}^3} {{\,\textrm{div}\,}}_b\left( \mathcal {L}(b)\nabla _b f_s(x,b)\right) \mu (s,x,db) dx ds \end{aligned}$$

for each $0\le t_1\le t_2\le T$ and $f\in C^1_b\left( [0,T];C^2_b(\mathbb {T}^3\times \mathbb {R}^3)\right)$.

The uniqueness of solutions of (4.23) in the sense of Definition 4.8 is an easy application of the results of [34]. Indeed, the following holds.

#### Proposition 4.10

There exists a unique $\mu \in C([0,T];\mathcal {Y})$ which solves (4.23) in the sense of Definition 4.8.

#### Proof

We start observing that for each $b\in \mathbb {R}^3$

$$\begin{aligned} \mathcal {L}(b)=\frac{8\pi \log 2 |b|^2}{15} \frac{b\otimes b}{|b|^2}+\frac{16\pi \log 2 |b|^2}{15} \left( I-\frac{b\otimes b }{|b|^2}\right) . \end{aligned}$$

Therefore $$ \mathcal {L}(b)=\mathcal {A}(b)\mathcal {A}(b)$$, where

$$\begin{aligned} \mathcal {A}(b)=\frac{\sqrt{8\pi \log 2} |b|}{\sqrt{15}} \frac{b\otimes b}{|b|^2}+\frac{4\sqrt{\pi \log 2 }|b|}{\sqrt{15}} \left( I-\frac{b\otimes b }{|b|^2}\right) . \end{aligned}$$

In particular $\mathcal {A}(b)$ is a symmetric, non negative matrix for each $b\in \mathbb {R}^3$ and the function $b\rightarrow \mathcal {A}(b)$ is Lipschitz with linear growth. Since for each $j\in \{1,2,3\},\ \sum _{i=1}^3 \partial _i\mathcal {L}_{i,j}(b)=0$, if $\hat{W}$ is a 3D Brownian motion, then the stochastic differential equation

4.24 $$\begin{aligned} {\left\{ \begin{array}{ll} dX_t& =0,\\ db_t& =\mathcal {A}(b_t)d\hat{W}_t\\ X_0& =x\\ b_0& =b \end{array}\right. } \end{aligned}$$

has (4.23) as Fokker-Planck equation associated. We have uniqueness in law of weak solutions of the stochastic differential equation (4.24) due to the properties of $\mathcal {A}(b)$. The latter implies uniqueness of solutions of (4.23) due to [34, Theorem 2.5]. □

Therefore, we are left to show that our limit object $\overline{\mu }$ is a weak solution of (4.23). This is the content of the following result.

#### Proposition 4.11

$\overline{\mu }$ is the unique weak solution of (4.23) in the sense of Definition 4.8.

#### Proof

We divide the proof in two steps.

*Step 1: test function independent of time*. Let us start showing that for each $f\in C^2_c(\mathbb {T}^3\times \mathbb {R}^3)$ and t∈[0,T]

4.25 $$\begin{aligned} \begin{array}{c} \int _{\mathbb {T}^3\times \mathbb {R}^3}f(x,b)(\overline{\mu }(t,x,db)-\delta _{\overline{B}_0(x)}(db)) dx\\ = \int _0^t\int _{\mathbb {T}^3\times \mathbb {R}^3} {{\,\textrm{div}\,}}_b\left( \mathcal {L}(b)\nabla _b f(x,b)\right) \overline{\mu }(s,x,db) dx ds \quad \mathbb {P}-a.s. \end{array}\end{aligned}$$

By standard arguments, see for example [19, Chapter 2], also $\tilde{\mu }_n$ satisfies (4.2). Now we pass to the limit in each term of (4.2). Thanks to the a.s. convergence of $\tilde{\mu }_n$ to $\overline{\mu }$ in $C([0,T];\mathcal {Y})$ we obtain

$$\begin{aligned}&\int _{\mathbb {T}^3\times \mathbb {R}^3}f(x,b)(\tilde{\mu }_n(t,x,db)-\delta _{\overline{B}_0(x)}(db)) dx\rightarrow \\  &\int _{\mathbb {T}^3\times \mathbb {R}^3}f(x,b)(\overline{\mu }(t,x,db)-\delta _{\overline{B}_0(x)}(db)) dx\quad \mathbb {P}-a.s. \end{aligned}$$

Secondly, as discussed at the beginning of the proof of Proposition 4.7, since $\eta _n\rightarrow 0$, $\mathcal {L}^n(b)~\rightarrow ~\mathcal {L}(b)$ uniformly on compact sets and *f* has compact support we have

$$\begin{aligned} \left\lvert \eta _n\int _0^t\int _{\mathbb {T}^3\times \mathbb {R}^3} \Delta _x f(x,b)\tilde{\mu }_n(s,x,db) dx ds\right\rvert \lesssim \eta _n \left\| f\right\| _{C^2_b}~\rightarrow 0, \end{aligned}$$

$$\begin{aligned}&\left\lvert \int _0^t\int _{\mathbb {T}^3\times \mathbb {R}^3} {{\,\textrm{div}\,}}_b\left( \mathcal {L}^n(b)\nabla _b f(x,b)\right) \tilde{\mu }_n(s,x,db) dx ds\right. \\  &\left. -\int _0^t\int _{\mathbb {T}^3\times \mathbb {R}^3} {{\,\textrm{div}\,}}_b\left( \mathcal {L}(b)\nabla _b f(x,b)\right) \overline{\mu }(s,x,db) dx ds\right\rvert \nonumber \\  &\le \left\lvert \int _0^t\int _{\mathbb {T}^3\times \mathbb {R}^3} {{\,\textrm{div}\,}}_b\left( \left( \mathcal {L}^n(b)-\mathcal {L}(b)\right) \nabla _b f(x,b)\right) \tilde{\mu }_n(s,x,db) dx ds\right\rvert \nonumber \\  &+\left\lvert \int _0^t\int _{\mathbb {T}^3\times \mathbb {R}^3} {{\,\textrm{div}\,}}_b\left( \mathcal {L}(b)\nabla _b f(x,b)\right) \left( \overline{\mu }(s,x,db)-\tilde{\mu }_n(s,x,db)\right) dx ds\right\rvert \\&\rightarrow 0\quad \mathbb {P}-a.s., \end{aligned}$$

where we used that ${{\,\textrm{div}\,}}_b\left( \mathcal {L}^n(b)-\mathcal {L}(b)\right) =0$ to show the convergence of the first summand. Concerning the stochastic integrals we argue by Burkholder-Davis-Gundy inequality obtaining

$$\begin{aligned}&\mathbb {E} \left[ |\sum _{\begin{array}{c} k\in \mathbb {Z}^{3}_0\\ j\in \{1,2\} \end{array}}\int _0^t\int _{\mathbb {T}^3\times \mathbb {R}^3} \sigma _{k,j}^{n}(x)\cdot (\nabla _x f)(x,b)\tilde{\mu }_n(s,x,db)dxdW^{k,j}_s|^2\right] \\&=\sum _{\begin{array}{c} k\in \mathbb {Z}^{3}_0\\ j\in \{1,2\} \end{array}}\mathbb {E} \left[ \int _0^t|\int _{\mathbb {T}^3\times \mathbb {R}^3} \sigma _{k,j}^{n}(x)\cdot (\nabla _x f)(x,b)\tilde{\mu }_n(s,x,db)dx |^2 ds\right] \\  &\lesssim \sum _{\begin{array}{c} k\in \mathbb {Z}^3_0\\ n\le |k|\le 2n \end{array} } \frac{1}{|k|^5}\left\| \nabla _x f\right\| _{C_b}^2\rightarrow 0. \end{aligned}$$

For the second one we need to argue in a more precise way, using the fact that $$\{a_{k,j}\otimes a_{k,l} e^{ik\cdot x}\}_{\begin{array}{c} k\in \mathbb {Z}^3_0\\ j,l\in \{1,2,3\} \end{array}}$$ is an orthonormal basis of $L^2(\mathbb {T}^3;\mathbb {R}^3\otimes \mathbb {R}^3)$. Let us define for each *f* and *n* the process

$$\begin{aligned} F^n_f(t,x)=\int _{\mathbb {R}^3}b\otimes \nabla _b f(x,b) \tilde{\mu }_n(t,x,db). \end{aligned}$$

Due to Jensen’s inequality and Theorem 2.10

$$\begin{aligned} \mathbb {E} \left[ \sup _{t\in [0,T]}\left\| F^n_f(t)\right\| _{L^2(\mathbb {T}^3)}^2\right]&\le \mathbb {E} \left[ \sup _{t\in [0,T]}\int _{\mathbb {T}^3}\int _{\mathbb {R}^3}|b\otimes \nabla _b f(x,b) |^2\tilde{\mu }_n(t,x,db) dx \right] \\  &\le \left\| f\right\| _{C^1_b}^2\mathbb {E} \left[ \sup _{t\in [0,T]}\int _{\mathbb {T}^3}\int _{\mathbb {R}^3}|b|^2\tilde{\mu }_n(t,x,db) dx \right] \\  &\le \left\| f\right\| _{C^1_b}^2 C_0(\overline{B}_0,T). \end{aligned}$$

Therefore

$$\begin{aligned}  &   \mathbb {E} \left[ \left\lvert \sum _{\begin{array}{c} k\in \mathbb {Z}^{3}_0\\ j\in \{1,2\} \end{array}}\int _0^t\int _{\mathbb {T}^3\times \mathbb {R}^3} b\cdot \nabla \sigma _{k,j}^{n}(x)\nabla _b f(x,b)\tilde{\mu }_n(s,x,db)dxdW^{k,j}_s \right\rvert ^2\right] \\    &   \quad =\sum _{\begin{array}{c} k\in \mathbb {Z}^{3}_0\\ j\in \{1,2\} \end{array}}\mathbb {E} \left[ \int _0^t\left\lvert \int _{\mathbb {T}^3\times \mathbb {R}^3} b\cdot \nabla \sigma _{k,j}^{n}(x)\nabla _b f(x,b)\tilde{\mu }_n(s,x,db)dx\right\rvert ^2 ds\right] \\    &   \quad = \sum _{\begin{array}{c} k\in \mathbb {Z}^{3}_0\\ j\in \{1,2\} \end{array}}|\theta _{k,j}^{n}|^2|k|^2\mathbb {E} \left[ \int _0^t \left\langle \frac{k}{|k|}\otimes a_{k,j} e^{ik\cdot x}, F^n_f(s,x) \right\rangle _{L^2(\mathbb {T}^3)}^2 ds\right] \\    &   \quad \le \frac{1}{n^3}\mathbb {E} \left[ \int _0^T \left\| F^n_f(t)\right\| _{L^2(\mathbb {T}^3)}^2 dt \right] \\    &   \quad \le \frac{T}{n^3}\left\| f\right\| _{C^1_b}^2 C_0(\overline{B}_0,T)\rightarrow 0. \end{aligned}$$

This completes the proof of the first step.

*Step 2: Conclusion.* By continuity in time of the objects and separability of $C^2_c(\mathbb {T}^3\times \mathbb {R}^3)$ we can find a zero probability set $\mathcal {N}$ such that on its complementary the PDE in weak form is satisfied for each t∈[0,T], $f\in C^{2}_c(\mathbb {T}^3\times \mathbb {R}^3)$. Secondly, let us consider $f\in C^{1}_c((0,T);C^2_c(\mathbb {T}^3\times \mathbb {R}^3))$ and $0=t_0<t_1<\dots <t_N=T$ a partition of [0, *T*] also denoted by π. Thanks to the identity

$$\begin{aligned}\begin{array}{c} \int _{\mathbb {T}^3\times \mathbb {R}^3} f_{t_{i+1}}(x,b)\overline{\mu }(t_{i+1},x,db)dx-\int _{\mathbb {T}^3\times \mathbb {R}^3} f_{t_{i}}(x,b)\overline{\mu }(t_{i+1},x,db)dx\\ =\int _{t_{i}}^{t_{i+1}}\int _{\mathbb {T}^3\times \mathbb {R}^3} \partial _s f_{s}(x,b)\overline{\mu }(t_{i+1},x,db)dxds \end{array}\end{aligned}$$

and relation (4.25) we obtain

$$\begin{aligned} \int _{\mathbb {T}^3\times \mathbb {R}^3}f_{i+1}(x,b)\overline{\mu }(t_{i+1},x,db)dx&-\int _{\mathbb {T}^3\times \mathbb {R}^3}f_{t_i}(x,b)\overline{\mu }(t_{i},x,db)dx\\  &= \int _{t_{i}}^{t_{i+1}}\int _{\mathbb {T}^3\times \mathbb {R}^3} \partial _s f_{s}(x,b)\overline{\mu }(t_{i+1},x,db)dxds\\  &\quad +\int _{t_{i}}^{t_{i+1}}\int _{\mathbb {T}^3\times \mathbb {R}^3} {{\,\textrm{div}\,}}_b\left( \mathcal {L}(b)\nabla _b f_{t_i}(x,b)\right) \overline{\mu }(s,x,db) dx ds, \end{aligned}$$

which implies

$$\begin{aligned}&\int _{0}^{T}\int _{\mathbb {T}^3\times \mathbb {R}^3} \partial _s f_{s}(x,b)\overline{\mu }(s^{+}_{\pi },x,db)dxds\\  &\quad +\int _{0}^{T}\int _{\mathbb {T}^3\times \mathbb {R}^3} {{\,\textrm{div}\,}}_b\left( \mathcal {L}(b)\nabla _b f_{s^{-}_{\pi }}(x,b)\right) \overline{\mu }(s,x,db) dx ds=0, \end{aligned}$$

where $s^{-}_{\pi }=t_i$ and $s^{+}_{\pi }=t_{i+1}$ if $s\in [t_i,t_{i+1})$. Taking a sequence $\pi _n$ with size going to 0 we conclude the proof thanks to dominated convergence’s theorem and the regularity of $\overline{\mu }$ and *f*. □

Combining Theorem 4.6, Proposition 4.10 and Proposition 4.11 we can easily prove Theorem 2.11.

#### Proof of Theorem 2.11

We denote by $\mathscr {L}_n$ the law of $\mu _n$ on $C([0,T];\mathcal {Y})$. By Theorem 4.6, Proposition 4.10 and Proposition 4.11, every subsequence $\mathscr {L}_{n(k)}$ admits a sub-subsequence which converges to the unique limit point $\delta _\mu$, where μ is the deterministic solution of (4.23) in the sense of Definition 4.8. Then, for example by [7, Theorem 2.6], the whole sequence $\mathscr {L}_{n}$ converges weakly to $\delta _\mu$, and then also in probability as the limit point is a Dirac delta (see e.g. the argument in [7, page 27]). The last claim follows introducing a family of functions $f^{M,i}\in C^2_b(\mathbb {T}^3\times \mathbb {R}^3)$ for $i\in \{1,2,3\},\ M\in \mathbb {N}$ such that

$$\begin{aligned} \left\| \nabla ^2 f^{M,i}\right\| _{C_b(\mathbb {T}^3\times \mathbb {R}^3)}\le 1,\quad |f^{M,i}(x,b)|\le |b|,\quad f^{M,i}(x,b)= b^i \quad&\text {if } |b |\le M. \end{aligned}$$

Using these $f^{M,i}$ as test functions in (4.25) and letting $M\rightarrow +\infty$, by dominated convergence theorem we get the claim. The proof is complete. □

## Data Availability

Data sharing not applicable to this article as no data sets were generated or analyzed during the current study.

## Global well-posedness of equation (2.8)

The proof of Theorem 2.10 is a consequence of a vanishing viscosity procedure. In order to ease the notation, let us fix χ=1. For each $n\in \mathbb {N}$, given a family of positive numbers $\eta \rightarrow 0$, let us introduce the approximate problem

A.1 $$\begin{aligned} {\left\{ \begin{array}{ll} dB^{n,\eta }_t& =(\frac{2}{3}\eta _n+\eta )\Delta B^{n,\eta }_t dt+\sum _{\begin{array}{c} k\in \mathbb {Z}^{3}_0\\ j\in \{1,2\} \end{array}}(\sigma _{k,j}^{n}\cdot \nabla B^{n,\eta }_t-B^{n,\eta }_t\cdot \nabla \sigma _{k,j}^{n})dW^{k,j}_t,\\ {{\,\textrm{div}\,}}B^{n,\eta }_t& =0,\\ B^{n,\eta }_0& =\overline{B}_0. \end{array}\right. } \end{aligned}$$

Analogously to Definition 2.9 we introduced a notion of weak solution for the viscous problem (A.1).

### Definition A.1

A stochastic process $C_{\mathcal {F}}(0,T;V)\cap L_{\mathcal {F}}^2(0,T;\textbf{H}^2)$ is a weak solution of equation (A.1) if $\mathbb {P}-$a.s.

$$\begin{aligned} \langle B^{n,\eta }_t,\phi \rangle -\langle \overline{B}_0,\phi \rangle&=-\left( \frac{2}{3}\eta _n+\eta \right) \int _0^t \langle \nabla B^{n,\eta }_s,\nabla \phi \rangle ds\\  &+\sum _{\begin{array}{c} k\in \mathbb {Z}^{3}_0\\ j\in \{1,2\} \end{array}}\int _0^t \langle \sigma _{k,j}^{n}\cdot \nabla B^{n,\eta }_s - B^{n,\eta }_s\cdot \nabla \sigma _{k,j}^{n},\phi \rangle dW^{k,j}_s \end{aligned}$$

for each $$\phi \in V,\ t\in [0,T]$$.

The well-posedness of the stochastic PDE above is a well-known fact, see for example [16, Chapters 3-5]. Indeed the following holds

### Theorem A.2

For each $\overline{B}_0\in V$ there exists a unique weak solution of (A.1) in the sense of Definition A.1.

### A priori estimates for the viscous equation

In this section we provide for each $n\in \mathbb {N}$ some a priori estimates for $B^{n,\eta }$ uniformly in the viscosity parameter.

#### Proposition A.3

For each $\gamma \ge 4$ we have

$$\begin{aligned} \mathbb {E} \left[ \sup _{t\in [0,T]}\left\| B^{n,\eta }_t\right\| _V^\gamma \right] \lesssim _{n,\overline{B}_0,T,\gamma }1. \end{aligned}$$

#### Proof

We divide the proof of this proposition in many steps.

*Step 1: Itô formula.* By Definition A.1

$$\begin{aligned} \int _0^T \left\| \Delta B^{n,\eta }_t\right\| ^2 dt<+\infty \quad \mathbb {P}-a.s. \end{aligned}$$

and

$$\begin{aligned} \sum _{\begin{array}{c} k\in \mathbb {Z}^{3}_0\\ j\in \{1,2\} \end{array}}\int _0^t \sigma _{k,j}^{n}\cdot \nabla B^{n,\eta }_s-B^{n,\eta }_s\cdot \nabla \sigma _{k,j}^{n}dW^{k,j}_s \end{aligned}$$

is a *V* valued local martingale. Therefore we can apply Itô formula in *V*, see [30, Theorem 2.13], obtaining

$$\begin{aligned} d\left\| \nabla B^{n,\eta }_t\right\| ^2+2\left( \frac{2}{3}\eta _n+\eta \right) \left\| \Delta B^{n,\eta }_t\right\| ^2dt&=dM^{n,\eta }_t+I^{n,\eta }_tdt, \end{aligned}$$

where

$$\begin{aligned} dM^{n,\eta }_t&:=2\sum _{\begin{array}{c} k\in \mathbb {Z}^{3}_0\\ j\in \{1,2\} \end{array}}\langle \nabla (\sigma _{k,j}^{n}\cdot \nabla B^{n,\eta }_t-B^{n,\eta }_t\cdot \nabla \sigma _{k,j}^{n}),\nabla B^{n,\eta }_t\rangle dW^{k,j}_t,\\ I^{n,\eta }_t&:=2\sum _{\begin{array}{c} k\in \mathbb {Z}^{3}_0\\ j\in \{1,2\} \end{array}}\langle \nabla (\sigma _{k,j}^{n}\cdot \nabla B^{n,\eta }_t-B^{n,\eta }_t\cdot \nabla \sigma _{k,j}^{n}), \nabla (\sigma _{-k,j}^{n}\cdot \nabla B^{n,\eta }_t-B^{n,\eta }_t\cdot \nabla \sigma _{-k,j}^{n}) \rangle . \end{aligned}$$

Since $\sigma _{k,j}^{n}$ are divergence free the second order term in $B^{n,\eta }$ cancels, i.e.

$$\begin{aligned} \langle \sigma _{k,j}^{n}\cdot \nabla ^2 B^{n,\eta }_t,\nabla B^{n,\eta }_t\rangle = 0. \end{aligned}$$

Therefore

$$\begin{aligned} dM^{n,\eta }_t=2\sum _{\begin{array}{c} k\in \mathbb {Z}^{3}_0\\ j\in \{1,2\} \end{array}}\langle \nabla \sigma _{k,j}^{n}\cdot \nabla B^{n,\eta }_t-\nabla B^{n,\eta }_t\cdot \nabla \sigma _{k,j}^{n}-B^{n,\eta }_t\cdot \nabla ^2\sigma _{k,j}^{n},\nabla B^{n,\eta }_t\rangle dW^{k,j}_t. \end{aligned}$$

Let us write better also $I^{n,\eta }_t$

$$\begin{aligned} I^{n,\eta }_t&=I^{0,n,\eta }_t+I^{1,n,\eta }_t+I^{2,n,\eta }_t+I^{3,n,\eta }_t+I^{4,n,\eta }_t, \end{aligned}$$

where

$$\begin{aligned} I^{0,n,\eta }_t&=2\sum _{\begin{array}{c} k\in \mathbb {Z}^{3}_0\\ j\in \{1,2\} \end{array}}\langle \nabla \sigma _{k,j}^{n}\cdot \nabla B^{n,\eta }_t, \nabla \sigma _{-k,j}^{n}\cdot \nabla B^{n,\eta }_t-\nabla B^{n,\eta }_t\cdot \nabla \sigma _{-k,j}^{n}\rangle \\&-2\sum _{\begin{array}{c} k\in \mathbb {Z}^{3}_0\\ j\in \{1,2\} \end{array}}\langle \nabla B^{n,\eta }_t\cdot \nabla \sigma _{k,j}^{n},\nabla \sigma _{-k,j}^{n}\cdot \nabla B^{n,\eta }_t-\nabla B^{n,\eta }_t\cdot \nabla \sigma _{-k,j}^{n}\rangle \\  &+2\sum _{\begin{array}{c} k\in \mathbb {Z}^{3}_0\\ j\in \{1,2\} \end{array}}\langle B^{n,\eta }_t\cdot \nabla ^2 \sigma _{k,j}^{n},B^{n,\eta }_t\cdot \nabla ^2 \sigma _{-k,j}^{n}\rangle ,\\ I^{1,n,\eta }_t&=-2\sum _{\begin{array}{c} k\in \mathbb {Z}^{3}_0\\ j\in \{1,2\} \end{array}}\left( \langle \sigma _{k,j}^{n}\cdot \nabla ^2 B^{n,\eta }_t ,B^{n,\eta }_t\cdot \nabla \sigma _{-k,j}^{n}\rangle +\langle B^{n,\eta }_t\cdot \nabla \sigma _{k,j}^{n},\sigma _{-k,j}^{n}\cdot \nabla ^2 B^{n,\eta }_t \rangle \right) ,\\ I^{2,n,\eta }_t&=2\sum _{\begin{array}{c} k\in \mathbb {Z}^{3}_0\\ j\in \{1,2\} \end{array}}\langle \sigma _{k,j}^{n}\cdot \nabla ^2 B^{n,\eta }_t,\nabla \sigma _{-k,j}^{n}\cdot \nabla B^{n,\eta }_t-\nabla B^{n,\eta }_t\cdot \nabla \sigma _{-k,j}^{n}\rangle \\  &+2\sum _{\begin{array}{c} k\in \mathbb {Z}^{3}_0\\ j\in \{1,2\} \end{array}}\langle \nabla \sigma _{k,j}^{n}\cdot \nabla B^{n,\eta }_t-\nabla B^{n,\eta }_t\cdot \nabla \sigma _{k,j}^{n},\sigma _{-k,j}^{n}\cdot \nabla ^2 B^{n,\eta }_t\rangle ,\\ I^{3,n,\eta }_t&=-2\sum _{\begin{array}{c} k\in \mathbb {Z}^{3}_0\\ j\in \{1,2\} \end{array}}\langle \nabla \sigma _{k,j}^{n}\cdot \nabla B^{n,\eta }_t-\nabla B^{n,\eta }_t\cdot \nabla \sigma _{k,j}^{n}, B^{n,\eta }_t\cdot \nabla ^2\sigma _{-k,j}^{n}\rangle \\  &-2\sum _{\begin{array}{c} k\in \mathbb {Z}^{3}_0\\ j\in \{1,2\} \end{array}}\langle \nabla \sigma _{-k,j}^{n}\cdot \nabla B^{n,\eta }_t-\nabla B^{n,\eta }_t\cdot \nabla \sigma _{-k,j}^{n}, B^{n,\eta }_t\cdot \nabla ^2\sigma _{k,j}^{n}\rangle , \\ I^{4,n,\eta }_t&=2\sum _{\begin{array}{c} k\in \mathbb {Z}^{3}_0\\ j\in \{1,2\} \end{array}}\langle \sigma _{k,j}^{n}\cdot \nabla ^2 B^{n,\eta }_t, \sigma _{-k,j}^{n}\cdot \nabla ^2 B^{n,\eta }_t \rangle . \end{aligned}$$

Now we show that $I^{1,n,\eta }_t=I^{2,n,\eta }_t=I^{3,n,\eta }_t=0,\ I^{4,n,\eta }_t=\frac{4}{3}\left\| \Delta B^{n,\eta }_t\right\| ^2$. Expanding the definition of each summand in $I^{1,n,\eta }_t$ we have

$$\begin{aligned}&\langle \sigma _{k,j}^{n}\cdot \nabla ^2 B^{n,\eta }_t ,B^{n,\eta }_t\cdot \nabla \sigma _{-k,j}^{n}\rangle +\langle B^{n,\eta }_t\cdot \nabla \sigma _{k,j}^{n},\sigma _{-k,j}^{n}\cdot \nabla ^2 B^{n,\eta }_t, \rangle \\&\quad =-i|\theta _{k,j}^{n}|^2\sum _{\alpha ,\beta ,\gamma ,\theta =1}^3\int _{\mathbb {T}^3} (a_{k,j})_{\alpha }\partial _{\alpha ,\beta }(B^{n,\eta }_t)_{\gamma }(B^{n,\eta }_t)_{\theta }(k)_{\theta }(a_{k,j})_{\gamma } dx\\&\qquad +i|\theta _{k,j}^{n}|^2\sum _{\alpha ,\beta ,\gamma ,\theta =1}^3\int _{\mathbb {T}^3} (a_{k,j})_{\alpha }\partial _{\alpha ,\beta }(B^{n,\eta }_t)_{\gamma }(B^{n,\eta }_t)_{\theta }(k)_{\theta }(a_{k,j})_{\gamma } dx=0. \end{aligned}$$

The analysis of $I^{2,n,\eta }_t,\ I^{3,n,\eta }_t$ is analogous and we omit the detailed computations. Concerning $I^{4,n,\eta }_t$, we have, recalling that $$\sum _{\begin{array}{c} k\in \mathbb {Z}^{3}_0\\ j\in \{1,2\} \end{array}}|\theta _{k,j}^{n}|^2 (a_{k,j})_{\alpha }(a_{k,j})_{\beta }=\frac{2}{3}\eta _n\delta _{\alpha ,\beta }$$,

$$\begin{aligned} I^{4,n,\eta }_t&=\frac{4}{3}\eta _n \sum _{\alpha ,\beta ,\gamma =1}^3 \langle \partial _{\alpha ,\beta } (B^{n,\eta }_t)_{\gamma },\partial _{\alpha ,\beta } (B^{n,\eta }_t)_{\gamma }\rangle \\  &=\frac{4}{3}\eta _n \left\| \Delta B^{n,\eta }_t\right\| ^2. \end{aligned}$$

In conclusion, so far we showed that

A.2 $$\begin{aligned} d\left\| \nabla B^{n,\eta }_t\right\| ^2+2\eta \left\| \Delta B^{n,\eta }_t\right\| ^2dt&=dM^{n,\eta }_t+I^{0,n,\eta }_tdt. \end{aligned}$$

Therefore, applying finite dimensional Itô formula to relation (A.2) we obtain for $\gamma \ge 4$

A.3 $$\begin{aligned} d&\left\| \nabla B^{n,\eta }_t\right\| ^\gamma +\eta \gamma \left\| \nabla B^{n,\eta }_t\right\| ^{\gamma -2}\left\| \Delta B^{n,\eta }_t\right\| ^2dt \nonumber \\  &=\frac{\gamma }{2}\left\| \nabla B^{n,\eta }_t\right\| ^{\gamma -2} I^{0,n,\eta }_t dt+\frac{\gamma }{2}\left\| \nabla B^{n,\eta }_t\right\| ^{\gamma -2}dM^{n,\eta }_t+\gamma (\gamma -2)\left\| \nabla B^{n,\eta }_t\right\| ^{\gamma -4}\nonumber \\&\times \sum _{\begin{array}{c} k\in \mathbb {Z}^{3}_0\\ j\in \{1,2\} \end{array}}\left\lvert \langle \nabla \sigma _{k,j}^{n}\cdot \nabla B^{n,\eta }_t-\nabla B^{n,\eta }_t\cdot \nabla \sigma _{k,j}^{n}-B^{n,\eta }_t\cdot \nabla ^2\sigma _{k,j}^{n},\nabla B^{n,\eta }_t\rangle \right\rvert ^2 dt. \end{aligned}$$

*Step 2: Proof of the estimates.* The reason why expressions (A.2) and (A.3) allow us to obtain estimates independent of the viscosity is that second-order derivatives of $B^{n,\eta }_t$ do not appear in any term on the right-hand side. For each $M\ge 1,\ n,\ \eta$ let $\tau _M=\inf \{t\in [0,T]:\ \left\| \nabla B^{n,\eta }_t\right\| ^2\ge M\}\wedge T$. Integrating (A.2) between 0 and $r\le \tau ^M$ and taking the expected value of the supremum in time for $r\le t\wedge \tau _M$ we obtain, by Burkholder-Davis-Gundy inequality and Hölder’s inequality, since only finite $\sigma _{k,j}^{n}$ are different from 0

$$\begin{aligned}&\mathbb {E} \left[ \sup _{r\le t}\left\| \nabla B^{n,\eta }_{r \wedge \tau _M}\right\| ^2\right] =\mathbb {E} \left[ \sup _{r\le t\wedge \tau _M}\left\| \nabla B^{n,\eta }_r\right\| ^2\right] \\&\quad \le \left\| \nabla \overline{B}_0\right\| ^2+\mathbb {E} \left[ \int _0^{t\wedge \tau _M}|I^{0,n,\eta }_s|ds\right] +\mathbb {E} \left[ \left( \int _0^{t\wedge \tau _M}d[M^{n,\eta },M^{n,\eta }]_s ds\right) ^{1/2}\right] \\&\quad \le \left\| \nabla \overline{B}_0\right\| ^2+C_{n}\mathbb {E} \left[ \int _0^{t\wedge \tau _M}\left\| \nabla B^{n,\eta }_s\right\| ^2 ds\right] \\&\quad \quad \quad \qquad +C_{n}\mathbb {E} \left[ \left( \int _0^{t\wedge \tau _M}\left\| \nabla B^{n,\eta }_s\right\| ^4 ds\right) ^{1/2}\right] \\&\quad \le \left\| \nabla \overline{B}_0\right\| ^2+C_{n}\mathbb {E} \left[ \int _0^{t\wedge \tau _M}\left\| \nabla B^{n,\eta }_s\right\| ^2 ds\right] \\&\quad \quad \quad +C_{n}\mathbb {E} \left[ \sup _{r\le t\wedge \tau _M}\left\| \nabla B^{n,\eta }_r\right\| \left( \int _0^{t\wedge \tau _M}\left\| \nabla B^{n,\eta }_s\right\| ^2 ds\right) ^{1/2}\right] \\&\quad \le \left\| \nabla \overline{B}_0\right\| ^2+\frac{1}{2}\mathbb {E} \left[ \sup _{r\le t\wedge \tau _M}\left\| \nabla B^{n,\eta }_r\right\| ^2\right] +C_{n}\mathbb {E} \left[ \int _0^{t\wedge \tau _M}\left\| \nabla B^{n,\eta }_s\right\| ^2 ds\right] , \end{aligned}$$

where the last step follows by Young’s inequality. In conclusion, by simple manipulations, we proved that

$$\begin{aligned} \mathbb {E} \left[ \sup _{r\le t}\left\| \nabla B^{n,\eta }_{r \wedge \tau _M}\right\| ^2\right] \le 2\left\| \nabla \overline{B}_0\right\| ^2+C_n\mathbb {E} \left[ \int _0^t \sup _{r\le s} \left\| \nabla B^{n,\eta }_{r \wedge \tau _M}\right\| ^2 ds\right] . \end{aligned}$$

By Grönwall’s lemma, the latter implies

$$\begin{aligned} \mathbb {E} \left[ \sup _{r\le T\wedge \tau _M}\left\| \nabla B^{n,\eta }_r\right\| ^2\right] =\mathbb {E} \left[ \sup _{r\le T}\left\| \nabla B^{n,\eta }_{r \wedge \tau _M}\right\| ^2\right] \lesssim _{n,\overline{B}_0,T}1. \end{aligned}$$

Since $\tau _M\rightarrow T$ as $M\rightarrow +\infty$ due to the fact that $B^{n,\eta }\in C([0,T];V)$, letting $M\rightarrow +\infty$ the latter implies the claim for γ=2 by monotone convergence. The proof of the claim for $\gamma \ge 4$ is analogous, only starting by (A.3) in place of (A.2) and we omit the easy details. □

#### Proposition A.4

For each $\beta \ge 4,s_1\in (0,\frac{1}{2}),r_1>2$ such that $r_1s_1>1$ we have

$$\begin{aligned} \mathbb {E} \left[ \int _0^T\int _0^T \frac{\left\| B^{n,\eta }_t-B^{n,\eta }_s\right\| _{\textbf{H}^{-\beta }}^{r_1}}{|t-s|^{1+r_1s_1}}dt ds\right] \lesssim _{n,\overline{B}_0,\beta ,r_1,s_1,T} 1. \end{aligned}$$

#### Proof

We start estimating $\mathbb {E} \left[ |\langle B^{n,\eta }_t-B^{n,\eta }_s,a_{h,l}e^{ih\cdot x}\rangle |^{r_1}\right]$ for each $h\in \mathbb {Z}^3_0,\ l\in \{1,2\}$. Thanks to the weak formulation satisfied by $B^{n,\eta }_t$ we have

$$\begin{aligned} \langle B^{n,\eta }_t-B^{n,\eta }_s,a_{h,l}e^{ih\cdot x}\rangle&=\left( \frac{2}{3}\eta _n+\eta \right) \int _s^t \langle \nabla B^{n,\eta }_r,h\otimes a_{h,l}e^{ih\cdot x}\rangle dr\\  &+\sum _{\begin{array}{c} k\in \mathbb {Z}^{3}_0\\ j\in \{1,2\} \end{array}}\int _s^t \langle \sigma _{k,j}^{n}\cdot \nabla B^{n,\eta }_r - B^{n,\eta }_r\cdot \nabla \sigma _{k,j}^{n},a_{h,l}e^{ih\cdot x}\rangle dW^{k,j}_r. \end{aligned}$$

Taking the modulus of the expression above and raising to the power $r_1$ we can estimate the quantity required by Burkholder-Davis-Gundy inequality. Indeed, thanks to Hölder’s inequality and Proposition A.3

A.4 $$\begin{aligned}&\mathbb {E} \left[ |\langle B^{n,\eta }_t-B^{n,\eta }_s,a_{h,l}e^{ih\cdot x}\rangle |^{r_1}\right] \nonumber \\&\quad \lesssim _{r_1,n}|h|^r\mathbb {E} \left[ \left( \int _s^t\left\| \nabla B^{n,\eta }_r\right\| dr\right) ^{r_1}\right] +\mathbb {E} \left[ \left( \int _s^t \left\| \nabla B^{n,\eta }_r\right\| ^{2}dr\right) ^{r_1/2}\right] \nonumber \\  &\quad \le |h|^{r_1}|t-s|^{r_1}\mathbb {E} \left[ \sup _{r\in [0,T]}\left\| \nabla B^{n,\varepsilon }_r\right\| ^{r_1}\right] +|t-s|^{\frac{r_1}{2}}\mathbb {E} \left[ \sup _{r\in [0,T]}\left\| \nabla B^{n,\varepsilon }_r\right\| ^{r_1}\right] \nonumber \\  &\quad \lesssim _{n,\overline{B}_0,T,r_1} |h|^{r_1}|t-s|^{\frac{r_1}{2}}. \end{aligned}$$

Secondly, by definition of $\textbf{H}^{-\beta }$ norm and Hölder’s inequality for some ε>0 small enough

A.5 $$\begin{aligned} \mathbb {E} \left[ \left\| B^{n,\eta }_t-B^{n,\eta }_s\right\| ^{r_1}_{\textbf{H}^{-\beta }}\right]&=\mathbb {E} \left[ \left( \sum _{\begin{array}{c} h\in \mathbb {Z}^3_0\\ l\in \{1,2\} \end{array}}\frac{\langle B^{n,\eta }_t-B^{n,\eta }_s,a_{h,l}e^{ih\cdot x} \rangle ^2}{|h|^{2\beta }} \right) ^{\frac{r_1}{2}}\right] \nonumber \\  &=\mathbb {E} \left[ \left( \sum _{\begin{array}{c} h\in \mathbb {Z}^3_0\\ l\in \{1,2\} \end{array}}\frac{\langle B^{n,\eta }_t-B^{n,\eta }_s,a_{h,l}e^{ih\cdot x} \rangle ^2}{|h|^{\frac{3(1+\varepsilon )(r_1-2)}{r_1}}|h|^{\frac{2\beta r_1-3(1+\varepsilon )(r_1-2)}{r_1}}} \right) ^{\frac{r_1}{2}}\right] \nonumber \\  &\le \left( \sum _{\begin{array}{c} h\in \mathbb {Z}^3_0\\ l\in \{1,2\} \end{array}}\frac{1}{|h|^{3(1+\varepsilon )}}\right) ^{\frac{r_1-2}{2}} \mathbb {E} \left[ \sum _{\begin{array}{c} h\in \mathbb {Z}^3_0\\ l\in \{1,2\} \end{array}}\frac{\langle B^{n,\eta }_t-B^{n,\eta }_s,a_{h,l}e^{ih\cdot x} \rangle ^{r_1}}{|h|^{\beta r_1-\frac{3(1+\varepsilon )(r_1-2)}{2}}} \right] \nonumber \\  &\lesssim _{r_1}\mathbb {E} \left[ \sum _{\begin{array}{c} h\in \mathbb {Z}^3_0\\ l\in \{1,2\} \end{array}}\frac{\langle B^{n,\eta }_t-B^{n,\eta }_s,a_{h,l}e^{ih\cdot x} \rangle ^{r_1}}{|h|^{\beta r_1 -\frac{3(1+\varepsilon )(r_1-2)}{2}}} \right] . \end{aligned}$$

Combining (A.4) and (A.5) we get immediately the claim of the proposition. Indeed

$$\begin{aligned}\begin{array}{c} \mathbb {E} \left[ \int _0^T\int _0^T \frac{\left\| B^{n,\eta }_t-B^{n,\eta }_s\right\| _{\textbf{H}^{-\beta }}^{r_1}}{|t-s|^{1+r_1s_1}}dt ds\right] \\ \lesssim _{n,\overline{B}_0,T,r_1}\sum _{\begin{array}{c} h\in \mathbb {Z}^3_0\\ l\in \{1,2\} \end{array}}\frac{1}{|h|^{(\beta -1)r_1-\frac{3(1+\varepsilon )(r_1-2)}{2}}}\int _0^T\int _0^T\frac{1}{|t-s|^{1+r_1(s_1-\frac{1}{2})}}dtds. \end{array}\end{aligned}$$

The latter being finite as soon as $s_1\in (0,\frac{1}{2})$ and

$$\begin{aligned} (\beta -1)r_1-\frac{3(1+\varepsilon )(r_1-2)}{2}>3. \end{aligned}$$

The relation above is always satisfied if $$\beta \ge 4$$ for each choice of $$r_1>2 $$ and $$\varepsilon \in (0,1)$$. □

### Tightness and limit object

Let us denote by

$$\begin{aligned} \mathbb {B}_R=\{x\in V: \left\| x\right\| _V\le R\} \end{aligned}$$

and *q* the metric on $\mathbb {B}_R$ compatible with the weak topology. Let us introduce the space

$$\begin{aligned} C([0,T];\mathbb {B}_R)=\{f\in C([0,T];V_w):\ \left\| f(t)\right\| _V\le R\quad \forall t\in [0,T]\}. \end{aligned}$$

The space $C([0,T];\mathbb {B}_R)$ is metrizable and complete if endowed with the metric

$$\begin{aligned} d(u,v)=\sup _{t\in [0,T]}q(u(t),v(t)) \end{aligned}$$

see [8–10]. Secondly we denote by

$$\begin{aligned} Z_T=C([0,T];H)\cap C([0,T];V_w). \end{aligned}$$

endowed with the supremum of the corresponding topologies, i.e. the coarsest topology on $Z_T$ that is finer either than the one in *C*([0, *T*]; *H*) and the one in $C([0,T];V_w).$ We are interested to introduce compact sets in $Z_T$. For this reason we recall the following results, see [11, Lemma 2.1] and [31, Corollary 5].

#### Theorem A.5

Let $u_k\in C([0,T];V_w)$ such that

$$\begin{aligned} \sup _k \sup _{t\in [0,T]}\left\| u_k(t)\right\| _V\le R,\quad u_k\rightarrow u\quad \text {in } C([0,T];H). \end{aligned}$$

Then $$u_k,u \in C([0,T];\mathbb {B}_r),\ u_k\rightarrow u $$ in $$C([0,T];\mathbb {B}_r)$$.

#### Theorem A.6

Let $p,r \in [1,+\infty ]$ and $s\in \mathbb {R}$; assume that s>0 if r≥p or s>1/r-1/p if r≤p. Let $X,\ B,\ Y$ be separable Banach spaces such that

$$\begin{aligned} X{\mathop {\hookrightarrow }\limits ^{c}}B\hookrightarrow Y; \end{aligned}$$

and *F* a bounded subset in $L^p(0,T;X)\cap W^{s,r}(0,T;Y)$. Then *F* is relatively compact in $L^p(0,T;B)$ (in *C*([0, *T*]; *B*) if p=+∞).

Combining Theorem A.5 and Theorem A.6 we obtain the following.

#### Lemma A.7

Let $s\in (0,\frac{1}{2}),\ r>2$ such that sr>1 and *F* a bounded subset in $C(0,T;V)\cap W^{s,r}(0,T;\textbf{H}^{-4})$. Then *F* is relatively compact in $Z_T$.

#### Proof

Since *F* is bounded in *C*([0, *T*]; *V*),  there exists R<+∞ such that

$$\begin{aligned} \sup _{f\in F}\left\| f\right\| _{C([0,T];V)}\le R. \end{aligned}$$

Therefore we can consider on $Z_T$ the metric of $C([0,T];\mathbb {B}_R)\cap C([0,T];H)$. In particular compactness is equivalent to sequentially compactness. Let $(f_k)\in F$. Thanks to Theorem A.6 with $p=+\infty ,\ X=V,\ B=H,\ Y=\textbf{H}^{-4}$ there exists a subsequence $k_j$ and f∈C([0,T];H) such that

$$\begin{aligned} f_{k_j}\rightarrow f\quad \text {in }C([0,T];H). \end{aligned}$$

Then Theorem A.5 implies that also

$$\begin{aligned} f_{k_j}\rightarrow f\quad \text {in }C([0,T];\mathbb {B}_R). \end{aligned}$$

This completes the proof. □

Having Proposition A.3, Proposition A.4, Lemma A.7 in mind we get immediately the following:

#### Corollary A.8

For each $n\in \mathbb {N}$ the family of laws $\{\mathscr {L}(B^{n,\eta })\}_{\eta > 0}$ is tight in $Z_T$.

By standard arguments, see for example [19, Chapter 2] or [9, Section 5.3], by Jakubowski version of Skorokhod’s representation’s theorem, [25, Theorem 2], for each $n\in \mathbb {N}$ we can find, up to passing to subsequences, an auxiliary probability space, that for simplicity we continue to call $(\Omega ,\mathcal {F},\mathbb {P})$, and processes $(\tilde{B}^{n,\eta _h},W^{h}:=\{W^{k,j,h}\}_{k\in \mathbb {Z}^3_0,\ j\in \{1,2\}}),\ (B^n,W:=\{W^{k,j}\}_{k\in \mathbb {Z}^3_0,\ j\in \{1,2\}}),$ such that

$$\begin{aligned}&\tilde{B}^{n,\eta _h}\rightarrow B^n\quad \text {in } C([0,T];V_w)\cap C([0,T];H) \quad \mathbb {P}-a.s.\\&W^h\rightarrow W \quad \text {in } C([0,T];\mathbb {C}^{\mathbb {Z}^3_0\times \mathbb {Z}^3_0})\quad \mathbb {P}-a.s. \end{aligned}$$

Of course the convergence above between $W^n$ and *W* can be seen as the uniform convergence of cylindrical Wiener processes

$$\begin{aligned} W^h=\sum _{\begin{array}{c} k\in \mathbb {Z}^{3}_0\\ j\in \{1,2\} \end{array}}a_{k,j}e^{ik\cdot x} W^{k,j,h},\ W=\sum _{\begin{array}{c} k\in \mathbb {Z}^{3}_0\\ j\in \{1,2\} \end{array}}a_{k,j}e^{ik\cdot x} W^{k,j} \end{aligned}$$

on a suitable Hilbert space $\mathcal {U}_0$. Before going on, in order to identify $B^n$ as a weak solution of equation (2.8) we need further integrability properties of $B^n$.

#### Proposition A.9

For each $$\gamma \ge 4$$

$$\begin{aligned} \mathbb {E} \left[ \sup _{t\in [0,T]}\left\| B^{n}_t\right\| _V^\gamma \right] \lesssim _{n,\overline{B}_0,T,\gamma }1. \end{aligned}$$

#### Proof

Since $\tilde{B}^{n,\eta _h}$ has the same law of $B^{n,\eta _h}$, by Proposition A.3 for each $\gamma \ge 4$

$$\begin{aligned} \sup _{h\in \mathbb {N}}\mathbb {E} \left[ \sup _{t\in [0,T]}\left\| \nabla \tilde{B}^{n,\eta _h}\right\| ^\gamma \right] \lesssim _{n,\overline{B}_0,T,\gamma }1. \end{aligned}$$

In particular up to passing to a further subsequence $B^{n,\eta ,h_l}$ converges weakly star in $L^{\gamma }(\Omega ;L^{\infty }(0,T;V))$ to some $\overline{B}^n$. The latter implies also that the weak star convergence holds in $L^{\gamma }(\Omega ;L^{\infty }(0,T;H))$. Since $B^{n,\eta ,h_l}\rightarrow B^n\ \mathbb {P}-a.s.$ in $C([0,T];H)\hookrightarrow L^{\infty }(0,T;H)$ we have that $B^n=\overline{B}^n$ and in particular the claim. □

Now we are in the position to prove Theorem 2.10.

#### Proof of Theorem 2.10

We divide the proof in some steps.

*Step 1: A priori estimates.* We start showing that each solution of (2.8) in the sense of Definition 2.9 satisfies (2.9), (2.10), (2.11), (2.12). By Definition 2.9

$$\begin{aligned} \int _0^T \left\| \Delta B^{n}_t\right\| _{\textbf{H}^{-1}}^2 dt<+\infty \quad \mathbb {P}-a.s. \end{aligned}$$

and

$$\begin{aligned} \sum _{\begin{array}{c} k\in \mathbb {Z}^{3}_0\\ j\in \{1,2\} \end{array}}\int _0^t \sigma _{k,j}^{n}\cdot \nabla B^{n}_s-B^{n}_s\cdot \nabla \sigma _{k,j}^{n}dW^{k,j}_s \end{aligned}$$

is a *H* valued local martingale. Therefore we can apply Itô formula in *H*, see [30, Theorem 2.13], obtaining (2.9). Secondly, applying finite dimensional Itô formula to relation (2.9) with $f(x)=|x|^{\gamma /2}$ for $\gamma \ge 4$ we obtain (2.10). For each $M\ge 1,\ n\in \mathbb {N}$ let $\tau _M=\inf \{t\in [0,T]:\ \left\| \nabla B^{n}_t\right\| ^2\ge M\}\wedge T$. Integrating (2.9) between 0 and $r\le \tau ^M$ and taking the expected value of the supremum in time for $r\le t\wedge \tau _M$ we obtain, by Burkholder-Davis-Gundy inequality and Hölder’s inequality

$$\begin{aligned} \mathbb {E} \left[ \sup _{r\le t}\left\| B^{n}_{r \wedge \tau _M}\right\| ^2\right]&=\mathbb {E} \left[ \sup _{r\le t\wedge \tau _M}\left\| B^{n}_r\right\| ^2\right] \\  &\le \left\| \overline{B}_0\right\| ^2+\sum _{\begin{array}{c} k\in \mathbb {Z}^{3}_0\\ j\in \{1,2\} \end{array}}|k\theta _{k,j}^{n}|^2\mathbb {E} \left[ \int _0^{t\wedge \tau _M}\Vert B^n_s\Vert ^2 ds\right] \\  &\quad +\mathbb {E} \left[ \left( \int _0^{t\wedge \tau _M}\sum _{\begin{array}{c} k\in \mathbb {Z}^{3}_0\\ j\in \{1,2\} \end{array}}|k\theta _{k,j}^{n}|^2 \Vert B^n_s\Vert ^4 ds\right) ^{1/2}\right] \\  &\le \left\| \overline{B}_0\right\| ^2\\&\quad +C\mathbb {E} \left[ \int _0^{t\wedge \tau _M}\left\| B^{n}_s\right\| ^2 ds\right] +C\mathbb {E} \left[ \left( \int _0^{t\wedge \tau _M}\left\| B^{n}_s\right\| ^4 ds\right) ^{1/2}\right] \\  &\le \left\| \overline{B}_0\right\| ^2+C\mathbb {E} \left[ \int _0^{t\wedge \tau _M}\left\| B^{n}_s\right\| ^2 ds\right] \\  &\quad \quad \quad \quad \qquad +C\mathbb {E} \left[ \sup _{r\le t\wedge \tau _M}\left\| B^{n}_r\right\| \left( \int _0^{t\wedge \tau _M}\left\| B^{n}_s\right\| ^2 ds\right) ^{1/2}\right] \\  &\le \left\| \overline{B}_0\right\| ^2+\frac{1}{2}\mathbb {E} \left[ \sup _{r\le t\wedge \tau _M}\left\| B^{n}_r\right\| ^2\right] +C\mathbb {E} \left[ \int _0^{t\wedge \tau _M}\left\| B^{n}_s\right\| ^2 ds\right] , \end{aligned}$$

where in the second step we exploited the fact that

$$\begin{aligned} \sum _{\begin{array}{c} k\in \mathbb {Z}^{3}_0\\ j\in \{1,2\} \end{array}}|k\theta _{k,j}^{n}|^2=\sum _{\begin{array}{c} k\in \mathbb {Z}^3_0\\ n\le |k|\le 2n\\ j\in \{1,2\} \end{array}}\frac{1}{|k|^3}\lesssim 1 \end{aligned}$$

and in the last one we applied Young’s inequality. In conclusion, by simple manipulations, we proved that

A.6 $$\begin{aligned} \mathbb {E} \left[ \sup _{r\le t}\left\| B^{n}_{r \wedge \tau _M}\right\| ^2\right] \le 2\mathbb {E} \left[ \left\| \overline{B}_0\right\| ^2\right] +C\mathbb {E} \left[ \int _0^t \sup _{r\le s} \left\| B^{n}_{r \wedge \tau _M}\right\| ^2 ds\right] . \end{aligned}$$

By Grönwall’s lemma, the latter implies

$$\begin{aligned} \mathbb {E} \left[ \sup _{r\le T\wedge \tau _M}\left\| B^{n}_r\right\| ^2\right] =\mathbb {E} \left[ \sup _{r\le T}\left\| B^{n}_{r \wedge \tau _M}\right\| ^2\right] \lesssim _{\overline{B}_0,T}1. \end{aligned}$$

Since $\tau _M\rightarrow T$ as $M\rightarrow +\infty$ due to the fact that $B^{n}\in C([0,T];H)$, letting $M\rightarrow +\infty$ the latter implies equation (2.11) by monotone convergence. The proof of equation (2.12) is analogous, only starting by (2.10) in place of (2.9) and we omit the easy details.

*Step 2: Uniqueness.* By linearity of the equation it is enough to prove uniqueness in case of $\overline{B}_0=0$. In particular, due (A.6), our solution starting from $\overline{B}_0=0$ satisfies

$$\begin{aligned} \mathbb {E} \left[ \sup _{r\le t}\left\| B^{n}_{r \wedge \tau _M}\right\| ^2\right] \le C\mathbb {E} \left[ \int _0^t \sup _{r\le s} \left\| B^{n}_{r \wedge \tau _M}\right\| ^2 ds\right] . \end{aligned}$$

By Grönwall’s lemma, the latter implies

$$\begin{aligned} \mathbb {E} \left[ \sup _{r\le T\wedge \tau _M}\left\| B^{n}_r\right\| ^2\right] =\mathbb {E} \left[ \sup _{r\le T}\left\| B^{n}_{r \wedge \tau _M}\right\| ^2\right] =0. \end{aligned}$$

Since $\tau _M\rightarrow T$ as $M\rightarrow +\infty$ due to the fact that $B^{n}\in C([0,T];H)$, letting $M\rightarrow +\infty$ the $B^n\equiv 0$ and the uniqueness follows.

*Step 3: Limit equation.* Let $\phi \in \textbf{H}^2$ we know that

$$\begin{aligned} \langle \tilde{B}^{n,\eta _h}_t,\phi \rangle -\langle \overline{B}_0,\phi \rangle&=\left( \frac{2}{3}\eta _n+\eta _{h}\right) \int _0^t \langle \tilde{B}^{n,\eta _h}_s,\Delta \phi \rangle ds\\  &\quad +\sum _{\begin{array}{c} k\in \mathbb {Z}^{3}_0\\ j\in \{1,2\} \end{array}}\int _0^t\langle \sigma _{k,j}^{n}\times B^{n,\eta _h}_s,\nabla \times \phi \rangle dW^{k,j,h}_s\quad \mathbb {P}-a.s. \end{aligned}$$

Therefore we will show, up to passing to a further subsequence, $\mathbb {P}$-a.s. convergence of all the terms appearing above, uniformly in time. Thanks to the fact that $\tilde{B}^{n,\eta _h}\rightarrow B^n\ \mathbb {P}-a.s.$ in *C*([0, *T*]; *H*) we get easily

$$\begin{aligned}&\sup _{t\in [0,T]}|\langle \tilde{B}^{n,\eta _h}_t-B^n_t,\phi \rangle |\rightarrow 0\quad \mathbb {P}-a.s.,\\&\sup _{t\in [0,T]}\left\lvert \left( \frac{2}{3}\eta _n+\eta _{h}\right) \int _0^t \langle \tilde{B}^{n,\eta _h}_s,\Delta \phi \rangle ds- \frac{2}{3}\eta _n \int _0^t \langle \tilde{B}^{n}_s,\Delta \phi \rangle ds\right\rvert \rightarrow 0\quad \mathbb {P}-a.s. \end{aligned}$$

We are left to show the convergence of the stochastic integrals. Since

$$\begin{aligned}&\sum _{\begin{array}{c} k\in \mathbb {Z}^{3}_0\\ j\in \{1,2\} \end{array}}\int _0^T\langle \sigma _{k,j}^{n}\times \left( B^{n,\eta _h}_s-B^n_s\right) ,\nabla \times \phi \rangle ^2 ds\\  &\le \sum _{\begin{array}{c} k\in \mathbb {Z}^3_0\\ n\le |k|\le 2n\\ j\in \{1,2\} \end{array}}\frac{1}{|k|^5}\left\| \phi \right\| _V^2 T\left\| B^{n,\eta _h}-B^n\right\| _{C([0,T];H)}^2\\  &\rightarrow 0, \end{aligned}$$

we can apply [5, Lemma 4.3] obtaining that

$$\begin{aligned}&\sup _{t\in [0,T]}\left\lvert \sum _{\begin{array}{c} k\in \mathbb {Z}^{3}_0\\ j\in \{1,2\} \end{array}}\int _0^t\langle \sigma _{k,j}^{n}\times B^{n,\eta _h}_s,\nabla \times \phi \rangle dW^{k,j,h}_s - \int _0^t\langle \sigma _{k,j}^{n}\times B^{n}_s,\nabla \times \phi \rangle dW^{k,j}_s\right\rvert \\&\quad \rightarrow 0 \quad \text {in probability}, \end{aligned}$$

therefore $\mathbb {P}-$a.s. up to passing to a further sub-subsequence. In conclusion, so far we showed that for each $\phi \in \textbf{H}^2$

A.7 $$\begin{aligned} \langle B^n_t,\phi \rangle -\langle \overline{B}_0,\phi \rangle&=\frac{2}{3}\eta _n\int _0^t \langle B^n_s,\Delta \phi \rangle ds+\sum _{\begin{array}{c} k\in \mathbb {Z}^{3}_0\\ j\in \{1,2\} \end{array}}\int _0^t \langle \sigma _{k,j}^{n}\times B^n_s ,\nabla \times \phi \rangle dW^{k,j}_s \nonumber \\  &\quad \mathbb {P}-a.s.\quad \forall t\in [0,T]. \end{aligned}$$

By standard density argument, since $B^n\in C([0,T];V_w)$, we can find a zero measure set $\mathcal {N}\subset \Omega$ such that on its complementary relation (A.7) holds for each $\phi \in V$. We omit the easy details.

*Step 4: Conclusion.* Every subsequence $\mathscr {L}(B^{n,\eta _h})$ admits a sub-subsequence which converges to the unique limit point $\mathscr {L}(B^n)$, where $B^n$ is the unique weak solution of (2.8) in the sense of Definition 2.9. Then, the Gyongy–Krylov Convergence Criterion, see [24], applies and the whole sequence $B^{n,\eta _h}$ converges in probability in $C([0,T];H)\cap L^2(0,T;V)$ to $B^n$ in the original probability space. The proof is complete. □

## Some remarks on the occupation measure

Let $f:\mathbb {T}^{3}\rightarrow \mathbb {R}^{3}$ be a measurable function. Given $x_{0}\in \mathbb {T}^{3}$ and ε>0, call $\nu _{x_{0},\varepsilon }$ the probability measure on Borel sets of $\mathbb {R}^{3}$ defined as

$$ \int _{\mathbb {R}^{k}}\varphi \left( y\right) \nu _{x_{0},\varepsilon }\left( dy\right) =\frac{1}{\left| U_{\varepsilon }\left( x_{0}\right) \right| }\int _{U_{\varepsilon }\left( x_{0}\right) }\varphi \left( f\left( x\right) \right) dx $$

for every $\varphi \in C_{b}\left( \mathbb {R}^{3}\right)$, where $U_{\varepsilon }\left( x_{0}\right) \subset \mathbb {T}^3$ is the ball in $\mathbb {T}^{3}$ of center $x_{0}$ and radius ε and $\left| U_{\varepsilon }\left( x_{0}\right) \right|$ is its Lebesgue measure. In other words, $\nu _{x_{0},\varepsilon }$ is the push forward of the normalized Lebesgue measure $\frac{1}{\left| U_{\varepsilon }\left( x_{0}\right) \right| }\mathcal {L}_{3}|_{U_{\varepsilon }\left( x_{0}\right) }$ on $U_{\varepsilon }\left( x_{0}\right)$ under the map *f*; or the image law of the random variable *f* considered on the probability space $\left( U_{\varepsilon }\left( x_{0}\right) ,\mathcal {B}\left( U_{\varepsilon }\left( x_{0}\right) \right) ,\frac{1}{\left| U_{\varepsilon }\left( x_{0}\right) \right| }\mathcal {L}_3|_{U_{\varepsilon }\left( x_{0}\right) }\right)$. *We call *$\nu _{x_{0},\varepsilon }$* the local occupation measure around *$x_{0}$* of size *ε*. *

Clearly, if *f* is continuous, the weak limit as $\varepsilon \rightarrow 0$ of $\nu _{x_{0},\varepsilon }$ is $\delta _{f\left( x_{0}\right) }$. When *f* is rapidly oscillating, however, $\nu _{x_{0},\varepsilon }$ *captures the local oscillations in a statistical sense*, for finite ε. Here “statistical” is understood in a spatial sense (opposite to the more common temporal or probabilistic sense): where (on $\mathbb {R}^{3}$) $\nu _{x_{0},\varepsilon }$ gives more mass, more often we observe such values of *f* around $x_{0}$. The notion becomes particularly natural from the view point of interpretation in the stationary case, notion that we express here only in a loose way: if the phenomenon at hand is independent of position $x_{0}$ and maybe of a time parameter, $\nu _{x_{0},\varepsilon }$ captures the oscillations of *f* in a statistical sense, where now “statistical” may be interpreted in a wider sense, not only locally spatial.

Rigorously, assume we have a sequence of measurable functions $\left( f_{n}\right)$ from $\mathbb {T}^{3}$ to $\mathbb {R}^{3}$ and a measurable function $x\mapsto \nu _{x}$ from $\mathbb {T}^{3}$ to the space of probability measures on Borel sets of $\mathbb {R}^{3}$ endowed with the topology of weak convergence, such that

$$ \lim _{n\rightarrow \infty }\int _{\mathbb {T}^{3}}\varphi \left( f_{n}\left( x\right) \right) \psi \left( x\right) dx=\int _{\mathbb {T}^{3}}\left( \int _{\mathbb {R}^{3}}\varphi \left( y\right) \nu _{x}\left( dy\right) \right) \psi \left( x\right) dx $$

for every $\varphi \in C_{b}\left( \mathbb {R}^{3}\right)$ and $\psi \in C_{b}\left( \mathbb {T}^{3}\right)$. *The measure-valued function *$\left( \nu _{x}\right)$* is called the Young measure associated with the sequence *$\left( f_{n}\right)$. The above convergence extends to $\psi \in L^{2}\left( \mathbb {T}^{3}\right)$ by an easy argument of density, taking into account that the sequence $\left\| \varphi \left( f_{n}\left( \cdot \right) \right) \right\| _{\infty }$ is bounded. Then we can take $\psi =\frac{1}{\left| U_{\varepsilon }\left( x_{0}\right) \right| }\boldsymbol{1}_{U_{\varepsilon }\left( x_{0}\right) }$ and get

$$ \lim _{n\rightarrow \infty }\frac{1}{\left| U_{\varepsilon }\left( x_{0}\right) \right| }\int _{U_{\varepsilon }\left( x_{0}\right) }\varphi \left( f_{n}\left( x\right) \right) dx=\frac{1}{\left| U_{\varepsilon }\left( x_{0}\right) \right| }\int _{U_{\varepsilon }\left( x_{0}\right) }\left( \int _{\mathbb {R}^{3}}\varphi \left( y\right) \nu _{x}\left( dy\right) \right) dx. $$

In other words, if $\nu _{x_{0},\varepsilon }^{n}$ is the local occupation measure of $f_{n}$, we have

$$ \lim _{n\rightarrow \infty }\int _{\mathbb {R}^{3}}\varphi \left( y\right) \nu _{x_{0},\varepsilon }^{n}\left( dy\right) =\frac{1}{\left| U_{\varepsilon }\left( x_{0}\right) \right| }\int _{U_{\varepsilon }\left( x_{0}\right) }\left( \int _{\mathbb {R}^{3}}\varphi \left( y\right) \nu _{x}\left( dy\right) \right) dx. $$

More expressively, we may write

$$ \lim _{n\rightarrow \infty }\nu _{x_{0},\varepsilon }^{n}=\frac{1}{\left| U_{\varepsilon }\left( x_{0}\right) \right| }\int _{U_{\varepsilon }\left( x_{0}\right) } \nu _{x}dx $$

in the weak sense of measures. This formula provides an interpretation for the Young measure $\left( \nu _{x}\right)$ of the sequence $\left( f_{n}\right)$: *its local average captures the oscillations of *$f_{n}$* in a statistical sense, in the limit as *$n\rightarrow \infty$. In the homogeneous cases, when $\nu _{x}$ is independent of *x*, the interpretation is particularly strong.

### Large values

In the case of a scalar transport equation, the values of solutions for positive time cannot exceed the values of the initial conditions.

In the case of a vector-advection equation, the stretching term may increase the length of the vectors. The physical phenomenon of magnetic dynamo is an example. The theory developed above may help to recognize the existence of large values of the length of the vectors. Let us see a quantitative statement.

Assume we have

B.1 $$\begin{aligned} \lim _{n\rightarrow \infty }\int _{\mathbb {T}^{3}}\varphi \left( B_t^{n}\left( x\right) \right) \psi \left( x\right) dx=\int _{\mathbb {T}^{3}}\left( \int _{\mathbb {R}^{3}}\varphi \left( b\right) \mu _{t,x}\left( db\right) \right) \psi \left( x\right) dx \end{aligned}$$

for a sequence of vector fields $\left( B^{n}_t\right)$, where $\varphi ,\psi ,\mu _{t,x}$ are as above. Denote by $\mathcal {L}_{3}$ the Lebesgue measure on $\mathbb {T}^{3}$.

#### Proposition B.1

Assume that there exists a ball $U_{r}\left( x_{0}\right) \subset \mathbb {T}^{3}$ and real numbers R,λ>0 such that

$$ \mu _{t,x}\left( U_{R}\left( 0\right) ^{c}\right) \ge \lambda \qquad \text {for all }x\in U_{r}\left( x_{0}\right) , $$

where above $U_R\left( 0\right) \subset \mathbb {R}^3$ denotes the ball in $\mathbb {R}^3$ centered in 0 with radius *R*. Then, for all $\lambda ^{\prime }\in \left( 0,\lambda \right)$ there exists $n_{0}\in \mathbb {N}$ such that for all $n\ge n_{0}$

$$ \mathcal {L}_{3}\left\{ x\in U_r(x_0) :\left| B_t^{n}\left( x\right) \right| \ge R\right\} \ge \lambda ^{\prime }\mathcal {L} _{3}\left( U_r(x_0) \right) . $$

#### Proof

For every $\delta \in \left( 0,\frac{R}{2}\right)$, let $\varphi _{\delta }$ be a smooth function on $\mathbb {R}^{3}$, equal to 1 on $U_{R-\delta }\left( 0\right) ^{c}$, zero on $U_{R-2\delta }\left( 0\right)$, with values in $\left[ 0,1\right]$ in $U_{R-\delta }\left( 0\right) \backslash U_{R-2\delta }\left( 0\right)$; and similarly let $\psi _{\delta }$ be a smooth function on $\mathbb {T}^{d}$, equal to 1 on $U_{r+\delta }\left( x_{0}\right)$, zero on $U_{r+2\delta }\left( x_{0}\right)$, with values in $\left[ 0,1\right]$ in $U_{r+2\delta }\left( x_{0}\right) \backslash U_{r+\delta }\left( x_{0}\right)$. Then

$$ \int _{\mathbb {R}^{3}}\varphi _{\delta }\left( b\right) \mu _{t,x}\left( db\right) \ge \lambda \qquad \text {for all }x\in U_r\left( x_{0}\right) $$

and thus

$$ \int _{\mathbb {T}^{3}}\left( \int _{\mathbb {R}^{3}}\varphi _{\delta }\left( b\right) \nu _{t,x}\left( db\right) \right) \psi _{\delta }\left( x\right) dx\ge \lambda \mathcal {L}_{3}\left( U_r\left( x_{0}\right) \right) . $$

Therefore, given $\lambda ^{\prime }\in \left( 0,\lambda \right)$, there exists $n_{0}\in \mathbb {N}$ such that for all $n\ge n_{0}$

$$ \int _{\mathbb {T}^{3}}\varphi _{\delta }\left( B_t^{n}\left( x\right) \right) \psi _{\delta }\left( x\right) dx\ge \lambda ^{\prime }\mathcal {L}_{3}\left( U_r\left( x_{0}\right) \right) . $$

We may easily deduce, by the arbitrariness of δ, that

$$ \int _{U_r\left( x_{0}\right) }\boldsymbol{1}_{\left\{ \left| B_t^{n}\left( x\right) \right| \ge R\right\} }dx\ge \lambda ^{\prime }\mathcal {L}_{3}\left( U_r\left( x_{0}\right) \right) . $$

This is the claimed formula. □

Due to Theorem 2.11, relation (B.1) is satisfied $\mathbb {P}-a.s.$ up to passing to subsequences. To be able to use Proposition B.1 to say something about large values attained by the stochastic PDE for $B^n_t$, we prove here also that for every $x \in \mathbb {T}^3$ such that $\overline{B}_0(x)\ne 0$, the limit measure μ(t,x,db) has full support on $\mathbb {R}^3$ for every t>0. This fact does not follow trivially by a standard application of the Support Theorem because the diffusion matrix $\mathcal {A}(b)$ introduced in the proof of Proposition 4.10 is not differentiable at b=0. To remedy this fact we ‘avoid’ the singularity by means of a localization procedure. We state the two main results we are going to use for the ease of the reader and refer to [28, Theorem 1.4.5], [6, Chapter 9.5] for details.

#### Proposition B.2

Let $\sigma :\mathbb {R}^n\rightarrow \mathbb {R}^{n\times k}$ be of class $C^2$ with bounded first and second derivative. For $0\le t \le T$, let $X_t$ be the solution of

$$\begin{aligned} dX_t = \sigma (X_t)dW_t, \qquad X_0=x\in \mathbb {R}^n, \end{aligned}$$

then for every $\alpha \in [0, 1/2)$, the support of the measure $P\circ X^{-1}$ in $C^\alpha (0, T; \mathbb {R}^n)$ is the closure of the set $\left\{ S(h), h\in \mathcal {H}\right\}$ where $\mathcal {H}$ is the Cameron-Martin space of *W* and *S*(*h*) is given by the solution of

B.2 $$\begin{aligned} S(h)_t = x + \int _0^t \sigma (S(h)_t)\cdot \dot{h}_tdt - \frac{1}{2}\int _0^t \nabla (\sigma (S(h)_t))\sigma (S(h)_t)dt \end{aligned}$$

#### Proposition B.3

Let $\sigma _i(x):\mathbb {R}^n\rightarrow \mathbb {R}^{n\times k}, \ i=1,2$ be measurable functions and let $X^i$ be the solutions of

$$\begin{aligned} dX^i_t = \sigma _i(X^i_t)dW_t \qquad X^i_0=x_0. \end{aligned}$$

Let $D\subset \mathbb {R}^d$ be an open set such that on *D* it holds $\sigma _1 = \sigma _2$ and

$$\begin{aligned} |\sigma _i(x)- \sigma _i(y)|\le L|x-y|. \end{aligned}$$

Then, if $\tau _i$ denotes the exit time of $X_i$ from *D*, we have

B.3 $$\begin{aligned} \tau _1=\tau _2 \text { a.s. and }\ \mathbb {P}(X_i(t)=X_2(t), \text { for every }0\le t\le \tau _1) = 1. \end{aligned}$$

Recalling the notation introduced in the proof of Proposition 4.10, with these tools we prove the following

#### Proposition B.4

Let $b_t$ be the solution of

B.4 $$\begin{aligned} db_t = \mathcal {A}(b_t)d\hat{W}_t \end{aligned}$$

with $b(0)=b_0\ne 0$. Then for every T>0, ε>0, $b^*\in \mathbb {R}^3$ we have

$$\begin{aligned} \mathbb {P}(|b_T-b^*| \le \varepsilon )>0 \end{aligned}$$

#### Proof

Without loss of generality we can suppose that $$b^*\ne 0$$ (if it does, choose $$|b'|\le \frac{\varepsilon }{2}$$, then use $b^{\prime }$ as target point). Fix $$\delta \le \frac{1}{2}(|b_0|\wedge |b^*|)$$ , $$R\ge 2(|b_0|\vee |b^*|) $$ and call $$D= U_R(0)\smallsetminus \overline{U_{\delta }(0)} $$ the open annulus in $$\mathbb {R}^3$$ of radii δ and *R*. Set $$\tau = \inf \{t>0: b_t \notin D\} $$. Now consider a new diffusion coefficient $$\mathcal {A}^r$$ such that $$\mathcal {A}^r(b)=\mathcal {A}(b)$$ for every b∈D and $$\mathcal {A}^r \in C^\infty _c(\mathbb {R}^3)$$. Finally consider $$b_t^r$$ the solution of $$db_t^r = \mathcal {A}^r(b_t)d\hat{W}_t$$ with $$b_0^r= b_0$$, and $$\tau ^r$$ its associated exit time from *D*. By Proposition B.3, we know that $$\tau = \tau ^r$$ a.s. and $$b_t=b_t^r$$ a.s. for every $$t\le \tau $$. This implies that

$$\begin{aligned} \mathbb {P}(|b_T-b^*| \le \varepsilon )&\ge \mathbb {P}(|b_T-b^*| \le \varepsilon , \ T\le \tau , \ \sup _{t\le T}|b_t-b_t^r|=0)\\&\ge \mathbb {P}(|b^r_T-b^*| \le \varepsilon , \ T\le \tau ^r ). \end{aligned}$$

Let us now suppose that the line $y_t= \frac{t}{T}b^* + \frac{T-t}{T}b_0$ lies inside $D^\varepsilon :=\{x \in D: |x|>\delta + \varepsilon \}$. In that case we have

$$\begin{aligned} \mathbb {P}(|b^r_T-b^*| \le \varepsilon ,\ T\le \tau ^r )&\ge \mathbb {P}(|b^r_T-b^*| \le \varepsilon ,\ T\le \tau ^r,\ \sup _{t\le T}|b_t^r - y_t|\le \varepsilon /2 ) \\&= \mathbb {P}( \sup _{t\le T}|b_t^r - y_t|\le \varepsilon /2 ,\ T\le \tau ^r)\\&= \mathbb {P}( \sup _{t\le T}|b_t^r - y_t|\le \varepsilon /2). \end{aligned}$$

Thus, we are left to show that the curve $y_t$ is in the support of the law of $b^r$ on $C^0(0, T; \mathbb {R}^3)$. But now we are in shape to apply Proposition B.2: we just need to check that there exists $h \in \mathcal {H}$ such that $y_t$ solves the control problem B.2. This is readily done, setting

B.5 $$\begin{aligned} h_t = \int _0^t \mathcal {A}^{-1}(y_t)\left( \frac{b^*- b_0}{T} - (\nabla \mathcal {A}(y_t))\mathcal {A}(y_t)\right) dt \end{aligned}$$

Since $y_t$ has support in *D*, $\mathcal {A}(y_t)$ is invertible with bounded inverse for every t≤T. Also $\mathcal {A}$ and $\nabla \mathcal {A}$ are bounded, thus the integrand in is $L^2$ and $h \in \mathcal {H}$.

If instead $$y_t$$ does not lie inside of *D*, we can find a middle point $$b^{**}$$ such that the line $$\tilde{y}_t$$ connecting $$b_0, b^{**}$$ and $$b^*$$ in time *T* lies inside of $$D^\varepsilon $$ and repeat the argument using $$\tilde{y}_t$$ in place of $$y_t$$. □

To be able to apply Proposition B.1 we need some uniformity of the previous statement with respect to the initial point $x\in \mathbb {T}^3$.

#### Lemma B.5

Suppose that $\overline{B}_0\in C(\mathbb {T}^3; \mathbb {R}^3)$ then for every R>0 the map $\mathbb {T}^3 \ni x\rightarrow \mu _{t,x}(U_R(0))$ is continuous.

#### Proof

Denote $b_t^\nu$ the same process as above with initial condition $\nu \in \mathbb {R}^3$. Since the matrix field $\mathcal {A}$ is Lipschitz continuous and $\overline{B}_0$ is continuous by assumption, $b_t^{\overline{B}_0(x_n)} \rightarrow b_t^{\overline{B}_0(x)}$ a.s. if $x_n \rightarrow x$. The result follows then by dominated convergence recalling that

$$\begin{aligned} \mu _{t,x}(U_R(0))= \mathbb {E}\left[ \boldsymbol{1}_{U_{R}(0)}\left( b_t^{\overline{B}_0(x)}\right) \right] . \end{aligned}$$

□

#### Corollary B.6

If $\overline{B}_0\in V\cap C(\mathbb {T}^3;\mathbb {R}^3)$ and is not identically null, the family of measures $\mu _{t,x}$ satisfy the assumption of Proposition B.1 for any value of R>0.

#### Proof

By Proposition B.4, for every $x_0\in \mathbb {T}^3$ such that $\overline{B}_0(x_0)\ne 0$ and R>0 there exist λ>0 such that $\mu _{t,x_0}(U_R(0)^c)> \lambda$, then by continuity of this quantity with respect to *x* ensured by the Lemma B.5, there exists r>0 such that $\mu _{t,x}(U_R(0)^c)\ge \lambda /2$ for every $x \in U_r(x_0)$. □

## Data Availability

No datasets were generated or analysed during the current study.
